# Evolution of the PWWP-domain encoding genes in the plant and animal lineages

**DOI:** 10.1186/1471-2148-12-101

**Published:** 2012-06-26

**Authors:** Raúl Alvarez-Venegas, Zoya Avramova

**Affiliations:** 1 Department of Genetic Engineering, Centro de Investigación y de Estudios Avanzados, Unidad Irapuato, Irapuato Gto.,36821, Mexico; 2 School of Biological Sciences, University of Nebraska, Lincoln, NE 69588, USA

## Abstract

**Background:**

Conserved domains are recognized as the building blocks of eukaryotic proteins. Domains showing a tendency to occur in diverse combinations (‘promiscuous’ domains) are involved in versatile architectures in proteins with different functions. Current models, based on global-level analyses of domain combinations in multiple genomes, have suggested that the propensity of some domains to associate with other domains in high-level architectures increases with organismal complexity. Alternative models using domain-based phylogenetic trees propose that domains have become promiscuous independently in different lineages through convergent evolution and are, thus, random with no functional or structural preferences. Here we test whether complex protein architectures have occurred by accretion from simpler systems and whether the appearance of multidomain combinations parallels organismal complexity. As a model, we analyze the modular evolution of the PWWP domain and ask whether its appearance in combinations with other domains into multidomain architectures is linked with the occurrence of more complex life-forms. Whether high-level combinations of domains are conserved and transmitted as stable units (cassettes) through evolution is examined in the genomes of plant or metazoan species selected for their established position in the evolution of the respective lineages.

**Results:**

Using the domain-tree approach, we analyze the evolutionary origins and distribution patterns of the promiscuous PWWP domain to understand the principles of its modular evolution and its existence in combination with other domains in higher-level protein architectures. We found that as a single module the PWWP domain occurs only in proteins with a limited, mainly, species-specific distribution. Earlier, it was suggested that domain promiscuity is a fast-changing (volatile) feature shaped by natural selection and that only a few domains retain their promiscuity status throughout evolution. In contrast, our data show that most of the multidomain PWWP combinations in extant multicellular organisms (humans or land plants) are present in their unicellular ancestral relatives suggesting they have been transmitted through evolution as conserved linear arrangements (‘cassettes’). Among the most interesting biologically relevant results is the finding that the genes of the two plant Trithorax family subgroups (*ATX1/2* and *ATX3/4/5*) have different phylogenetic origins. The two subgroups occur together in the earliest land plants *Physcomitrella patens* and *Selaginella moellendorffii.*

**Conclusion:**

Gain/loss of a single PWWP domain is observed throughout evolution reflecting dynamic lineage- or species-specific events. In contrast, higher-level protein architectures involving the PWWP domain have survived as stable arrangements driven by evolutionary descent. The association of PWWP domains with the DNA methyltransferases in *O. tauri* and in the metazoan lineage seems to have occurred independently consistent with convergent evolution. Our results do not support models wherein more complex protein architectures involving the PWWP domain occur with the appearance of more evolutionarily advanced life forms.

## Background

Conserved protein domains appear as a single (the only recognizable) architectural unit or in diverse combinations with a variety of other domains, a feature referred to as “domain versatility” or “promiscuity” [1-3]. Two- or three domains recurring in a conserved linear order within different protein contexts may form “cassettes” with specific functional and spatial relationships [4]. The biological mechanisms that give rise to new domain combinations are largely unknown but novel combinations may arise through the fusion of one protein with another or by the loss/gain of fragments particularly at the proteins’ termini [5-8]. Domain promiscuity is considered a relatively fast-changing (volatile) feature so that only a few domains retain their promiscuity status throughout evolution [1-5]. It is suggested also that formation of multidomain architectures should increase with organismal complexity [9-15] and, thus, the probability of convergent evolution should be low [16]. However, using domain-based trees, instead of a species tree, Forslund et al. [17] found no strong functional bias for multiple independent evolutionary events of protein architecture and suggested that many domains have become promiscuous independently in different lineages unrelated to functionality. Both models are based on studies of multiple genomes from phylogenetically spread species.

Here, we use the domain-based tree approach to analyze the PWWP domain’s evolutionary patterns and to test these opposing hypotheses. The PWWP domain has been identified among the highly promiscuous domains present in proteins involved in various forms of signal transduction, in the ubiquitin system, and in chromatin remodeling [2,12-15]. To be able to infer correlations between species evolution and the occurrences of protein architectural complexity we analyze ancestrally related genomes within the plant and the animal lineages. We test, *first*, whether the complexity of the domain architecture of chromatin proteins has increased during eukaryotic evolution; *second*, whether the assembly of a “versatile” conserved protein domain into multiple-domain architectures is linked to the occurrence of multicellular life-forms; and *third*, whether arrangements involving the PWWP domain represent ancestral *versus* reinvented architectures.

The PWWP domain, discovered in the *WHSC1* (*Wolf-Hirschhorn Syndrome Candidate1*) gene [18] is named for a conserved Pro-Trp-Trp-Pro motif found in eukaryotic, but not prokaryotic, genomes [1]. The domain spans about 70 amino acids and is present in a large number of nuclear proteins involved in cell division, growth and differentiation. The PWWP, Tudor, Chromo (chromatin-binding), and MBT (malignant brain tumor) domains belong to the ‘Royal Family’ of proteins involved in chromatin functions. Three conserved beta-stranded core regions relate them to a common ancestor [19-21]. Emerging evidence implicates PWWP domains in epigenetic regulation through interactions with histones [21-23], with DNA [24-27], or both [28].

By sequence similarity, multiple alignment, and tree-reconstruction approaches, we investigate the phylogenetic relationships among the PWWP domains of proteins from the genomes of species with established evolutionary relationship within the plant and metazoan lineages (see Additional file 1 and Additional file 2) [29,30].

The species with the smallest genome among the free-living eukaryotes *Ostreococcus tauri* (*O. tauri *) is among the earliest members of the green lineage [31]. Here, it provides a model for the minimum of PWWP-domain containing proteins required for life as a free cell. *Chlamydomonas reinhardtii * (called here Chlamydomonas) and *Volvox carteri * (Volvox) were chosen to follow the fate of the PWWP domain in connection with multicellular transitions [32-35]. The moss *Physcomitrella patens * (Physcomitrella) and the lycophyte *Selaginella moellendorffii * (Selaginella) are used as models for the early evolution of the *PWWP * genes in land plants. Physcomitrella is considered a phylogenetic link half-way between algae and angiosperms [36-38] while Selaginella, with no true roots and leaves, occupies an important node of the plant evolutionary tree [39-41]. *Sorghum bicolor* was chosen as a model for a monocot because its genome is highly related to the other grasses, but contains fewer repetitive DNAs than its closest relatives [42]. Since its palaeopolyploidization (approximately 70 million years ago) most duplicated gene sets have lost one member [43] facilitating our analyses by limiting protein redundancy while preserving the PWWP-encoding genes retained for basal monocot functions. *Populus trichocarpaArabidopsis thaliana* and *Arabidopsis lyrata* were analyzed as eudicot models. The need to thrive in fixed locations over centuries under changing environmental conditions and biotic and abiotic stresses sets the poplar apart from the short-lived herbaceous plants. Populus and Arabidopsis lineages have diverged ~100–120 Ma and Populus homologs have been identified for each Arabidopsis gene, but poplar has more (1.4–1.6) protein-coding genes than Arabidopsis [44]. The contribution of gametes from very old individuals accounts for the remarkably reduced rate of sequence evolution. Substantially lower rates of nucleotide substitution, tandem gene duplication, and gross chromosomal rearrangement in Populus than in Arabidopsis have suggested that the poplar genome resembles more closely the ancestral eurosid genome [44].

The evolution of the PWWP-encoding genes in the animal lineage was followed in the genomes of *Homo sapiens* and the cnidarian, sea anemone (*Nematostella vectensis*)*.* The gene number and composition of Nematostella is more similar to vertebrates than are flies and nematodes suggesting that the complexity shared with humans must be ancient [45]. To trace the fate of the *PWWP * domain genes back to their unicellular origins, we analyzed the choanoflagelatum, *Monosiga brevicollis * (Monosiga), sharing a unicellular ancestor with Metazoa more than 600 million years ago [46].

Analyzing a limited selection of ancestrally related genomes positioned at important transitional stages in the evolution of either the plant or metazoan lineages allowed us to ask: 1) whether the modular evolution and the presence of the PWWP domain in complex protein architectures is linked to the occurrence of more complex life-forms; 2) whether assembled PWWP domain combinations are evolutionarily conserved and transmitted as cassettes, and 3) whether the PWWP domain has become promiscuous independently in different lineages through convergent evolution and, thus, the protein architectures are of multiple evolutionary origins and readily reinvented [17].

## Results

### The PWWP containing proteins in the plant lineage

#### The PWWP in unicellular ancestors of green plants: The volvocine algae and O. tauri

To explore the evolutionary history of the PWWP domains in the plant lineage, we analyzed the genomes of the green algae, Chlamydomonas, Volvox *,* and Ostreococcus (see Additional file 1) as they share a common ancestor an estimated one billion years ago [31-36,39]. As novel protein domains and/or combinations are thought to have contributed to multicellularity [9-11], we analyzed the PWWP domain containing proteins in volvocine algae including both unicellular and multicellular species with various levels of morphological and developmental complexity.

**Figure 1:**
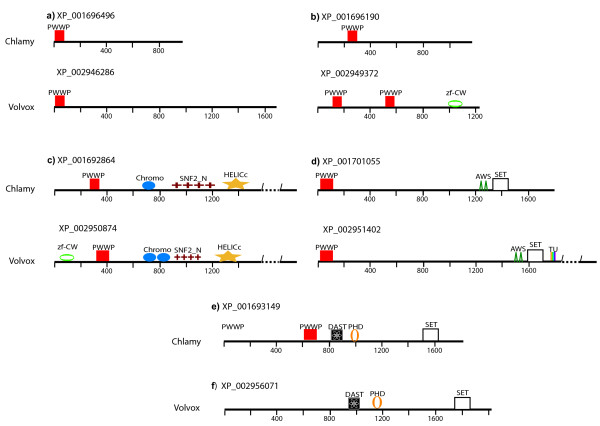
**Domain architecture of the PWWP-containing proteins in Chlamydomonas and Volvox.** Domains in the five Chlamydomonas and four Volvox proteins are drawn to scale. **a**) - **b**) single PWWP-containing proteins that are most similar to each other’s respective homologs. These proteins do not have counterparts in land plants but are highly conserved in extant algal and marine metagenomes; **c**) two algal PWWP containing proteins from the nucleosome modifying SNF2 family. Only the Chlamydomonas/Volvox proteins have ‘gained’ PWWP domains as a volvocine-specific feature; **d**) two PWWP containing proteins from the ASH1 histone methyltransferase family. Only the Chlamydomonas/Volvox proteins have ‘gained’ PWWP domains as a volvocine-specific feature; **e**) the earliest occurrence of the Trithorax (ATX1/2) type architecture with a fully assembled PWWP- DAST-ePHD-SET domain combination in Chlamydomonas. **f**) The PWWP domain has been lost from the Volvox protein.

**Figure 2:**
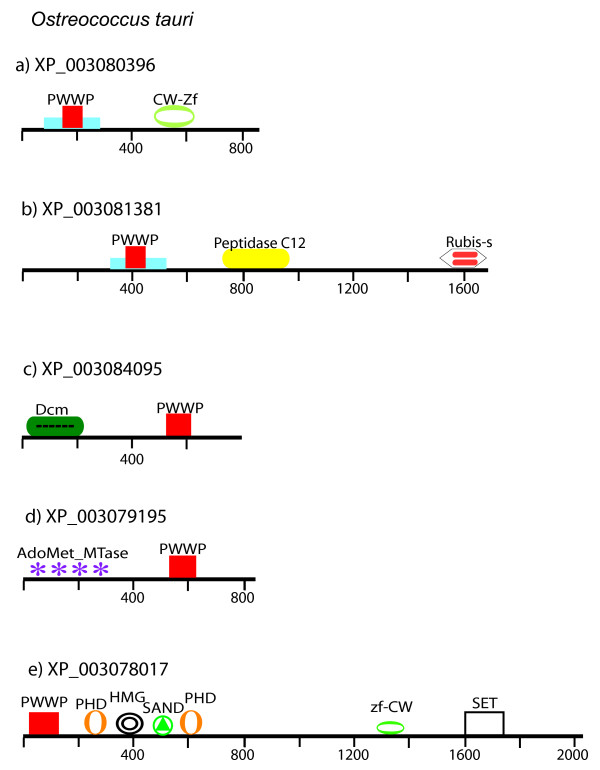
**Domain architecture of the PWWP-containing proteins in *Ostreococcus tauri. *** The five PWWP-containing proteins are drawn to scale. **a**) The PWWP domain (red box) is within a region defined as a Tudor/PWWP/MBT superfamily domain (region in blue) in combination with a domain related to a CW-type zinc finger. Note that this structure is related also to the Set domain fold. **b**) an *O. tauri* specific protein with a similarly organized PWWP domain in a unique combination with an Ubiquitin carboxyl-terminal hydrolase family 1 domain (most similar to ubiquitinases of animal origin) and with a domain from the RuBisCo LSMT substrate-binding protein. The latter domain shows the highest similarity to RuBisCo holoenzyme complex proteins from other algae, but not to the Chlamydomonas, Volvox, or the green plants. This domain is also related to the SET domain carrying a histone methyltransferase activity; **c**) a putative DNA-methyltransferase most similar to proteins from other ocean metagenomes; **d**) a putative DNA-methyltransferase highly similar to the bacterial methyltransferases; **e**) an architecturally complex protein related to the proteins of land plants grouped in the ATX3/4/5 clade but the HMG, the SAND, and the zf-CW domains are absent from the plant versions.

Five PWWP-encoding proteins were identified in Chlamydomonas, four in Volvox, most similar to each other’s respective homologs (Figure 1), and five PWWP-domain proteins in *O. tauri * (Figure 2). Surprisingly, none of the *O. tauri * PWWP proteins showed significant similarity to either the Volvox or PWWP proteins. In all *O. tauri * proteins the PWWP domain is found assembled in high-level architectures (Figure 2). In two of the proteins the PWWP domain appears within a region defined as a Tudor/PWWP/MBT superfamily domain but is combined with different additional domains. In XP_003080396, there is a region of 854 amino acids that is related to the CW-type zinc finger (Figure 2a), while in XP_003081381 the combination is with an Ubiquitin carboxyl-terminal hydrolase family 1 domain (most similar to ubiquitinases of animal origin) and with a domain from the RuBisCo LSMT substrate-binding protein (Figure 2b). It is interesting to note that both the CW-type zinc finger and the Rubis-s domains (see Figure 2a, b) showed structural similarity to the Set domain fold characteristic of the SET domains of histone methyltransferases. We note also that the Rubis-s domain of *O. tauri * shows the highest similarity to RuBisCo holoenzyme complex proteins from other algae but not to the Chlamydomonas, Volvox, or the green plants. The two proteins provide examples of high-level multiple domain assemblies in *O. tauri * that are not found in Chlamydomonas, Volvox, or in the land plants.

PWWP in multidomain combinations found in Volvox and in Chlamydomonas , but not in land plant relatives, are illustrated by two Volvox (XP_002950874 and XP_002951402) and two Chlamydomonas (XP_001692864 and XP_001701055) proteins in Figure 1c, d. These proteins are members of the nucleosome modifying SNF2 and the ASH1 histone methyltransferase families, respectively. Despite highly conserved in land plants, none of the numerous plant members of these families have a PWWP domain. We did not identify PWWP domains in the highly conserved SNF2 and ASH1 family members from *O. lucimarinus*, *O. tauri,* or *Micromonas sp *. either, but a divergent (EFWPA) domain is present in an SNF2 protein from *Chlorella variabilis.* The results suggest that some algal SNF2 and ASH1 proteins have ‘gained’ a PWWP domain as species-specific features.

Two solo PWWP domain containing proteins in Chlamydomonas (XP_001696496, XP_001696190) are highly related to the Volvox (XP_002946286, XP_002949372, note the duplicated PWWP motif in the latter, Figure 1a, b) and to proteins in other algal and marine metagenomes (i.e., *Chlorella variabilis, Micromonas pusilla, O. lucimarinus* and *O. tauri*). Interestingly, these proteins have no similar counterparts in the land plants but are closely related to bacterial proteins (without the PWWP domain) suggesting common origins. In the unicellular marine eukaryotes the PWWP domain has apparently been ‘gained’ for algal specific functions.

**Figure 3:**
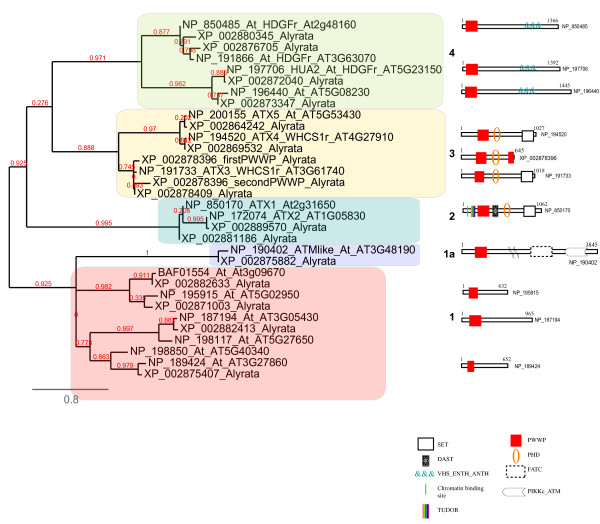
**Likelihood phylogeny of PWWP containing proteins in *A. thaliana *and *A. lyrata. figure 4 *** PWWP-domain sequences with the highest BLAST scores were used to generate the Maximum Likelihood phylogenetic tree. The distinct PWWP-domain subgroups (as discussed in the main text) are indicated by numbers and shaded in different colors. Domain names and representative domain architecture are shown for the major PWWP-domain protein families. Species abbreviations correspond to *A. thaliana* (At) and *A. lyrata * (Alyrata). The branch length is proportional to the number of substitutions per site. Numbers in red correspond to the branch support values. The FATC and the PIKKc_ATM domains in the ATM proteins represent the FRAP, ATM, TRRAP C-terminal domain and the phosphoinositide 3-kinase-related protein kinase_Ataxia telangiectasia mutated (ATM), catalytic domain, respectively.

**Figure 4:**
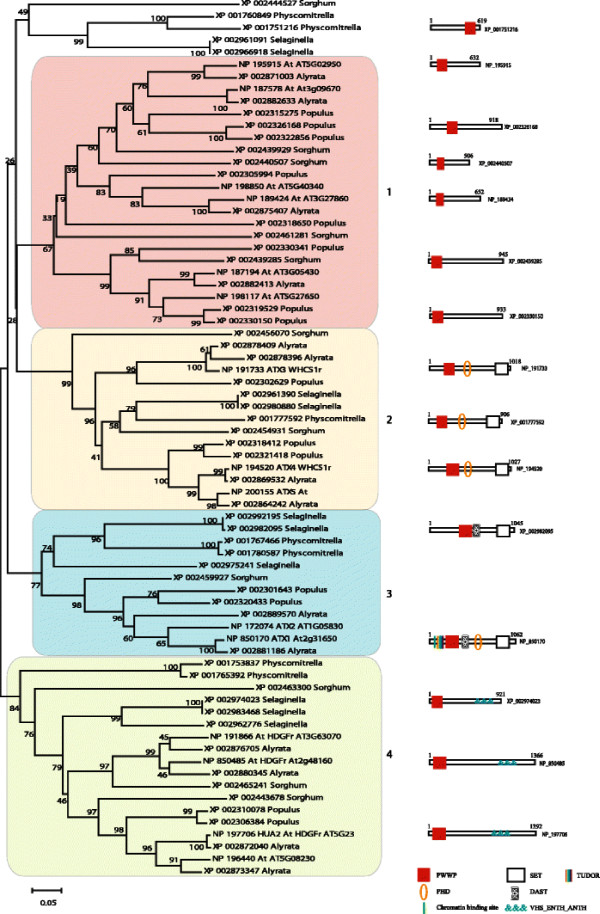
**Neighbor-Joining phylogeny of PWWP containing proteins in plants.** The evolutionary history was inferred using the Neighbor-Joining method. The percentage of replicate trees in which the associated taxa clustered together in the bootstrap test (5000 replicates) are shown next to the branches. The tree is drawn to scale, with branch lengths in the same units as those of the evolutionary distances used to infer the phylogenetic tree. All positions containing gaps and missing data were eliminated only in pairwise sequence comparisons (Pairwise deletion). Phylogenetic analyses were conducted in MEGA4. The distinct PWWP-domain subgroups (as discussed in the main text) are indicated by numbers and shaded in different colors. Domain names and representative domain architecture are shown for the major PWWP-domain protein families.

The relationship uncovered between the Chlamydomonas protein XP_001693149, the *O. tauri*, XP_003078017, and the land plant proteins from the ATX1/2 and ATX3/4/5 subgroups of the trithorax family are among the most revealing findings of this study. The Chlamydomonas protein (Figure 1e) contains the PWWP, the FYRN-FYRC (DAST), ePHD, and SET domains in the linear arrangement characteristic for the plant ATX1/2 proteins (Figure 3, clades 2,3; Figure 4, clade2,3). Phylogenetically, each of the architectural domains of the Chlamydomonas protein is most closely related to the respective domains in plants defining the Chlamydomonas protein as the earliest ancestral relative of the land plants’ ATX1/2. Presence of PWWP in a linear combination with DAST-ePHD-SET domains is a signature feature of the plant Trithorax family setting it apart from animal Trithorax proteins [47]. Notably, the PWWP domain is missing from the highly related Volvox ATX1/2 counterpart (Figure 1f), most likely, being lost during its separation from Chlamydomonas.

A PWWP domain, associated with an ePHD, and SET domains, but lacking the DAST domain, is present also in *O. tauri* (Figure 2e). Phylogenetically, however, the domains from the *O. tauri* protein are related to the plant proteins of the ATX3/4/5 subgroup but not to any of the Chlamydomonas, or to the plant Trithorax (ATX1/2), proteins. Thereby, the *O. tauri* protein XP_003078017 represents an ATX3-architype and the earliest ATX3 predecessor (among available sequenced genomes). It carries all structural domains in a linear order conserved in land plants suggesting that it has been transmitted as a stable unit through evolution.

This analysis provided important evidence for the different phylogenetic origins of the two subgroups of the plant Trithorax family. While the linear arrangement of the PWWP-DAST-ePHD-SET domains in the Chlamydomonas protein is an early version of the multidomain architecture of the plant ATX1/2, the PWWP-PHD-ePHD-SET linear arrangement in *O. tauri* represents the earliest version of the ATX3/4/5 trithorax subtypes. These linear arrangements have been transmitted as stable, not volatile, combinations through green plants’ evolution. In addition to the conserved cassettes, more domains are present on the respective algal proteins (Figure 1c, Figure 2e) indicating that multidomain combinations involving the versatile PWWP domain have been assembled in the unicellular ancestor of plants and, thus, complexities of protein architectures do not correlate with organismal complexity.

It is important to note also that the two *O. tauri PWWP* genes encoding putative DNA-methyltransferases (Figure 2 c, d) are found in Micromonas, Chlorella, and other ocean metagenomes, but not in Chlamydomonas/Volvox or in the land plants genomes [48]. A PWWP domain in combination with DNA-methyltransferase activity is a characteristic feature of the metazoan DNA-methyltransferases, however. Excluding the PWWP domain, the other *O. tauri* DNA-methyltransferase (XP_003079195 in Figure 2d) is most related to the bacterial methyltransferases [48].

### The PWWP during land plants’ evolution

The colonization of land by plants has been associated with substantial changes in morphology, as well as in cellular and physiological regulatory processes to resist heat, cold, desiccation, and UV-induced DNA-damaging effects. Some molecular clocks set the separation of mosses and vascular plants at ~ 700 Ma [38-41], while lycophytes have diverged from the fern/seed plant lineage at least 400 million years ago [40]. Whole-genome searches of the model species here identified 7 genes in Physcomitrella, 10 in Selaginella, 10 in *S. bicolor*, 15 in P. trichocarpa, 16 in *A. thaliana* and 14 in *A. lyrata* encoding putative PWWP-domain proteins (see Additional file 3). Here, we analyze the distribution and the phylogenetic relationship of the PWWP domain containing proteins of land plants to assess whether it appears as inherited assemblies, as single domain acquisitions/deletions, or as entirely novel occurrences in plant-specific combinations.

#### Arabidopsis thaliana and Arabidopsis lyrata

The eudicot *A. thaliana,* with a relatively small genome, is routinely used as the choice model for key comparisons with other plant genomes. Using the PWWP domain sequence of ATX1 (ARABIDOPSIS HOMOLOG OF TRITHORAX1) as a probe [49], we identified 16 genes encoding PWWP-containing proteins in the genome of *A. thaliana* (see Additional file 3). Based on the similarity of the PWWP amino acid sequences, two types of trees, ML and NJ-trees, were constructed. Both approaches yielded identical distribution patterns of the proteins within the subgroups (see Additional file 4Additional file 5 and Additional file 6) supporting the phylogenetic relationships between the PWWP-containing proteins of *A. thaliana*. These relationships were further confirmed by analyses of the PWWP-containing proteins of *A. lyrata*. As a very close relative [50-52], the genome of *A. lyrata* provides a well-suited system for distinguishing paralogs (the members of the same clade) from orthologs (the members of the other clades) and for revealing correlations between whole-protein structures and their segregation into specific subgroups. Fourteen genes encode PWWP-containing proteins in *A. lyrata* (see Additional file 3). Phylogenetic analysis positioned them into the same clades formed by the *A. thaliana* proteins (Figure 3) thus confirming the relatedness among the paralogs or the orthologs within each cluster. The gene encoding the *A. lyrata* protein XP_002878396 lacks an apparent homolog in *A. thaliana* and contains two closely related PWWP domains (Figure 3, clade 3) representing an *A. lyrata* species-specific gene. Another difference, illustrated by the presence of six *A. thaliana,* but only four *A. lyrata* paralogs in clade 1 (Figure 3) suggested that a duplication/deletion event has taken place after the separation of the two species.

Analyses of the proteins within each clade revealed characteristic features. Thus, all proteins of clade 1 have a solo PWWP domain at the N-terminal regions as the only recognized architectural module. Outside the PWWP domain, the proteins are most similar to each other indicating that similarities of the PWWP sequences reflect the phylogenetic relationships among the whole proteins. The remarkable conservation of these proteins in land plants (see below) and the proliferation of their gene numbers imply strong functional relevance, although no function for any protein from this subgroup has been reported.

Related to clade 1 are the PWWP domains of two proteins from the ATM (ATAXIA-TELANGIECTASIA MUTATED) family (Figure 3, clade 1a). The characteristic feature of these proteins is the combination of the PWWP domains with the ATM catalytic domains. Members of the ATM family carry phosphoinositide 3-kinase-related (PI3K) activity and are critical for chromosome stability and for the response to DNA double strand breaks caused by irradiation [53]. All land plants examined here, including Physcomitrella and Selaginella, have several highly conserved genes encoding a putative ATM activity but, notably, none of these plant proteins carries a PWWP domain. Apparently, the PWWP has been co-opted by the *ATM* gene in the ancestor of the Arabidopsis lineage after its separation from the other plants. These proteins were not included in further tree reconstructions.

Clades 2 and 3 are remarkable in that they contain the five proteins from the Arabidopsis Trithorax-like (ATX) family. The PWWP-domain based tree approach used here segregated the proteins into the same two subgroups established earlier by the relatedness of their SET (Suppressor of variegation, Enhancer of zeste, Trithorax) domains [54,55]. Thereby, in addition to the differences in their SET domains and the presence/absence of the FYRN/FYRC domain (also called DAST [49]), the members of the two ATX subgroups differ also by the nature of their PWWP domains: ATX1 and ATX2 group together, while the ATX3, ATX4 and ATX5 form a separate subgroup suggesting non-monophyletic origins (Figures 3,4, and see Additional file 7 and Additional file 8). The results from the analysis of unicellular algae (see above) provided strong support to the hypothesis that the separation of ATX1/2 from ATX3/4/5 is ancient and that the two subgroups are of apparently distinct phylogenetic origins.

The last cluster (4) contains PWWP domains in combination with the VHS_ENTH_ANTH domains. The VHS, ENTH and ANTH domains are structurally similar, can bind inositol phospholipids, and are found in proteins involved in the nucleation and formation of clathrin-coated vesicles [56]. The only studied member, the *A. thaliana* protein NP_197706, HUA2, contains a nuclear localization signal and may act as a transcription factor [57]. *HUA2* genetically facilitates the *AGAMOUS, AG,* gene [57-59] but the molecular role of the PWWP domain in HUA2 function has not been established.

### The PWWP domain proteins in *P. trichocarpa*, *S. bicolor, P. patens* and *S. moellendorffii*

Phylogenetic analyses of the PWWP domain containing proteins from the other model land plants positioned them into the same clades, as defined by the Arabidopsis proteins (Figure 4), suggesting common origins for the plants’ PWWP-containing orthologous groups. However, in different species different numbers of genes encode the members of each clade. For example, eight genes in *P. trichocarpa*, four in *S. bicolor*, six in *A. thaliana,* and four in *A*. *lyrata* encode the proteins in clade 1 of Figure 4 (see also Additional file 8). Two solo PWWP-encoding genes from Selaginella, two from Physcomitrella, and one *S. bicolor* are weakly related to the proteins of clade 1 (Figure 4). Even in the two closely related Arabidopsis species these genes are present in a different copy-number suggesting that the encoded proteins play species-specific roles. This is the most abundant type of PWWP-containing proteins suggesting that the solo PWWP-encoding genes have important plant functions, although none has been established yet (see Additional file 7).

The proteins of clusters 2 and 3 (Figure 4) are members of the trithorax related ATX3/4/5 and ATX1/ATX2 subgroups, respectively. The phylogenetic relationship between the PWWP domains of the angiosperm trithorax-like proteins is supported by their architectural content (see Additional file 7 and Additional file 8) as well as by the relatedness of their SET domains [47,54]. We note that the two (ATX4 and ATX5) proteins from *P. trichocarpa* and the Arabidopsis form a subgroup that is distinct, although related, to the ATX3 subgroup and that the proteins from Physcomitrella and Selaginella group separately in a cluster related to the ATX4/5 subgroup in clade 3. We also note that the different plant genomes contain different numbers of proteins belonging to either the ATX3/4/5 or ATX1/ATX2 subgroups suggesting that specific duplication events multiplying members of these subgroups have taken place in individual plant’s genomes.

Clade 4 contains the PWWP- VHS_ENTH_ANTH containing proteins (see above). Four genes in Arabidopsis, three in *S. bicolor*, and two in *P. trichocarpa* encode members of this clade. Given the role of the *A. thaliana* HUA2 in flower development [57-59], it is remarkable that there are PWWP- VHS_ENTH_ANTH encoding genes in Physcomitrella and in Selaginella indicating roles different from involvement in flowering.

Collectively, the analyses of the PWWP domain containing proteins in land plants indicate that: 1) all PWWP-containing proteins are phylogenetically related consistent with common ancestral origins; 2) the solo PWWP domain-containing proteins, only weakly related to the solo PWWP domain proteins of Chlamydomonas/Volvox, are the most abundant indicating a specific proliferation of this group in the land plants; 3) the ancestral version of ATX3, found in *O. tauri,* and of ATX1, found in Chlamydomonas, illustrate the transmission of complex domain arrangements as stable units. Copies of these genes, appearing together for the first time in the moss, are present in all examined here land plants in species-specific copy numbers; 4) all PWWP domain cassettes found in land plants are found also in unicellular algae suggesting they have been inherited from the common ancestor. A notable exception are the PWWP- VHS_ENTH_ANTH containing proteins as no related proteins were found in the available genomes of unicellular organisms. These proteins may have occurred as a function specific for the land plants.

### The PWWP proteins during the evolution of the animal lineage

#### The PWWP-genes in Monosiga brevicollis

**Figure 5:**
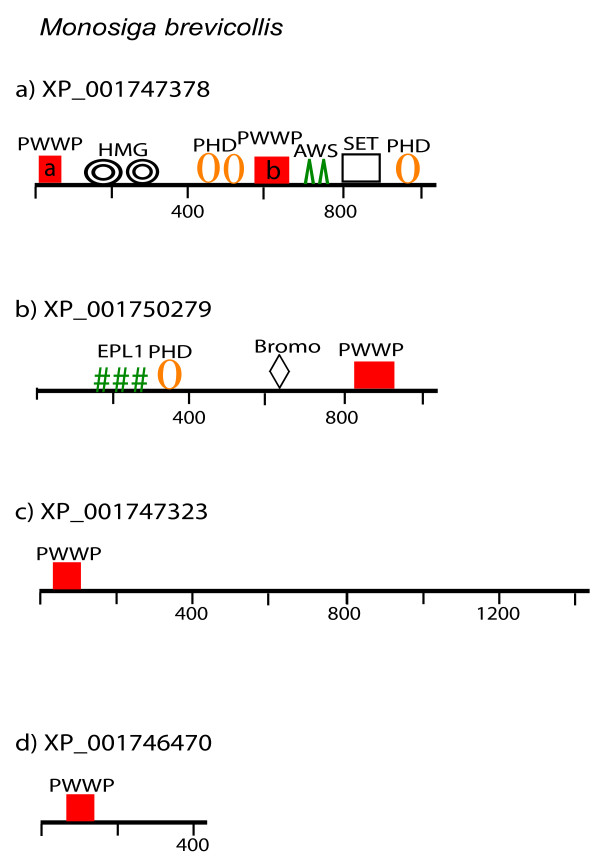
**Domain architecture of the PWWP-containing proteins in *Monosiga brevicollis *. a**) the multi-domain protein containing two PWWP (labeled boxes ‘a’ and ‘b’), three PHD, an HMG, AWS and SET-domains has been conserved in the metazoan proteins although the different human copies have lost either the HMG, or the terminal PHD domains (see Figure 6); **b**) a Peregrin-type protein with the characteristic cassette involving EPL1 (a not well-defined domain), a PHD, Bromo, and PWWP domains. It represents the earliest version and a founding member of the BRPF-family (see Figure 6); **c**) a Monosiga-specific protein. The PWWP-domain shows low-level similarity with the PWWP domain of animal HDGF proteins; **d**) a single PWWP domain containing Monosiga-specific protein. Domains are drawn to scale. EPL1 is the Enhancer of polycomb-like domain.

The unicellular choanoflagellate *Monosiga brevicolis* is considered the closest known relative of metazoans [46]. Because genomic features shared by Monosiga and metazoans were probably present in their last common ancestor, we extended the evolutionary history of the PWWP domains to the pre-metazoan era. The architecture of the four *M. brevicollis* PWWP-domain proteins is shown in Figure 5. Two of the proteins carry a PWWP as the only recognized domain and are, likely, Monosiga-specific as no significant similarity to any protein in the *NCBI* database was revealed. Low-level similarity limited to the PWWP domains is displayed by the Monosiga protein XP_001747323 and the animal HDGF (Hepatoma derived growth factor), a nuclear protein with mitogenic activity [60].

The other two proteins (Figure 5 a, b) are of a particular relevance for our model as they represent complex multi-domain arrangements that have been conserved in evolution. One of the *M. brevicollis * proteins (XP_001747378) carries two PWWP domains, two HMG boxes, and three PHD fingers associated with AWS-SET domains as the earliest recognized version of the NSD (WHSC) family members (Figure 5a). The SET domain, the signature feature for the NSD family, relates it to the superfamily of the histone lysine methyltransferases. The two PWWP domains (labeled PWWPa and PWWPb) are conserved also in the human genes (see further below). Notably, however, the human versions represent losses, rather than acquisitions, of additional architectural domains, as seen by the deletion from the human proteins of either the HMG-boxes (in WHSC1L1 NM_023034) or the C-terminal PHD in WHSC1 NM_133330 (see Additional file 7 and Additional file 9).

The other multidomain protein of Monosiga shows a remarkable conservation of domains and of their linear arrangement with members of the animal Peregrin family (Figure 5b). Phylogenetically, the Monosiga protein clusters with the human Peregrin-type proteins (see Additional file 9) suggesting that it represents an ancient PWWP-combination that has been assembled in the common ancestor and conserved in the Monosiga and metazoan relatives.

Thereby, contrary to the suggestion that abundant domain shuffling followed the separation of the choanoflagellate and metazoan lineages [46], two of the four PWWP containing proteins provide examples of complex multi-domain linear combinations that have been transmitted to the human genomes.

### The PWWP-genes in the sea anemone *Nematostella vectensis* and in humans

Sea anemones constitute the oldest eumetazoan phylum and the sequenced genome of *Nematostella vectensis* indicated many genes uniquely shared with animals [45]. Among the 18,000 protein-coding Nematostella genes, six encode PWWP domain containing proteins. Here, we compare their evolution to the metazoan (human) lineage.

**Figure 6:**
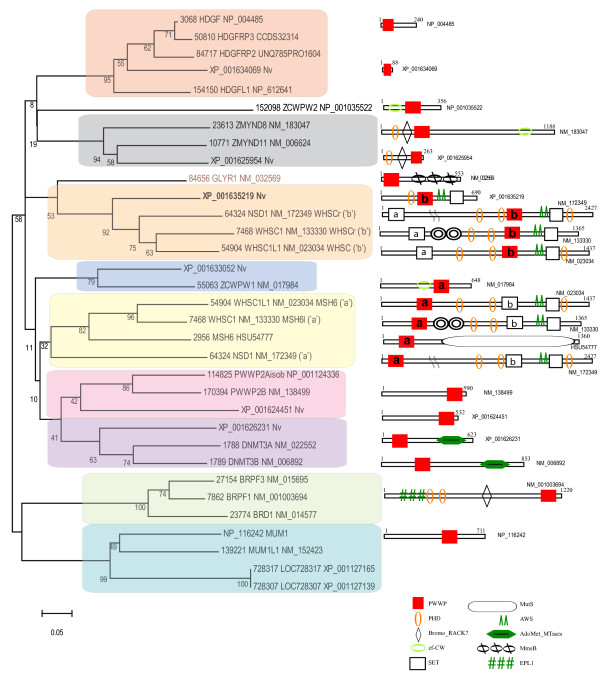
**Neighbor-Joining phylogeny of PWWP containing proteins in the Sea Anemone *Nematostella vectensis * and in humans.** The evolutionary history was inferred using the Neighbor-Joining method. The percentage of replicate trees in which the associated taxa clustered together in the bootstrap test (5000 replicates) are shown next to the branches. The tree is drawn to scale, with branch lengths in the same units as those of the evolutionary distances used to infer the phylogenetic tree. All positions containing gaps and missing data were eliminated from the dataset (Complete deletion option). Phylogenetic analyses were conducted in MEGA4. The full-size protein products of the three genes from the NSD (WHSC) family are included in the analysis. Each of the three proteins appears twice (annotated with an ‘a’ or ‘b’ proteins) in two separate clades on the phylogenetic tree, as the two PWWP domains of each protein form separate clusters (see text for details). The distinct PWWP-domain subgroups (as discussed in the main text) are shaded in different colors. Species abbreviations correspond to *Nematostella vectensis* (Nv) and the rest of the proteins correspond to humans.

In the human genome, 24 genes encode more than 70 PWWP containing proteins that form distinct phylogenetic clusters (Figure 6, clades 3 and 5). Three genes (NM_133330, NM_023034, and NM_172349) encode about 40 proteins from the NSD (WHSC) family representing differentially spliced isoforms. In addition to the signature SET domain, presence of two PWWP domains (marked as PWWP ‘a’ and PWW ‘b’) is a characteristic feature for the NSD (WHSC) family. Phylogenetic analysis indicated that ‘a’ and ‘b’ PWW domains form different phylogenetic subgroups suggesting different origins and that the NSD (WHSC) proteins may have resulted from fusion of two different PWWP domain containing proteins. Such a scenario is supported by the Nematostella protein XP_001635219 (Figure 6, shown in bold) where a PWWP of the subtype ‘b’, exists in combination with the PHD, AWS, and SET domains exactly as found in the cassette conserved at the C-terminal-half of the metazoan NSD proteins. Furthermore, the N-terminal portions of the human NSD proteins contain a PWWP domain (of type ‘a’) that is related to a different protein subgroup that includes the Nematostella (XP_001633052) and the human ZCWPW1 proteins (Figure 6, clade 4). In addition to type ‘a’ *PWWP* domain-sequences, the latter two genes carry also the *zf-CW* domain sequences. It is remarkable that the two N-terminal and C-terminal PWWP-carrying cassettes are found fused together in the unicellular Monosiga (Figure 5a; SF 6) and have been propagated as a unit to the metazoan lineage. In Nematostella, the N-terminal cassette apparently, has been deleted from the NSD related protein.

The PWWP domain of the human mismatch repair protein MSH6 (HSU54777) also belongs to the type ‘a’ and the presence of a PWWP domain in MSH6 is an exclusively metazoan feature, as a similar combination has not been found in any other mismatch repair in the examined genomes. The PWWP domain of the human GLYR1 (glyoxylate reductase 1 homolog of Arabidopsis) is phylogenetically related to the ‘b’-type. Despite highly similar to the plant glyoxylate reductase, only the metazoan proteins carry a PWWP domain. The cnidarian homologous proteins do not carry PWWP-domains suggesting that the PWWP-domain was gained after the metazoan divergence from Nematostella.

Two cassettes containing a PWWP-PHD and BROMO domains are found in the ZMYND8/11 and Peregrin (BRPF) families. Despite similar modular structures, the PWWP domains from the two cassettes distribute into different subgroups (Figure 6, clades 2 and 8) suggesting phylogenetically distinct origins. Nematostella has one protein belonging to the ZMYND8/11 group but no Peregrin homolog.

An interesting case of related PWWP domain containing proteins is presented by the four human proteins in Figure 6 (clades 6 and 7). One subset carries only the PWWP domain, the other group represents a combination of PWWP with DNA methyltransferase activity. Such a combination is not found in Monosiga but is present in *O. tauri* (Figure 4) [48]. Despite the architectural similarity, however, the PWWP domains of the *O. tauri,* Nematostella or human DNA methyltransferases do not appear to be phylogenetically related (see Additional file 9) suggesting independent occurrence of the PWWP-DNA methyltransferase association.

Lastly, two separate clades represent proteins with a single PWWP domain: the HDGF and the MUM1/EXPAND1 (clades 1 and 9 in Figure 6), respectively. The two human proteins XP_001127165 and XP_001127139 are predicted, containing a PWWP domain highly related to the MUM1 subgroup and a domain found in the APC family. Only the HDGF cluster contains a Nematostella protein.

Collectively, our analyses of the PWWP domain containing proteins in the metazoan lineage suggested that: 1) the higher-level architectural PWWP assemblies found in the unicellular *M. brevicollis* genes have been transmitted as stable combinations to the human genome. However, only one of the Monosiga proteins, the WHSC-related protein (Figure 5a) is found in Nematostella where it is represented by two separate proteins of the subtypes ‘a’ and ‘b’ (Figure 6); the gene encoding a homolog of the Peregrin family is not present in Nematostella; 2) like in the plant lineage, the single PWWP domain proteins are species-specific, except for the HDGF proteins, which contains proteins from both the human and the cnidarian genomes suggesting ancient origins of these proteins (clade 1 in Figure 6); 3) the association of a PWWP domain with a DNA-methyltransferase activity is found in the human and cnidarian genomes, but not in *M. brevicollis*; 4) the combinations of the PWWP domain with the mismatch repair function (in HSU54777 protein), and with the glyoxylate reductase 1 homolog (in the GLYR1 protein) appear to be metazoan-specific.

## Discussion

The evolution of complex protein architectures has been linked to the requirements for novel functions during the occurrence of multicellular life-forms [1,2]. Here, we test whether the architectures identified in the genomes are of evolutionary descent, consistent with low probabilities of convergent evolution [16], or whether the PWWP domain has become promiscuous independently in different lineages and, thus, consistent with convergent evolution [17]. Analyzing the distribution patterns of the promiscuous PWWP domain in ancestrally related genomes from the plant and metazoan lineages we follow the tendencies for the PWWP domain to appear as a single module, in species-specific multidomain arrangements, and as evolutionarily conserved cassettes transmitted as stable units.

### The PWWP domain as a single module

As a single domain, PWWP is found in proteins encoded by both unicellular and multicellular genomes. However, the proteins found in unicellular organisms are not found in the multicellular and *vice versa*. Thereby, a characteristic feature of single-PWWP domain proteins is that they are of different phylogenetic origins and with limited distribution among closely related species. Examples are the single PWWP domain proteins of *M. brevicolis* and from Chlamydomonas/Volvox that have homologs in other marine metagenomes but are not conserved in the animal or plant lineages. Interestingly, the existence of highly similar proteins of bacterial origin, but lacking the PWWP domain, indicate that marine unicellular eukaryotes have ‘gained’ a PWWP domain for algal specific functions. No solo PWWP domain encoding genes were found in *O. tauri* (see Additional file 7).

Genes encoding single PWWP domain proteins in multicellular genomes also display limited lineage-specific distribution. In land plants, such proteins are clustered in clade 1 (Figure 4). Although functions have not been established for any member of this subgroup, they are likely plant-specific. The single PWWP domain proteins in the animal lineage form two distinct clades suggesting different phylogenetic origins (Figure 6, clades 1 and 9). The proteins in clades 1 and 6 (Figure 6) contain one Nematostella representative but the MUM1/EXPAND1 members have no PWWP domain homologs in the cnidarian or in *M. brevicollis* genomes.

### The PWWP domain in species- or lineage-specific multidomain arrangements

The PWWP module found in various combinations with other domains may also be species- or lineage-specific. In unicellular genomes examples are the two *O. tauri* proteins (Figure 2a, b; see Additional file 7) where multiple domains are assembled in a complex architecture that has not been found in multicellular organisms. Individual modules from these proteins, however, have been passed on to multicellular lineages: i.e., the Ubiquitin carboxyl-terminal hydrolase domain (most related to ubiquitinases of animal origin) or the RuBisCo sequences (conserved in plants), illustrate multidomain assemblies in architectures that are more complex in the unicellular than in the multicellular lineages. Acquisitions (accretion) of domains are illustrated by the ‘gained’ PWWP domains in the Chlamydomonas/Volvox SNF2 and ASH1 proteins (Figure 1 c, d), not present in the multicellular relatives, illustrating higher-order architectures in the algal than in plant proteins. Furthermore, the presence of a PWWP in the ATX1 homolog of Chlamydomonas but lost in Volvox (Figure 1 e, f) negatively correlates with the complexity of this protein architecture and with the ability of this species to transition to multicellular forms.

Gain or loss of a PWWP, usually at the N-termini, is found in proteins of both unicellular and multicellular organisms as a species- or lineage-specific trait (see Additional file 7). For example, we found that the human mismatch repair protein MSH6 and GLYR1 (glyoxylate reductase 1 homolog of Arabidopsis) have a PWWP domain only in the metazoan, but not plant, proteins. Furthermore, clade 4 (in Figure 4) contains plant proteins with an apparently lineage-specific acquisition of an N-terminal PWWP domain by proteins participating in intracellular membrane trafficking, while the Arabidopsis ATM proteins (Figure 3, clade 1a) provide an example of an Arabidopsis-specific ‘gain’ of *PWWP* sequences by the *ATM-PI3K* genes upon the divergence of the Arabidopsis species from the eudicots.

Collectively, these findings suggest that gain/loss of the PWWP domain from the termini of existing proteins is a recurring feature in the evolution occurring in a lineage or species-specific pattern that does not support a model postulating that complex architectures in multicellular systems occur by accretion from simpler systems [9,10,61].

### The PWWP domains in evolutionarily conserved cassettes

Complex multidomain arrangements of PWWP with other domains have been found as stable cassettes transferred through unicellular/multicellular evolutionary transitions (see Additional file 7). A Peregrin-type protein with the characteristic cassette involving the EPL1-PHD- BROMO-PWWP domains (in a rare C terminus location) is seen assembled in Monosiga, lost in Nematostella, but re-appearing in humans as a small gene family (see Additional file 7 and Additional file 9). The Brf1 protein, identified as a TrxG protein with essential roles in epigenetic memory during vertebrate development [62], raises the immediate question about its possible role in the unicellular Monosiga. A different evolutionary path, however, was revealed for a similar cassette containing the same (PHD- BROMO-PWWP) modules, but of a phylogenetically different origin: assembled together, the cassette is present in the cnidarian, inherited in humans (the ZMYND8/11 proteins) (Figure 6), but not found in the unicellular organisms or in plants. The earliest relatives of the human ZCWPW1/ ZCWPW2 and ZMYND8/11 PWWP domain proteins are shared with the cnidarians and, thus, might reflect specific combinations related to multicellular development.

The NSD protein types provide a striking example of a complex multidomain arrangement present in an assembled combination in Monosiga and found in humans (Figure 5a, Figure 6, and see Additional file 7 and Additional file 9). These proteins contain two PWWP domains (PWWP ‘a’ and PWWP ‘b’) of different phylogenetic origin and in association with different domains. Each of the PWWP ‘a’ and PWWP ‘b’ seems to represent a distinct cassette (as illustrated by the two separate proteins in Nematostella) but fused into one gene in Monosiga (Figure 5a). Three ‘fused’ genes in the human genome encode more than 40 proteins as distinct isoforms. Plant proteins belonging to the NSD family have not been identified although a PWWP-AWS-SET domain in an arrangement similar to the C-terminal part of NSD proteins are found in the Volvox/Chlamydomonas ASH1-like proteins (Figure 1d). The NSD proteins illustrate complex multidomain arrangements of PWWP with other domains in stable combinations that have been preserved through evolutionary transitions and inherited from unicellular to multicellular lineages as stable units.

The associations of PWWP with DNA methyltransferase activities in the two *O. tauri* proteins, in Nematostella, and in humans provide an interesting example consistent with a convergent evolution and re-invented architectures [17]. Despite the similar combination, the PWWP domain in the *O. tauri* proteins is at the C-terminal end, while in the human and cnidarian proteins the PWWP domain is at the N-terminus. Importantly, our phylogenetic analysis did not support a relationship between the metazoan and *O. tauri* proteins suggesting distinct phylogenetic origins. Excluding the PWWP domain the *O. tauri* protein XP_003079195 is most similar to the bacterial methyltransferases, while XP_003084095 including the PWWP domain is most similar to the putative DNA-methyltransferases from *MicromonasChlorella*, and other ocean metagenomes [48]. Notably, the DNA methyltransferases from the genomes of Chlamydomonas/Volvox, land plants, and Monosiga do not have PWWP domains.

### Evolutionarily distinct origins of the plant *ATX1/2* and *ATX3/4/5 trithorax*-like genes

An unexpected finding of this study was that the *ATX1/2* and *ATX3/4/5* gene types in plants are of a different origin. The relatedness of their PWWP domains positioned the proteins into two separate clades, consistent with their segregation based of the relatedness of their SET domains [47,54]. Analyses of unicellular algal genomes revealed that a fully assembled ATX1/2 (cassette) was present in Chlamydomonas (Figure 1), while the PWWP-ePHD-SET cassette of the ATX3 type has been assembled in the common ancestor with *O. tauri* (Figure 2). The earliest simultaneous presence of both protein types in land plants is found in Physcomitrella (Figure 4) where the *ATX3*-like gene is found as a single copy but the *ATX1* has been duplicated. Different numbers of the *ATX3*-like or *ATX1*-like gene duplications have occurred in individual plant genomes, as seen in Figure 4; see Additional file 8).

The ATX3/4/5 type proteins are plant-specific, while the ATX1/2 proteins are related to the metazoan MLL, except for the presence of the PWWP domain in the plant trithorax proteins. In Arabidopsis, the *ATX1* and *ATX2* genes have originated from a chromosomal segmental duplication but have evolved divergent biochemical activities as features on the path of neofunctionalization [63]. It will be informative to reveal the roles of the duplicated *ATX1/2* genes in other plant species, particularly in the earliest land plants, to reveal the basal functions of the ancestral genes.

### Evolution and diversification of the PWWP domain function

The PWWP domain is defined as a chromatin-binding domain with dual functions binding both DNA and methyl lysine histones [21-28,64-67]. The third (Trp) and fourth (Pro) residues of the PWWP motif are highly conserved but the other residues may vary, illustrating specific divergences that may underlie different functions. The NMR structures of the PWWP domains from different proteins show a high-degree similarity and common topology, although the C-terminal regions of the PWWP domains are significantly divergent [68]. The structure of the PWWP domain in complex with bound substrates has been reported [28,69] and we shall not discuss it here. It is interesting to note, however, that the PWWP domain of LEDGF is critical for the function of the MLL in chromatin [70] and that the functional association of the LEDGF-PWWP domain with MLL at *Hox* genes mimics the naturally occurring arrangement of a PWWP domain at the N-terminus of the plant trithorax homologs, ATX1/2. These results provide evolutionary support for a functional link between MLL and a PWWP. Although promoting the association of MLL with chromatin, the molecular role of the PWWP domain in the H3K4 trimethylation by either the human MLL or by the plant ATX1 remains unknown.

Given the established roles for the PWWP domains in chromatin, the finding of a PWWP domain linked to modules implicated in non-nuclear functions is intriguing. For example, the Ubiquitin carboxyl-terminal hydrolase-RuBisCo domains in *O. tauri*, the human GLYR1 involved in lipid metabolism and the cellular redox homeostasis, and the plant proteins associated with the VHS-ENTH-ANTH domains implicated in cellular trafficking and membrane functions, raises interesting questions about roles that seem unrelated to chromatin. The role of HUA2 as a transcription factor in the nucleus [57], the binding of the HATH/PWWP domain of HDGF to cell surface receptors to modulate downstream signaling, as well as its ability to target the nucleus by its nuclear localization signals [71] suggested novel roles for the PWWP domains as integrators in global cellular signaling networks. These possibilities remain to be explored.

## Conclusions

Analysis of the distribution patterns of the versatile PWWP domain in proteins from ancestrally related genomes did not support models wherein complex protein architectures have appeared with the evolution of multicellularity and increased organismal complexity. Our analyses revealed that most of the high-level PWWP domain combinations in extant green plants’ or human genomes are present in their respective unicellular relatives. These data do not support models wherein the occurrence of complex architectures results by accretion of domains from simpler systems either. In fact, the simplest arrangements represented by proteins carrying a single PWWP domain are the least conserved and are found in both unicellular and multicellular genomes as species- or lineage-specific genes. Gain/loss of a PWWP domain at the termini of existing proteins is a recurring feature reflecting dynamic lineage- or species-specific events and not an increase in the degree of protein architecture correlated with increased organismal complexity. When present as a component of a multidomain arrangement the PWWP domain exists in both species- or lineage-specific combinations and in conserved cassettes inherited through the unicellular/multicellular evolution. These results indicate that multidomain combinations carrying the versatile PWWP domain are not volatile [17] but rather have survived as stable arrangements, apparently, driven by evolutionary descent and functional requirements. However, the PWWP domain in association with the VHS-ENTH-ANTH domains found in the land plants, but not algae, and of the PWWP domain in association with the GLYR1 function found in the human genome, but not in the cnidarian or the choanoflagellate, may represent novel occurrences in the plant or metazoan lineages, respectively. PWWP domains associated with DNA methyltransferases seen in some marine metagenomes and in the metazoan lineage provide plausible candidates for reinvented architectures resulting from convergent evolution.

## Methods

### PWWP-domain sequence search

PWWP-domain sequences were initially searched in *Arabidopsis thaliana* (the NCBI) database. Three search methods were used to mine new PWWP-domain proteins from all listed organisms: BLAST protein similarity searches were conducted by BLASTP [72] using the Arabidopsis PWWP-domain sequences as the queries against the non-redundant database available at NCBI with the default settings. To find similar protein regions from un-annotated genomic regions, TBLASTN [72] was used to perform similarity searches against nucleotide sequences of the genomes translated in all six frames. More sensitive searches were performed using the position specific iteration BLAST (PSI-BLAST) [73]. Each query was used against individual genomes with the inclusion E-value threshold of 0.001 and four search iterations. Furthermore, with the PWWP-containing proteins obtained at the NCBI, new BLAST searches were conducted to collect sequences from the Phytozome (http://www.phytozome.net/), as well as from the DOE Joint Genome Institute (http://www.jgi.doe.gov/). After performing the similarity searches, all non-redundant hits were compiled and each of these sequences was manually examined to confirm the presence of the PWWP domain by searching the Conserved Domain Database (CDD) available from NCBI [74] as well as the Simple Modular Architecture Research Tool (SMART) database [75,76]. In addition, the InterPro Scan Sequence Search web page (http://www.ebi.ac.uk/Tools/pfa/iprscan/) was used. All 14 applications, including TIGRFAM, Superfamily, Gene3D, Panther, PFam, were used to re-test and confirm the analyses. In general, there was a very good consistence between the various prediction models from these applications and those at NCBI.

### Multiple alignments of SET-domain sequences

The ClustalX software, version 2.0 [77] was employed to generate multiple sequence alignments of the PWWP-domain sequences obtained from the different databases (see Additional file 6). Once created, the ClustalX alignments were used to generate Neighbor Joining (NJ) phylogenetic trees with the MEGA4 program [78]. Multiple sequence alignment of proteins to generate the Maximum Likelihood phylogenetic trees was performed with the MUSCLE software [79].

### Phylogenetic analyses

Neighbor-Joining phylogeny of PWWP containing proteins was conducted with the MEGA4 program [78]. The evolutionary history was inferred using the Neighbor-Joining method [80]. The bootstrap consensus tree inferred from 5000 replicates was taken to represent the evolutionary history of the analyzed taxa [81]. Branches corresponding to partitions reproduced in less than 50% bootstrap replicates were collapsed. The percentage of replicate trees in which the associated taxa clustered together in the bootstrap test (5000 replicates) are shown next to the branches in all phylogenies. The trees were drawn to scale, with branch lengths in the same units as those of the evolutionary distances used to infer the phylogenetic tree. All positions containing gaps and missing data were eliminated from the datasets (Complete deletion option).

Maximum Likelihood phylogeny of PWWP containing proteins was performed with the “Phylogeny Pipeline” at http://phylogeny.lirmm.fr/[82]. Multiple sequence alignment of proteins was performed with the MUSCLE software [79], curation of the alignment was done with the GBLOCKS program [83], Maximum Likelihood trees with approximate Likelihood Ratio Test for branches (PhyML + aLRT) was performed with the PhyML3.0 program [84,85], and trees were drawn with TREEDYN software [86].

## Authors’ contributions

RAV performed sequence and phylogenetic analyses, as well as tree constructions; ZA conceived the study and analyzed the data. Both authors contributed to the writing and preparation of the manuscript.

## Supplementary Material

Additional file 1 Twelve genomes used in this study. Click here for file

Additional file 2 Phylogeny of the 12 taxa included in the study. Click here for file

Additional file 3 ** The genes encoding the PWWP domain containing proteins in *A. thaliana *and in *A. lyrata.*** Click here for file

Additional file 4 ** Maximum Likelihood phylogeny of PWWP containing proteins in *A. thaliana. *** Click here for file

Additional file 5 ** Neighbor-Joining phylogeny of PWWP containing proteins in *A. thaliana. *** Click here for file

Additional file 6 Multiple sequence alignments used for generating the phylogenetic trees in this study. Click here for file

Additional file 7 Gene architecture of the PWWP domain containing proteins from the 12 genomes studied. Click here for file

Additional file 8 ** Neighbor-Joining phylogeny of PWWP containing proteins in plants including the ATX3-like protein from*O. tauri *and the ATX1-like protein of Chlamydomonas.** Click here for file

Additional file 9 ** Neighbor-Joining phylogeny of PWWP containing proteins in humans, Nematostella,* M. brevicolis *and *O. tauri *.** Click here for file
