# Supplementary material for: Evolution of the PWWP-domain encoding genes in the plant and animal lineages
Source: BMC Evol Biol. 2012 Jun 26;12:101. doi: 10.1186/1471-2148-12-101 (PMC3457860; doi:10.1186/1471-2148-12-101)
Supplement: Additional file 1 — Twelve genomes used in this study. [file 1471-2148-12-101-S1.pdf]

Twelve genomes used in this study.

Species	Genome size / Gene number	Number of genes encoding PWWP containing proteins	Genome sequencing / Version number	Reference
*Arabidopsis thaliana (At)**	119.67 Mb / 33,583	16	Assembly Name TAIR9	http://www.ncbi.nlm.nih.gov/ genome/?term=Arabidopsis%20TAIR10
Arabidopsis lyrata (Al)	207.0 Mb / 32,670	14	US DOE Joint Genome Institute Program CSP 2006	http://genome.jgi-psf.org/programs/ plants/plant-projects.jsf
Sorghum bicolor (Sb)	698.2 Mb / 33,080	10	Assembly Name Sorbi1	http://www.ncbi.nlm.nih.gov/ genome?term=txid4558%5Borgn%5D
Populus trichocarpa (Pt)	427.47 Mb / 42,965	15	Assembly name Poptr1_1	http://www.ncbi.nlm.nih.gov/ genome?term=txid3694%5Borgn%5D
Physcomitrella patens (Pp)	480.0 Mb / 32,272	7	US DOE Joint Genome Institute Program CSP 2009	http://genome.jgi-psf.org/programs/ plants/plant-projects.jsf
Selaginella moellendorffii (Sm)	212.5 Mb / 22,273	10	US DOE Joint Genome Institute Program CSP 2005	http://genome.jgi-psf.org/programs/ plants/plant-projects.jsf
Chlamydomonas reinhardtii CC-503 (*Cr*)	105.19 Mb / 14,354	5	http://www.ncbi.nlm.nih.gov/ Taxonomy/Browser/wwwtax. cgi?id=3055	http://www.ncbi.nlm.nih.gov/Taxonomy/ Browser/wwwtax.cgi?id=3055
Volvox carteri f. Nagariensis (Vc)	120 Mb / 14,437	4	US DOE Joint Genome Institute Program DOEM 2005	http://genome.jgi-psf.org/programs/ plants/plant-projects.jsf

Ostreococcus tauri OTH95 RCC745-2009 (*Ot*)	12.56 Mb / 8,166	5	US DOE JGIP Program CSP 2010	http://genome.jgi-psf.org/programs/plants/plant-projects.jsf
Homo sapiens *(Hs)*	3095.69 Mb / 36,073	24	Human Genome Project - Assembly GRCh37.p7	http://www.ncbi.nlm.nih.gov/genome/guide/human/
Nematostella vectensis *(Nv)*	450 Mb / 18,000	6	US DOE Joint Genome Institute Program DOEM 2004	http://genome.jgi-psf.org/programs/plants/plant-projects.jsf
Monosiga brevicollis MX1 (*Mb*)	41.6 Mb / 9,200	4	US DOE Joint Genome Institute Program DOEM 2003	http://genome.jgi-psf.org/programs/plants/plant-projects.jsf

* abbreviations for the species shown in brackets
