# Supplementary material for: Evolution of the PWWP-domain encoding genes in the plant and animal lineages
Source: BMC Evol Biol. 2012 Jun 26;12:101. doi: 10.1186/1471-2148-12-101 (PMC3457860; doi:10.1186/1471-2148-12-101)
Supplement: Additional file 2 — Phylogeny of the 12 taxa included in the study. [file 1471-2148-12-101-S2.pdf]

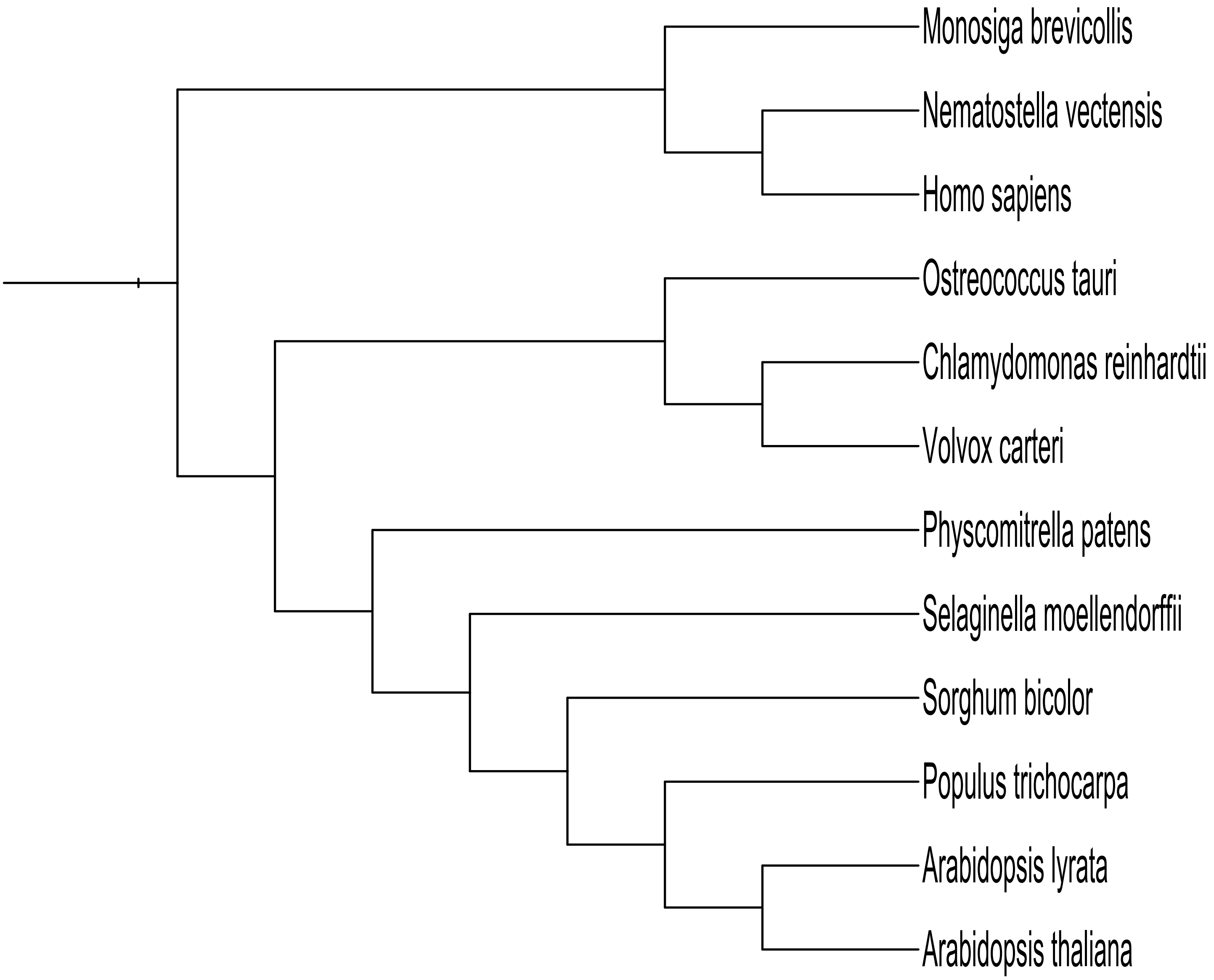

Additional File 2. Phylogeny of the 12 taxa included in the study. Tree was generated at the Interactive Tree Of Life online tool for the display and manipulation of phylogenetic trees (http://itol.embl.de/index.shtml). Phylogeny of the 12 taxa used in this study is represented as a trimmed version of NCBI taxonomy.
