# Supplementary material for: Evolution of the PWWP-domain encoding genes in the plant and animal lineages
Source: BMC Evol Biol. 2012 Jun 26;12:101. doi: 10.1186/1471-2148-12-101 (PMC3457860; doi:10.1186/1471-2148-12-101)
Supplement: Additional file 3 — The genes encoding the PWWP domain containing proteins in A. thaliana and in A. lyrata. [file 1471-2148-12-101-S3.pdf]

GenBank locus tag *A. thaliana*	Protein ID *A. thaliana*
AT3G05430	NP_187194
AT5G27650	NP_198117
AT5G40340	NP_198850
AT3G27860	NP_189424
AT3G09670	NP_187578
AT5G02950	NP_195915
AT3G48190	NP_190402
AT5G53430	NP_200155
AT4G27910	NP_194520
AT3G61740	NP_191733
AT2G31650	NP_850170
AT1G05830	NP_172074
AT3G63070	NP_191866
AT2G48160	NP_850485
AT5G23150	NP_197706
AT5G08230	NP_196440

GenBank locus tag *A. lyrata*	Protein ID *A. lyrata*
ARALYDRAFT_902198	XP_002881186
ARALYDRAFT_311665	XP_002889570
ARALYDRAFT_907732	XP_002878409
ARALYDRAFT_907712	XP_002878396
ARALYDRAFT_918421	XP_002864242
ARALYDRAFT_913734	XP_002869532
ARALYDRAFT_484576	XP_002875407
ARALYDRAFT_489171	XP_002872040
ARALYDRAFT_487657	XP_002873347
ARALYDRAFT_486811	XP_002876705
ARALYDRAFT_484006	XP_002880345
ARALYDRAFT_323420	EFH52141
ARALYDRAFT_324953	EFH47262
ARALYDRAFT_317405	XP_002882413

Additional File 3. The genome of *A. thaliana* was examined with the PWWP domain sequence of the ATX1 protein as a probe, and 16 genes were identified encoding PWWP-containing proteins. The fourteen genes encoding PWWP-containing proteins in *A. lyrata* were identified by BLAST analysis with the *A. thaliana* sequences.
