# Supplementary material for: Evolution of the PWWP-domain encoding genes in the plant and animal lineages
Source: BMC Evol Biol. 2012 Jun 26;12:101. doi: 10.1186/1471-2148-12-101 (PMC3457860; doi:10.1186/1471-2148-12-101)
Supplement: Additional file 4 — Maximum Likelihood phylogeny of PWWP containing proteins in A. thaliana. [file 1471-2148-12-101-S4.pdf]

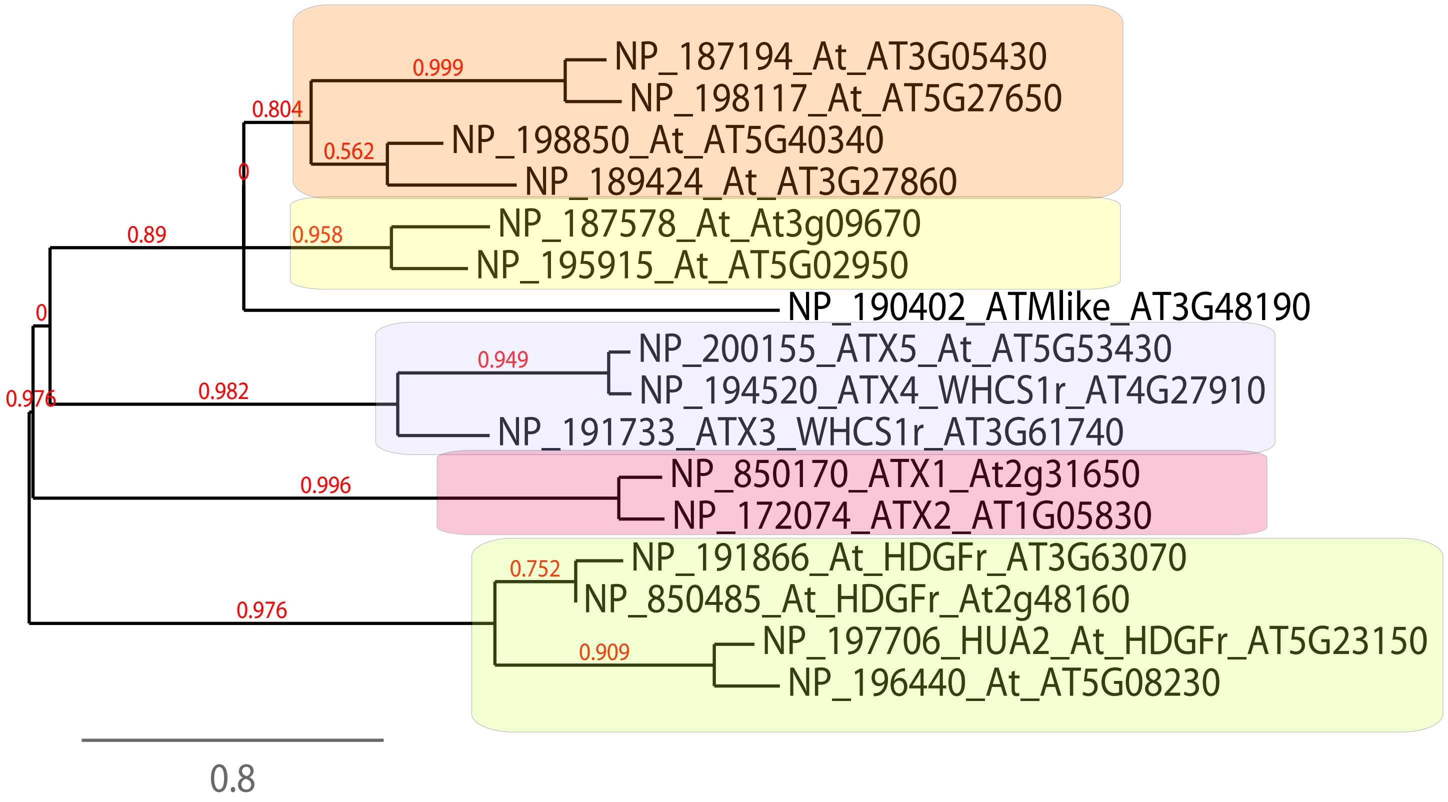

Additional File 4. Maximum Likelihood phylogeny of PWWP containing proteins in *A. thaliana*. The PWWP of ATX1 was used to perform database searches using BLAST at the NCBI network service (http://www.ncbi.nlm.nih.gov/blast). PWWP-domain sequences with the highest scores were used to generate the Maximum Likelihood phylogenetic tree. Species abbreviations correspond to *A. thaliana* (At). The branch length is proportional to the number of substitutions per site. Numbers in red correspond to the branch support values. The phylogenetic analysis was performed by using the Phylogeny Pipeline at http://phylogeny.lirmm.fr/. Subgroups containing the most closely related members are distinctly shaded.
