# Supplementary material for: Evolution of the PWWP-domain encoding genes in the plant and animal lineages
Source: BMC Evol Biol. 2012 Jun 26;12:101. doi: 10.1186/1471-2148-12-101 (PMC3457860; doi:10.1186/1471-2148-12-101)
Supplement: Additional file 6 — Multiple sequence alignments used for generating the phylogenetic trees in this study. [file 1471-2148-12-101-S6.pdf]

Additional file 6. Multiple sequence alignments used for generating the phylogenetic trees in this study.

Multiple sequence alignment of *Arabidopsis thaliana* and *Arabidopsis lyrata* PWWP-domain containing sequences. The multiple sequence alignment was used to reconstruct the maximum likelihood (Fig. 3) phylogeny.

```
NP_190402_ATMlike_At_AT3G48190      -----GNLVWVMTKY-KKWWPGEVVDF--KADAKES----
EFH52141_Alyrata                    -----GNLVWVMTKY-KKWWPGEVVDF--KADAKES----
NP_195915_At_AT5G02950              ------DLVWAKLRS-YPWWPGLVFDK--SVASKAAMRHF
EFH47262_Alyrata                    ------DLVWAKIRS-YPWWPGQVIDA--SVASKAAKKHF
BAF01554_At_At3g09670               --ISDSDLVWAKVRS-HPWWPGQVFDA--SAATDKAKKHF
NP_187194_At_AT3G05430              --FEVGDMVWGKVKS-HPWWPGQIFNE--AFASPSVRRVK
XP_002882413_Alyrata                --FEVGDMVWGKVKS-HPWWPGQIFNE--AFASPSVRRMK
NP_198117_At_AT5G27650              --FEVGDLVWGKVKS-HPWWPGHIFNE--AFASPSVRRMR
NP_189424_At_AT3G27860              --FHVGDFVWGEEANSQQWWPGQIYDS--LDASDLALKTM
XP_002875407_Alyrata                --FHVGDFVWGKEVNSQQWWPGQIYDS--LDASDLALKTM
NP_198850_At_AT5G40340              -----GDFVWGKIKN-HPWWPGQIYDP--SDASDLALKIK
XP_002878396_firstPWWP_Alyrata      -----------------PAWPAMVVDP--ISQAPDGVLKH
XP_002878396_secondPWWP_Alyrat      ------------------------------RSQAPTAVLKH
XP_002878409_Alyrata                --FTVGDLVWAKCGKRFPAWPAVVIDP--ISQAPDGVLKH
NP_191733_ATX3_WHCS1r_AT3G6174      --FTVGDLVWAKCGKRFPAWPAVVIDP--ISQAPDGVLKH
NP_194520_ATX4_WHCS1r_AT4G2791      --FYSGDLVWAKSGRNEPFWPAIVIDP--MTQAPELVLRS
XP_002869532_Alyrata                --FYSGDLVWAKSGRNEPFWPAIVIDP--MTQAPELVLRS
NP_200155_ATX5_At_AT5G53430         --FYSGDLVWGKSGRNEPFWPAIVIDP--MTQAPELVLRS
XP_002864242_Alyrata                --FYSGDLVWGKSGRKEPFWPAIVIDP--MTQAPELVLRS
NP_850170_ATX1_At2g31650            --FEPGDIVWAKLAG-HAMWPAVIVDESIIGERKGLNNKV
XP_002881186_Alyrata                --FETGDIVWAKLAG-HAMWPAVIVDESVIGERKGLNNKV
NP_172074_ATX2_AT1G05830            --FEPRDIIWAKLTG-HAMWPAIIVDESVIVKRKGLNNKI
XP_002889570_Alyrata                --FEPRDIIWAKLTG-HAMWPAIIVDESVIVKRKGLNNKA
NP_191866_At_HDGFr_AT3G63070        -EWKVGDLVLAKVKG-FPAWPAVVDEP------EKWG-HS
NP_850485_At_HDGFr_At2g48160        -----GDLVLAKVKG-FPAWPAVVSEP------EKWD-AS
XP_002880345_Alyrata                -KWKVGDLVLAKVKG-FPAWPAAVSEP------EKWG-AL
XP_002876705_Alyrata                ---KVGDLVLAKVKG-FPAWPAAVSQP------EKWG-YS
NP_197706_HUA2_At_HDGFr_AT5G23      -QLVLGDLVLAKVKG-FPAWPAKISRP------EDWD-RA
XP_002872040_Alyrata                GQLILGDLVLAKVKG-FPAWPAKISRP------EDWD-RA
NP_196440_At_AT5G08230              GEMRLGDLVLAKVKG-FPAWPAKIGQP------EDWN-QA
XP_002873347_Alyrata                --LRLGDLVLAKVKG-FPAWPAKIGQP------EDWN-QA


NP_190402_ATMlike_At_AT3G48190      -----FMVRSIG------QSHLVSWFASSKLKPFKESFEQ
EFH52141_Alyrata                    -----FMVRYIG------QSHLVSWFAPLKLKPFKESFEQ
NP_195915_At_AT5G02950              KK-GNVLVAYFG------DCTFA-WNNASQIKPFHQNFSQ
EFH47262_Alyrata                    KKKGNLLVAYFG------DCTFA-WNNASQVKPFHQNFSQ
BAF01554_At_At3g09670               KK-GSFLVTYFG------DCTFA-WNEASRIKPFRQHFSQ
NP_187194_At_AT3G05430              KM-GYVLVAFFG------DNSYG-WFDPAELIPFEPHVKE
XP_002882413_Alyrata                KM-GYVLVAFFG------DNSYG-WFDPAELLPFEPHVAE
NP_198117_At_AT5G27650              RI-DHVLVAFFG------DSSYG-WFDPAELIPFEPNLEE
NP_189424_At_AT3G27860              QK-GKLLVAYFG------DGSFFGWCNPLELKPFLENFKE
XP_002875407_Alyrata                QK-GKLLVAYFG------DGKFC-WCNPLELKPFLENFKE
NP_198850_At_AT5G40340              QK-GKLLVACFG------DGTFA-WCGASQLKPFAESFKE
XP_002878396_firstPWWP_Alyrata      CVPGAICVMFFGYSKNGTQRDYA-WVRQGMVYPFTEFMDK
```

```
XP_002878396_secondPWWP_Alyrat      CVPGAICVMFFGYLKNGTQSDYA-RVRQGMMYPFTEFMDK
XP_002878409_Alyrata                CVPGAICVMFFGYSKDGTQRDYA-WVRQGMMYPFTEFMDK
NP_191733_ATX3_WHCS1r_AT3G6174      CVPGAICVMFFGYSKDGTQRDYA-WVRQGMVYPFTEFMDK
NP_194520_ATX4_WHCS1r_AT4G2791      CIPDAACVVFFGHSGNENERDYA-WVRRGMIFPFVDYVAR
XP_002869532_Alyrata                CIPDAACVVFFGHSGNENERDYA-WVRRGMIFPFVDYVAR
NP_200155_ATX5_At_AT5G53430         CIPDAACVMFFGHSGTENERDYA-WVRRGMIFPFVDYVER
XP_002864242_Alyrata                CIPDAACVMFFGHSGTENERDYA-WVRRGMIFPFVDYVDR
NP_850170_ATX1_At2g31650            SGGGSLLVQFFG------THDFA-RIKVKQAISFIKGLLS
XP_002881186_Alyrata                SGGGSLLVQFFG------THDFA-RIKEKQAISFIKGLLS
NP_172074_ATX2_AT1G05830            SGGRSVLVQFFG------THDFA-RIQVKQAVSFLKGLLS
XP_002889570_Alyrata                SGGRSVLVQFFG------THDFA-R---------------
NP_191866_At_HDGFr_AT3G63070        ADSKKVTVHFFG------TQQIA-FCNHGDVESFTEEKKQ
NP_850485_At_HDGFr_At2g48160        PDSKKVFVHFFG------TQQIA-FCNPGDVEAFTEERKQ
XP_002880345_Alyrata                TDLKKVFVHFFG------TQQIA-FCNHTDVEAFTDERKQ
XP_002876705_Alyrata                ADSKKVLVHFFG------TQQIA-LCNPADVESFTEEKKQ
NP_197706_HUA2_At_HDGFr_AT5G23      PDPKKYFVQFFG------TEEIA-FVAPPDIQAFTSEAKS
XP_002872040_Alyrata                PDPKKYFVQFFG------TEEIA-FVAPPDIQAFTSEAKS
NP_196440_At_AT5G08230              PDPKKHFVQFYG------TGEIG-FVTPPDIQPFTSETKK
XP_002873347_Alyrata                PDPKKHFVQFFG------TQEIG-FVAPPDIQPFTSEAKN

NP_190402_ATMlike_At_AT3G48190      VLNQ-----RNDNGFFDALQKA-------
EFH52141_Alyrata                    VLNQ-----RNDKGFFDAVEKA-------
NP_195915_At_AT5G02950              MQEQ-----SNSAEFRDAIDCALDE----
EFH47262_Alyrata                    MQEQ-----SNLAEFRDAIDCALDE----
BAF01554_At_At3g09670               MAKQ-----SSLPDFIDAIDFALEE----
NP_187194_At_AT3G05430              KSQQ-----TSSDHFAKAVEEAMNE----
XP_002882413_Alyrata                NSQQ-----TSSGHFAKAVEEAMDE----
NP_198117_At_AT5G27650              KSQQQ----TVSKHFVRAVEEAKDE----
NP_189424_At_AT3G27860              FSKM-----SDSRRFLLAVEDA-------
XP_002875407_Alyrata                FSKM-----SESKRFLSAVEEA-------
NP_198850_At_AT5G40340              CSKV-----SNSRSFLGAVEEAVEE----
XP_002878396_firstPWWP_Alyrata      FQDKTNLYNYKPSEFKKALDEAVLAENG-
XP_002878396_secondPWWP_Alyrat      -----------------------------
XP_002878409_Alyrata                FQDQTNLYNYKPSEFKKALEEAVLAENG-
NP_191733_ATX3_WHCS1r_AT3G6174      FQDQTNLFNYKASEFNKALEEAVLAENGN
NP_194520_ATX4_WHCS1r_AT4G2791      FQEQPELQGCKPGNFQMALEEAFLADQGF
XP_002869532_Alyrata                FQEQSELQGCKPGNFQMALEEAFLADQGF
NP_200155_ATX5_At_AT5G53430         LQEQSELRGCNPRDFQMALEEALLADQGF
XP_002864242_Alyrata                FQEQSELRGCNPREFQMALEEALLADQGF
NP_850170_ATX1_At2g31650            PSHLK-CKQ--PR-FEEGMQEA-------
XP_002881186_Alyrata                PSHLK-CKQ--PR-FEEGMQEA-------
NP_172074_ATX2_AT1G05830            RSPLK-CKQ--PR-FEEAMEEA-------
XP_002889570_Alyrata                -----------------------------
NP_191866_At_HDGFr_AT3G63070        SLLTR-RHAKGSD-FVRAVKEITES----
NP_850485_At_HDGFr_At2g48160        SLLTR-RHAKGSD-FVRAVKEIIES----
XP_002880345_Alyrata                SLLTK-RHAKGSD-FLRAVKEIIES----
XP_002876705_Alyrata                LLLTK-RHAKGSD-FVRAVKEITES----
NP_197706_HUA2_At_HDGFr_AT5G23      KLLAR-CQGKTVKYFAQAVEQICTA----
XP_002872040_Alyrata                KLLAR-CQGKTVKYFAQAVEQICTA----
NP_196440_At_AT5G08230              KLSAR-CQGKTVKYFSQAVEEI-------
XP_002873347_Alyrata                KLLAR-CQGKTVKYFSQAVEEI-------
```

Multiple sequence alignment of plant PWWP-domain containing sequences. The multiple sequence alignment was used to reconstruct the neighbor joining (Fig. 4) phylogeny.

```
NP_850170_ATX1_At2g31650            ----------------FEPGDIVWAKLAG--HAMWPAVIV
XP_002881186_Alyrata                ----------------FETGDIVWAKLAG--HAMWPAVIV
NP_172074_ATX2_AT1G05830            ----------------FEPRDIIWAKLTG--HAMWPAIIV
XP_002301643_Populus                ----------------LEPGDIIWAKLTG--HAMWPAIVV
XP_002320433_Populus                ------------------PGDIIWAKVTG--HAMWPAIVV
XP_002889570_Alyrata                ----------------FEPRDIIWAKLTG--HAMWPAIIV
XP_002459927_Sorghum                ------------------PGDLVWAKITG--HAMWPAVVV
XP_002992195_Selaginella            -------------------GTVVWAKVKG--WPMWPALVL
XP_002982095_Selaginella            DELALLANEMESFEEQISHGTVVWAKVKG--WPMWPALVL
XP_001767466_Physcomitrella         -------------------GDLVWAKVKG--WPMWPAFVM
XP_001780587_Physcomitrella         -------------------GDLVWAKVKG--WPMWPAFVM
XP_002975241_Selaginella            ----------------FQHGDIVWAETPG--YPMWPAFVM
XP_001760849_Physcomitrella         ----------------FKHGQLVWAKFAR--FPWWPAEII
XP_001751216_Physcomitrella         -------------------GQLVWAKFAR--FPWWPAEII
XP_002961091_Selaginella            -----------------SAGQLVWAKFSR--SPWWPAQVA
XP_002966918_Selaginella            -----------------SAGQLVWAKFSR--SPWWPAQVA
XP_002444527_Sorghum                ----------------FRLGDITWVKCSG--SSWWPAQVI
NP_189424_At_AT3G27860              ----------------FHVGDFVWGEEANS-QQWWPGQIY
XP_002875407_Alyrata                ----------------FHVGDFVWGKEVNS-QQWWPGQIY
NP_198850_At_AT5G40340              -------------------GDFVWGKIKN--HPWWPGQIY
XP_002305994_Populus                ----------------FRVGDFVWGKIKS--HPWWPGRVY
NP_195915_At_AT5G02950              --------------------DLVWAKLRS--YPWWPGLVF
XP_002871003_Alyrata                --------------------DLVWAKIRS--YPWWPGQVI
NP_187578_At_At3g09670              ----------------ISDSDLVWAKVRS--HPWWPGQVF
XP_002882633_Alyrata                ----------------ISDSDLVWAKVRS--HPWWPGQVF
XP_002326168_Populus                ----------------LSVGDLVWGKVRS--HPWWPGQVF
XP_002322856_Populus                -------------------GDLVWGKVRS--HPWWPGQLF
XP_002315275_Populus                ----------------FSVSDLVWGKVRS--HPWWPGQIF
XP_002439929_Sorghum                --------------------DLVWGKVKS--HPWWPGEIF
XP_002440507_Sorghum                ------------------PGRLVWGKVRD--HPWWPAQVF
XP_002318650_Populus                ----------------FHVGDIVWVKTKN--QSWWPGKIF
XP_002461281_Sorghum                ----------------FAPGDMVWGKKLN--HAAWPGLIY
NP_187194_At_AT3G05430              ----------------FEVGDMVWGKVKS--HPWWPGQIF
XP_002882413_Alyrata                ----------------FEVGDMVWGKVKS--HPWWPGQIF
XP_002319529_Populus                ----------------FEVGDMVWGKVKS--HPRWPGHIF
XP_002330150_Populus                ----------------FEVGDMVWGKVKS--HPWWPGHIF
NP_198117_At_AT5G27650              ----------------FEVGDLVWGKVKS--HPWWPGHIF
XP_002330341_Populus                ----------------------------------------
XP_002439285_Sorghum                ----------------PRFGDMVWAKVKS--HPWWPGHIY
NP_191866_At_HDGFr_AT3G63070        ---------------EWKVGDLVLAKVKG--FPAWPAVVD
NP_850485_At_HDGFr_At2g48160        -------------------GDLVLAKVKG--FPAWPAVVS
XP_002880345_Alyrata                ---------------KWKVGDLVLAKVKG--FPAWPAAVS
XP_002876705_Alyrata                -----------------KVGDLVLAKVKG--FPAWPAAVS
XP_002465241_Sorghum                ----------------WKVGDLVLAKMKG--FPAWPAMIS
XP_002463300_Sorghum                ---------------QWKVGDLVLAKMKG--FPAWPAMQ-
XP_002974023_Selaginella            ---------------QWKIGDLVLAKVKG--FPAWPAQVS
XP_002983468_Selaginella            ---------------QWKIGDLVLAKVKG--FPAWPAQVS
XP_002962776_Selaginella            ---------------EWSVGDLVLAKVKG--FPAWPAQVS
NP_197706_HUA2_At_HDGFr_AT5G23      ---------------QLVLGDLVLAKVKG--FPAWPAKIS
XP_002872040_Alyrata                --------------GQLILGDLVLAKVKG--FPAWPAKIS
NP_196440_At_AT5G08230              --------------GEMRLGDLVLAKVKG--FPAWPAKIG
XP_002873347_Alyrata                ----------------LRLGDLVLAKVKG--FPAWPAKIG
```

```
XP_002310078_Populus             ---------------QLRLGDLVLAKVKG--YPSWPAKIS
XP_002306384_Populus             ---------------QLRLGDLVLAKVKG--YPSWPAKIS
XP_002443678_Sorghum             --------------REPQLGDLVLAKIKG--YPPWPAKIS
XP_001753837_Physcomitrella      --------------REFNEGDLVLAKVKG--WPAWPAQAE
XP_001765392_Physcomitrella      --------------REFNVGDLVLAKVKG--WPAWPAQ--
XP_002878409_Alyrata             ----------------FTVGDLVWAKCGKR-FPAWPAVVI
XP_002878396_Alyrata             --------------------------------PAWPAMVV
NP_191733_ATX3_WHCS1r_AT3G6174   ----------------FTVGDLVWAKCGKR-FPAWPAVVI
XP_002302629_Populus             ----------------FALGDIVWAKCGKR-YPWWPAIVI
NP_194520_ATX4_WHCS1r_AT4G2791   ----------------FYSGDLVWAKSGRN-EPFWPAIVI
XP_002869532_Alyrata             ----------------FYSGDLVWAKSGRN-EPFWPAIVI
NP_200155_ATX5_At_AT5G53430      ----------------FYSGDLVWGKSGRN-EPFWPAIVI
XP_002864242_Alyrata             ----------------FYSGDLVWGKSGRK-EPFWPAIVI
XP_002318412_Populus             ----------------FYSGDLVWAKSGMK-YPFWPAIVI
XP_002321418_Populus             ----------------FYSGDIVWAKSGNK-YPFWPAIVI
XP_002454931_Sorghum             ----------------FVLGDVVWARSGKK-SPTWPALVI
XP_002961390_Selaginella         ----------------FDIGDIVWAKSGKKKDPVWPAKVV
XP_002980880_Selaginella         ----------------FDIGDIVWAKSGKKKDPVWPAKVV
XP_001777592_Physcomitrella      ----------------FDLGEIVWAKSGKRNDPVWPARVI
XP_002456070_Sorghum             ----------------LVYGDIVWAKLGKR-QPMWPGVLI


NP_850170_ATX1_At2g31650         DESIIGERKGLNNKVSGGGSLLVQFFGTH-DFARI-----
XP_002881186_Alyrata             DESVIGERKGLNNKVSGGGSLLVQFFGTH-DFARI-----
NP_172074_ATX2_AT1G05830         DESVIVKRKGLNNKISGGRSVLVQFFGTH-DFARI-----
XP_002301643_Populus             DGALIGDHKGISKNI-GGGSISVQFFGTH-DFARI-----
XP_002320433_Populus             DEALIGNHKGLSKNI-GGRSVSVQFFGTH-DFARY-----
XP_002889570_Alyrata             DESVIVKRKGLNNKASGGRSVLVQFFGTH-DFAR------
XP_002459927_Sorghum             DESNVPATRAL-KSIRLDQSILVQFFGTH-DFARF-----
XP_002992195_Selaginella         DEE-HAEKCGLERPLKKST-FAVQFFGSC-DFARL-----
XP_002982095_Selaginella         DEE-HAEKCGLERPLKKST-FAVQFFGSC-DFARL-----
XP_001767466_Physcomitrella      DED-HAAACGME-PGKKGM-VPLQFFGSY-DHCRF-----
XP_001780587_Physcomitrella      DED-HAAACGMD-PGKKGM-VPLQFFGSY-DHCRF-----
XP_002975241_Selaginella         DDQ-HARLCNLE--ISTGE-LPVHYFGTY-ESA-------
XP_001760849_Physcomitrella      NERAFQPGTGGN--RRSNTDVLVRFFGTY-DYG-------
XP_001751216_Physcomitrella      NEKAVIPGTGGN--KRINADVLSKCF-IY-GLGSDGKCSG
XP_002961091_Selaginella         DEKSMSERT-----RQSKNHVLVRFFGNY-NYG-------
XP_002966918_Selaginella         DEKSMSERT-----RQSKNHVLVRFFGNY-NYG-------
XP_002444527_Sorghum             DELCVGSKP--K--KKDKYDCLVRLYGTC-QYL-------
NP_189424_At_AT3G27860           DSL--DASDLALKTMQK-GKLLVAYFGDG-SFFG------
XP_002875407_Alyrata             DSL--DASDLALKTMQK-GKLLVAYFGDG-KFC-------
NP_198850_At_AT5G40340           DPS--DASDLALKIKQK-GKLLVACFGDG-TFA-------
XP_002305994_Populus             DPS--NASDYAKKVKQR-DKILVAYFGDS-TFA-------
NP_195915_At_AT5G02950           DKS--VASKAAMRHFKK-GNVLVAYFGDC-TFA-------
XP_002871003_Alyrata             DAS--VASKAAKKHFKKKGNLLVAYFGDC-TFA-------
NP_187578_At_At3g09670           DAS--AATDKAKKHFKK-GSFLVTYFGDC-TFA-------
XP_002882633_Alyrata             DAS--AATDKAKKYFKK-GSFLVTYFGDC-TFA-------
XP_002326168_Populus             GRS--DASKKAKKYFKK-NSYLIAYFGDQ-TFA-------
XP_002322856_Populus             GCA--DASKKAKKHFKK-DSYLIAYLGDQ-TFA-------
XP_002315275_Populus             DPS--DASEKAMRYHKK-DCYLVAYFGDR-TFA-------
XP_002439929_Sorghum             DPS--DASELALKHQKK-GSHLVAYFGDN-TFA-------
XP_002440507_Sorghum             DAA--DASADARALRRPRGAVLVAYFWDK-TFA-------
XP_002318650_Populus             DPL--GVTKYAVQSDQR-NGLLVGYLGSC-HIA-------
XP_002461281_Sorghum             SAG--GNGTGHE------GQFLVSYFGDK-AFA-------
NP_187194_At_AT3G05430           NEA--FAS-PSVRRVKKMGYVLVAFFGDN-SYG-------
XP_002882413_Alyrata             NEA--FAS-PSVRRMKKMGYVLVAFFGDN-SYG-------
XP_002319529_Populus             NEA--FAS-SSVRRTRREGHVLVAFFGDS-SYG-------
```

```
XP_002330150_Populus              NEA--FAS-SSVRRTRREGHVLVAFFGDS-SYG-------
NP_198117_At_AT5G27650            NEA--FAS-PSVRRMRRIDHVLVAFFGDS-SYG-------
XP_002330341_Populus              ----------------REGHVLVAFFGDG-SYG-------
XP_002439285_Sorghum              SVN--LTDDEEVHRGYRDGLVLVAFFGDS-SYG-------
NP_191866_At_HDGFr_AT3G63070      -------EPEKWGHSADSKKVTVHFFGT--QQIA------
NP_850485_At_HDGFr_At2g48160      -------EPEKWDASPDSKKVFVHFFGT--QQIA------
XP_002880345_Alyrata              -------EPEKWGALTDLKKVFVHFFGT--QQIA------
XP_002876705_Alyrata              -------QPEKWGYSADSKKVLVHFFGT--QQIA------
XP_002465241_Sorghum              -------EPEQWKMPSTKKKPLVYFYGT--KQIA------
XP_002463300_Sorghum              ---------------FIKYSVHINIYRS--QDIV------
XP_002974023_Selaginella          -------RAEDFGRQRNPAKVFVVFFGT--KQIR------
XP_002983468_Selaginella          -------RAEDFGRQRNPAKVFVVFFGT--KQIR------
XP_002962776_Selaginella          -------KPEAFGQHHNPARVFVVFFGT--KQIR------
NP_197706_HUA2_At_HDGFr_AT5G23    -------RPEDWDRAPDPKKYFVQFFGT--EEIA------
XP_002872040_Alyrata              -------RPEDWDRAPDPKKYFVQFFGT--EEIA------
NP_196440_At_AT5G08230            -------QPEDWNQAPDPKKHFVQFYGT--GEIG------
XP_002873347_Alyrata              -------QPEDWNQAPDPKKHFVQFFGT--QEIG------
XP_002310078_Populus              -------RPEDWKRVADAKKVFVYFFGT--QEIA------
XP_002306384_Populus              -------RPEDWKRAPDAKKVFVYFFGT--QEIA------
XP_002443678_Sorghum              -------RPEDWQHKPTPKKFFVYFYGT--EEIA------
XP_001753837_Physcomitrella       VSCFPQEIGKLETQGKHAGKYYVTYFGPG-QQIG------
XP_001765392_Physcomitrella       -------IGRLETQGKHAGKYYVTYFGPG-QQIG------
XP_002878409_Alyrata              DPISQAPDGVL--KHCVPGAICVMFFGYS-KDG-TQRDYA
XP_002878396_Alyrata              DPISQAPDGVL--KHCVPGAICVMFFGYS-KNG-TQRDYA
NP_191733_ATX3_WHCS1r_AT3G6174    DPISQAPDGVL--KHCVPGAICVMFFGYS-KDG-TQRDYA
XP_002302629_Populus              DPILQAPDAVL--SCCVPGAICIMFYGYS-KNG-TQRDYA
NP_194520_ATX4_WHCS1r_AT4G2791    DPMTQAPELVL--RSCIPDAACVVFFGHS-GNE-NERDYA
XP_002869532_Alyrata              DPMTQAPELVL--RSCIPDAACVVFFGHS-GNE-NERDYA
NP_200155_ATX5_At_AT5G53430       DPMTQAPELVL--RSCIPDAACVMFFGHS-GTE-NERDYA
XP_002864242_Alyrata              DPMTQAPELVL--RSCIPDAACVMFFGHS-GTE-NERDYA
XP_002318412_Populus              DPMTQAPELVL--RSCIADAACVMFFGCS-GNDGDQRDYA
XP_002321418_Populus              DPMTQAPELVL--RSCIADAACVMFFGCS-GNDGNQRDYA
XP_002454931_Sorghum              DPMQHAPEVVL--NSCVPGALCVMFFGYS-ANG-HGRDYG
XP_002961390_Selaginella          DPIKEVPESVR--RKCVPNRLCVMFYGESLARGVKRRDYA
XP_002980880_Selaginella          DPIKEVPESVR--RKCVPNRLCVMFYGESLARGVKRRDYA
XP_001777592_Physcomitrella       DPFREAPPMVR--ELSLPNSLCVMFYGPSSSKGKHSRDYA
XP_002456070_Sorghum              DPTQQVAADAMPPQPRRVAVLCVMLFGWCTEFS-DEKKYV


NP_850170_ATX1_At2g31650          KVKQAISFIK-GLLSPSHLKCKQP-----------RFEEG
XP_002881186_Alyrata              KEKQAISFIK-GLLSPSHLKCKQP-----------RFEEG
NP_172074_ATX2_AT1G05830          QVKQAVSFLK-GLLSRSPLKCKQP-----------RFEEA
XP_002301643_Populus              KPKQAISFLK-GLLSSFHLKCKQP-----------RFTRS
XP_002320433_Populus              LSEQKLP---------------------------------
XP_002889570_Alyrata              --EDCHDFEP-RDIIWAKLTGHA-----------------
XP_002459927_Sorghum              ----------------------------------------
XP_002992195_Selaginella          NNDKIVTFSK-GVQLKYHSKCKRP-----------AFDQG
XP_002982095_Selaginella          NNDKIVTFSK-GVQLKYHSKCKRP-----------AFDQG
XP_001767466_Physcomitrella       SYKKLVIFSK-GLMMKFHTKCKRV-----------VFVQG
XP_001780587_Physcomitrella       SYKKLVIFSK-GLMMKFHTKCKRV-----------VFVQG
XP_002975241_Selaginella          ----------------------------------------
XP_001760849_Physcomitrella       WVDPEVGLSEFDVKMQERSRIKKK-----------AFQKG
XP_001751216_Physcomitrella       WVDPKVGLSEFDVKLQERSKIKKK-----------ALQKG
XP_002961091_Selaginella          WVDPASDLSKFDVDFDGRSQVPTK-----------AFQKG
XP_002966918_Selaginella          WVDPASDLSKFDVDFDGRSQVPTK-----------AFQKG
XP_002444527_Sorghum              YVDPWKSNSEFEMMLKQENKSTME-----------AFREV
NP_189424_At_AT3G27860            WCNPLE-LKPFLENFKEFSKMSDS----------RRFLLA
```

XP_002875407_Alyrata	WCNPLE-LKPFLENFKEFSKMSES----------KRFLSA
NP_198850_At_AT5G40340	WCGASQ-LKPFAESFKECSKVSNS----------RSFLGA
XP_002305994_Populus	WCNPSQ-LSPFEENFVEMFKQSNS----------KSFVNA
NP_195915_At_AT5G02950	WNNASQ-IKPFHQNFSQMQEQSNS----------AEFRDA
XP_002871003_Alyrata	WNNASQ-VKPFHQNFSQMQEQSNL----------AEFRDA
NP_187578_At_At3g09670	WNEASR-IKPFRQHFSQMAKQSSL----------PDFIDA
XP_002882633_Alyrata	WNDASR-VKPFRQHFSQMAKQSSL----------PDFIDA
XP_002326168_Populus	WNEVSK-IKPFRCNFSLLEKQSNL----------EDFHDA
XP_002322856_Populus	WNEVSK-IKPFRCNFSLLEKQSNL----------EDFHDA
XP_002315275_Populus	WNEASL-LKPFRSHFSQVEKQSNS----------EVFQNA
XP_002439929_Sorghum	WCDESQ-LKPFVTNYSQMEKQSTS----------DAFVGS
XP_002440507_Sorghum	WNDAAA-LLPFRAGF------------------------
XP_002318650_Populus	WCLPSQ-LKPFHKDFEQMVVKNKA----------RSFLGA
XP_002461281_Sorghum	WCDGAE-LRPYEPYFPVAELYDDGG---------EDFDAA
NP_187194_At_AT3G05430	WFDPAE-LIPFEPHVKEKSQQ-TSS---------DHFAKA
XP_002882413_Alyrata	WFDPAE-LLPFEPHVAENSQQ-TSS---------GHFAKA
XP_002319529_Populus	WFDPAE-LIQFDVNFAEKSQQ-TNS---------RTFIKA
XP_002330150_Populus	WFDPAE-LIPFDANFAEKSQQ-TNS---------RTFIRA
NP_198117_At_AT5G27650	WFDPAE-LIPFEPNLEEKSQQQTVS---------KHFVRA
XP_002330341_Populus	WLDPTE-LVQFDSHYVEKSKQTNAK---------VFLIKA
XP_002439285_Sorghum	WFEPSE-LVPFEDHFTEK---------------------
NP_191866_At_HDGFr_AT3G63070	FCNHGD-VESFTEEKKQSLLTRRH-----AKGS--DFVRA
NP_850485_At_HDGFr_At2g48160	FCNPGD-VEAFTEERKQSLLTRRH-----AKGS--DFVRA
XP_002880345_Alyrata	FCNHTD-VEAFTDERKQSLLTKRH-----AKGS--DFLRA
XP_002876705_Alyrata	LCNPAD-VESFTEEKKQLLLTKRH-----AKGS--DFVRA
XP_002465241_Sorghum	FCNYAD-LEAFTEEKKRSLLAKRH-----GKGA--DFLRA
XP_002463300_Sorghum	EITEPE-QWDLPAAKKKSIVAKK-------KLL--VYFYG
XP_002974023_Selaginella	FCQHSE-ISAFTVEARAALHARTL-----SKCVPSDLKRA
XP_002983468_Selaginella	FCQHSE-ISAFTVEARAALHARTL-----SKCVPSDLKRA
XP_002962776_Selaginella	FCHHSE-ISKFTPEAKASLVAKAH-----SKCTPADLRLA
NP_197706_HUA2_At_HDGFr_AT5G23	FVAPPD-IQAFTSEAKSKLLARC-----QGKTV-KYFAQA
XP_002872040_Alyrata	FVAPPD-IQAFTSEAKSKLLARC-----QGKTV-KYFAQA
NP_196440_At_AT5G08230	FVTPPD-IQPFTSETKKKLSARC-----QGKTV-KYFSQA
XP_002873347_Alyrata	FVAPPD-IQPFTSEAKNKLLARC-----QGKTV-KYFSQA
XP_002310078_Populus	FVAPSD-IQVFTNEVKNKLSARC-----QSKKD-RFFSQA
XP_002306384_Populus	FVAPSD-IQVFTNEVKNKLSARC-----QSKKD-KFFSQA
XP_002443678_Sorghum	FVPLAD-LEEFTEKTKNDLLDRAPNIKVQRKYV-QVFNDA
XP_001753837_Physcomitrella	WCLPVE-LSEFDADQREASIQKSL-----KKTADKKFISA
XP_001765392_Physcomitrella	WCLPVE-LSEFDADQREASVQKSL-----KKTADKKFIFA
XP_002878409_Alyrata	WVRQGM-MYPFTEFMDKFQDQTNLYN-----YKPSEFKKA
XP_002878396_Alyrata	WVRQGM-VYPFTEFMDKFQDKTNLYN-----YKPSEFKKA
NP_191733_ATX3_WHCS1r_AT3G6174	WVRQGM-VYPFTEFMDKFQDQTNLFN-----YKASEFNKA
XP_002302629_Populus	WVKQGM-VFPFAEFMERFQVQSQMFK-----CKLSDFQVA
NP_194520_ATX4_WHCS1r_AT4G2791	WVRRGM-IFPFVDYVARFQEQPELQG-----CKPGNFQMA
XP_002869532_Alyrata	WVRRGM-IFPFVDYVARFQEQSELQG-----CKPGNFQMA
NP_200155_ATX5_At_AT5G53430	WVRRGM-IFPFVDYVERLQEQSELRG-----CNPRDFQMA
XP_002864242_Alyrata	WVRRGM-IFPFVDYVDRFQEQSELRG-----CNPREFQMA
XP_002318412_Populus	WVQRGM-IFPFLDFVDRFQEQSELDD-----CKPGDFQMA
XP_002321418_Populus	WVQRGM-IFPFMDFLDRFQEQSELDD-----FN-GDFQMA
XP_002454931_Sorghum	WVKQGM-IFPFVDYLDRFQGQP-LYK-----LRPSKFRAA
XP_002961390_Selaginella	WVRKGM-IFPFVDYLERFQSQTSFNK-----SQPGDFWSA
XP_002980880_Selaginella	WVRKGM-IFPFVDYLERFQSQTSFNK-----SQPGDFWSA
XP_001777592_Physcomitrella	WVKQGM-IFPFNAYLERFQSQTQLNR-----SRPVDFRQA
XP_002456070_Sorghum	WVRQGL-IFPFSDYMDRFQGQTELSS-----CKPADFRRA

NP_850170_ATX1_At2g31650	MQEA-------

```
XP_002881186_Alyrata               MQEA-------
NP_172074_ATX2_AT1G05830           MEEA-------
XP_002301643_Populus               LEEA-------
XP_002320433_Populus               -----------
XP_002889570_Alyrata               -----------
XP_002459927_Sorghum               -----------
XP_002992195_Selaginella           LRE--------
XP_002982095_Selaginella           LRE--------
XP_001767466_Physcomitrella        LEEVE------
XP_001780587_Physcomitrella        LEEVE------
XP_002975241_Selaginella           -----------
XP_001760849_Physcomitrella        IEEALEY----
XP_001751216_Physcomitrella        IEEALEF----
XP_002961091_Selaginella           LKEALEY----
XP_002966918_Selaginella           LKEALEY----
XP_002444527_Sorghum               LEK--------
NP_189424_At_AT3G27860             VEDA-------
XP_002875407_Alyrata               VEEA-------
NP_198850_At_AT5G40340             VEEAVEE----
XP_002305994_Populus               VKEAVDE----
NP_195915_At_AT5G02950             IDCALDE----
XP_002871003_Alyrata               IDCALDE----
NP_187578_At_At3g09670             IDFALEE----
XP_002882633_Alyrata               IDFALEE----
XP_002326168_Populus               VHCALDE----
XP_002322856_Populus               VHCALDE----
XP_002315275_Populus               VDCSLEE----
XP_002439929_Sorghum               VNNALEE----
XP_002440507_Sorghum               -----------
XP_002318650_Populus               VEKAVDE----
XP_002461281_Sorghum               VEASLDE----
NP_187194_At_AT3G05430             VEEAMNE----
XP_002882413_Alyrata               VEEAMDE----
XP_002319529_Populus               VEEATDE----
XP_002330150_Populus               VEEATDE----
NP_198117_At_AT5G27650             VEEAKDE----
XP_002330341_Populus               VEEADD-----
XP_002439285_Sorghum               -----------
NP_191866_At_HDGFr_AT3G63070       VKEITES----
NP_850485_At_HDGFr_At2g48160       VKEIIES----
XP_002880345_Alyrata               VKEIIES----
XP_002876705_Alyrata               VKEITES----
XP_002465241_Sorghum               VDEIVE-----
XP_002463300_Sorghum               TKE--------
XP_002974023_Selaginella           VKEICE-----
XP_002983468_Selaginella           VKEICE-----
XP_002962776_Selaginella           VSEIC------
NP_197706_HUA2_At_HDGFr_AT5G23     VEQICTA----
XP_002872040_Alyrata               VEQICTA----
NP_196440_At_AT5G08230             VEEI-------
XP_002873347_Alyrata               VEEI-------
XP_002310078_Populus               VKEICAA----
XP_002306384_Populus               VKEICAA----
XP_002443678_Sorghum               VEQICKA----
XP_001753837_Physcomitrella        VKEIC------
XP_001765392_Physcomitrella        VKEIC------
XP_002878409_Alyrata               LEEAVLAENG-
```

XP_002878396_Alyrata	LDEAVLAENG-
NP_191733_ATX3_WHCS1r_AT3G6174	LEEAVLAENGN
XP_002302629_Populus	LEEAILAESGF
NP_194520_ATX4_WHCS1r_AT4G2791	LEEAFLADQGF
XP_002869532_Alyrata	LEEAFLADQGF
NP_200155_ATX5_At_AT5G53430	LEEALLADQGF
XP_002864242_Alyrata	LEEALLADQGF
XP_002318412_Populus	VEEAFLAEQGF
XP_002321418_Populus	FEEAFLAEQGF
XP_002454931_Sorghum	IEEAFLAERGF
XP_002961390_Selaginella	IEEATLAEAG-
XP_002980880_Selaginella	IEEATLAEAG-
XP_001777592_Physcomitrella	ISEAKLAD---
XP_002456070_Sorghum	VEEAFLADQGF

Multiple sequence alignment of animal PWWP-domain containing sequences. The multiple sequence alignment was used to reconstruct the neighbor joining (Fig. 6) phylogeny.

```
3068_HDGF_NP_004485                 ----KEYKCGDLVFAKMKGYPHWPARIDE---MPEAAVKS
50810_HDGFRP3_CCDS32314             ----REYKAGDLVFAKMKGYPHWPARIDE---LPEGAVKP
84717_HDGFRP2_UNQ785PRO1604         ----HAFKPGDLVFAKMKGYPHWPARIDD---IADGAVKP
154150_HDGFL1_NP_612641             ------YKSGDLVFAKLKGYAHWPARIEH---MTQ-----
XP_001634069_Nv                     ----MTYQPGDLIWAKMRGYPHWPARIDL---AAKDEVIP
84656_GLYR1_NM_032569               ------LRLGDLVWGKLGRYPPWPGKIVN---PPKDLKKP
7468_a_WHSC1_NM_133330_WHSCr        ------LHFQDIIWVKLGNYRWWPAEVCHPKNVPPNIQKM
64324_a_NSD1_NM_172349_WHSCr        ------PHYREIVWVKVGRYRWWPAEICHPRAVPSNIDKM
54904_a_WHSC1L1_NM_023034_WHSC      ------LHYKQIVWVKLGNYRWWPAEICNPRSVPLNIQGL
XP_001635219_Nv                     ------LLYGDIVWVKLGMYRWWPAMICNPRDVPTNIQSM
114825_PWWP2Aisob_NP_001124336      ------ICVGDIVWAKIYGFPWWPARILT--ITVSRKDNG
170394_PWWP2B_NM_138499             ------VAVGDIVWGKIHGFPWWPARVLD--ISLGQKEDG
XP_001624451_Nv                     ------INEGSIIWGKVHGHPWWPGRVMA--ITEVVDDQG
1788_DNMT3A_NM_022552               ------FGIGELVWGKLRGFSWWPGRIVS--WWMTGRSRA
1789_DNMT3B_NM_006892               ------FGIGDLVWGKIKGFSWWPAMVVS--WKATSKRQA
XP_001626231_Nv                     ---------GVLVWSKLKGYDWWPGRVVT--YMEAGRPPP
54904_b_WHSC1L1_NM_023034_MSH6      -----KFQVGDLVWSKVGTYPWWPCMVSSDPQLEVHTKIN
7468_b_WHSC1_NM_133330_MSH6l        ----LKYNVGDLVWSKVSGYPWWPCMVSADPLLHSYTKLK
64324_b_NSD1_NM_172349_MSH6l        ----LKYEVGDLIWAKFKRRPWWPCRICSDPLINTHSKMK
152098_ZCWPW2_NP_001035522          -------PLGSLVLVKLQNWPSWPGILCPDRFKGKYVTYD
XP_001633052_Nv                     ----SPFPTGAIVWAKLAGYPWWPAMMEDDPDYGYYYETD
10771_ZMYND11_NM_006624             WFCYPCIPNHELVWAKMKGFGFWPAKVMQ----KEDNQVD
XP_001625954_Nv                     WFCKPCFFPHELVWAKMTGEPPWPAKLLT----WSDDQTR
23613_ZMYND8_NM_183047              WFCEPCSNPHPLVWAKLKGFPFWPAKALR----DKDGQVD
NP_116242                           ------FEVGMLVWHKHKKYPFWPAVVKSVRQRDK-----
139221_MUM1L1_NM_152423             ------FETGMIVWFKYQKYPFWPAVIKSIRRKER-----
728317_LOC728317_XP_001127165       ------IERGTMVWFKFQDHPFWPAVVKSVSNTDK-----
728307_LOC728307_XP_001127139       ------IERGTMVWFKFQDHPFWPAVVKSVSNTDK-----
27154_BRPF3_NM_015695               ------LEPLELVWAKCRGYPSYPALIIDPKMPREGLLHN
7862_BRPF1_NM_001003694             ------LDALDLVWAKCRGYPSYPALIIDPKMPREGMFHH
23774_BRD1_NM_014577                ------LEPLKVVWAKCSGYPSYPALIIDPKMPRVPGHHN
55063_ZCWPW1_NM_017984              ----ASYIPGSIIWAKQYGYPWWPGMIESDPDLGEYFLFT
2956_MSH6_HSU54777                  ----CDFSPGDLVWAKMEGYPWWPCLVYNHPFDGTFIREK
                                               ::  *      :*

3068_HDGF_NP_004485                 T-----ANKYQVFFFGT-HETAFLGPKDLFPY-EESKEKF
50810_HDGFRP3_CCDS32314             P-----ANKYPIFFFGT-HETAFLGPKDLFPY-KEYKDKF
84717_HDGFRP2_UNQ785PRO1604         P-----PNKYPIFFFGT-HETAFLGPKDLFPY-DKCKDKY
154150_HDGFL1_NP_612641             ------PNRYQVFFFGT-HETAFLSPKRLFPY-KECKEKF
XP_001634069_Nv                     ------AKKYPIFFYGT-HETAVMLPKDLFPY-EKHKHKF
84656_GLYR1_NM_032569               R---G-KKCFFVKFFGT-EDHAWIKVEQLKPY-HAHKEEM
7468_a_WHSC1_NM_133330_WHSCr        K---HEIGEFPVFFFGS-KDYYWTHQARVFPYMEGDRGSR
64324_a_NSD1_NM_172349_WHSCr        R---HDVGEFPVLFFGS-NDYLWTHQARVFPYMEGDVSSK
54904_a_WHSC1L1_NM_023034_WHSC      K---HDLGDFPVFFFGS-HDYYWVHQGRVFPYVEGDK-SF
XP_001635219_Nv                     R---HQPGEFPVMFLGS-HDFYWIHKGRVFSYQDGDKGTE
114825_PWWP2Aisob_NP_001124336      L---LVRQEARISWFGS-PTTSFLALSQLSPFLENFQSRF
170394_PWWP2B_NM_138499             E---PSWREAKVSWFGS-PTTSFLSISKLSPFSEFFKLRF
XP_001624451_Nv                     G----VDTFAHVTWFGS-NTSSEMPVQELQDFEPNFRRRY
1788_DNMT3A_NM_022552               A-----EGTRWVMWFGD-GKFSVVCVEKLMPLSS-FCSAF
1789_DNMT3B_NM_006892               M-----SGMRWVQWFGD-GKFSEVSADKLVALGL-FSQHF
XP_001626231_Nv                     G-----PGNHWVKWFGD-NKFSQVTYDTVLPFAE-FKSNF
54904_b_WHSC1L1_NM_023034_MSH6      ---TRGAREYHVQFFSNQPERAWVHEKRVREYKGHKQYEE
```

```
7468_b_WHSC1_NM_133330_MSH6l          G-QKKSARQYHVQFFGDAPERAWIFEKSLVAFEGEGQFEK
64324_b_NSD1_NM_172349_MSH6l          VSNRRPYRQYYVEAFGDPSERAWVAGKAIVMFEGRHQFEE
152098_ZCWPW2_NP_001035522            P-DGNV-EEYHIEFLGDPHSRSWIKATFVGHYSITLKPEK
XP_001633052_Nv                       L-DDRIPISYHVVFFGKDVSRAWVNSFSVRELTADEEPED
10771_ZMYND11_NM_006624               -----------VRFFGHHHQRAWIPSENIQDITVNIHRLH
XP_001625954_Nv                       A---------LVRFFGSAHQR-------------------
23613_ZMYND8_NM_183047                -----------ARFFG-QHDRAWVPINNCYLMSKEIPFSV
NP_116242                             --------KASVLYIEGHMNPKMKG---FTVSLKSLKHFD
139221_MUM1L1_NM_152423               --------KASVLFVEANMNSEKKG---IRVNFRRLKKFD
728317_LOC728317_XP_001127165         --------TARVLLLEANLHHGKRG---IQVPLRRLKHLD
728307_LOC728307_XP_001127139         --------TARVLLLEANLHHGKRG---IQVPLRRLKHLD
27154_BRPF3_NM_015695                 G-VPIPVPPLDVLKLGEQKQAEAGEKLFLVLFFDNKRTWQ
7862_BRPF1_NM_001003694               G-VPIPVPPLEVLKLGEQMTQEAREHLYLVLFFDNKRTWQ
23774_BRD1_NM_014577                  G-VTIPAPPLDVLKIGEHMQTKSDEKLFLVLFFDNKRSWQ
55063_ZCWPW1_NM_017984                SHLDSLPSKYHVTFFGETVSRAWIPVNMLKNFQELSLELS
2956_MSH6_HSU54777                    G----KSVRVHVQFFDDSPTRGWVSKRLLKPYTGSKSKEA


3068_HDGF_NP_004485                   GKPN-----------K--RKGFSEGLWEIENN--------
50810_HDGFRP3_CCDS32314               GKSN-----------K--RKGFNEGLWEIENN--------
84717_HDGFRP2_UNQ785PRO1604           GKPN-----------K--RKGFNEGLWEIQNN--------
154150_HDGFL1_NP_612641               GKPN-----------K--RRGFSAGLWEIENN--------
XP_001634069_Nv                       AKPC-----------K--RKGFMEALEEIVKN--------
84656_GLYR1_NM_032569                 IKIN-----------K--GKRFQQAVDAVEEFL-------
7468_a_WHSC1_NM_133330_WHSCr          YQGV-----------RGIGRVFKNALQEAEARFREI----
64324_a_NSD1_NM_172349_WHSCr          DKMG-----------KGVDGTYKKALQEAAARFEEL----
54904_a_WHSC1L1_NM_023034_WHSC        AEGQ-----------TSINKTFKKALEEAAKRFQEL----
XP_001635219_Nv                       SGNN-----------KYLAKVFKKALVEAKEKYDEW----
114825_PWWP2Aisob_NP_001124336        NK--------------KRKGLYRKAITEAAKAA-------
170394_PWWP2B_NM_138499               NR--------------KKKGMYRKAITEAANAA-------
XP_001624451_Nv                       KK--------------DKKGCYRRAVRQAQETL-------
1788_DNMT3A_NM_022552                 HQAT-----------YNKQPMYRKAIYEVLQVA-------
1789_DNMT3B_NM_006892                 NLAT-----------FNKLVSYRKAMYHALEKA-------
XP_001626231_Nv                       LVTK-----------MKG--LYKKAVLDALEVL-------
54904_b_WHSC1L1_NM_023034_MSH6        LLAE-----------ATKQASNHSEKQKIRKPRPQRERAQ
7468_b_WHSC1_NM_133330_MSH6l          LCQE-----------SAKQAPTKAEKIKLLKPISGKLRAQ
64324_b_NSD1_NM_172349_MSH6l          LP-------------VLRRRGKQKEK-GYRHKVPQKILSK
152098_ZCWPW2_NP_001035522            CKN--------------KKKWYKSALQEA-----------
XP_001633052_Nv                       IGNI-----------VVRKKNYKKQLDDANGQARQALSLP
10771_ZMYND11_NM_006624               VKR---------------SMGWKKACDELELH--------
XP_001625954_Nv                       ----------------------------------------
23613_ZMYND8_NM_183047                KKT---------------KSIFNSAMQEMEVY--------
NP_116242                             CK--------------EKQTLLNQAREDFNQDIGWC----
139221_MUM1L1_NM_152423               CK--------------EKQMLVDKAREDYSESIDWC----
728317_LOC728317_XP_001127165         CK--------------EKEKLLKRAQKAYKQSVNWC----
728307_LOC728307_XP_001127139         CK--------------EKEKLLKRAQKAYKQSVNWC----
27154_BRPF3_NM_015695                 WLPRDKVLPLGVEDTVDKLKMLEGRKTSIRKSVQVAYDRA
7862_BRPF1_NM_001003694               WLPRTKLVPLGVNQDLDKEKMLEGRKSNIRKSVQIAYHRA
23774_BRD1_NM_014577                  WLPKSKMVPLGIDETIDKLKMMEGRNSSIRKAVRIAFDRA
55063_ZCWPW1_NM_017984                VMKK-------------RRNDCSQKLGVALMMAQEAEQIS
2956_MSH6_HSU54777                    QKGG---------HFYSAKPEILRAMQRADEALNKDKIKR


3068_HDGF_NP_004485                   ----------------
50810_HDGFRP3_CCDS32314               ----------------
84717_HDGFRP2_UNQ785PRO1604           ----------------
154150_HDGFL1_NP_612641               ----------------
```

```
XP_001634069_Nv                    ----------------
84656_GLYR1_NM_032569              ----------------
7468_a_WHSC1_NM_133330_WHSCr       ----------------
64324_a_NSD1_NM_172349_WHSCr       ----------------
54904_a_WHSC1L1_NM_023034_WHSC     ----------------
XP_001635219_Nv                    ----------------
114825_PWWP2Aisob_NP_001124336     ----------------
170394_PWWP2B_NM_138499            ----------------
XP_001624451_Nv                    ----------------
1788_DNMT3A_NM_022552              ----------------
1789_DNMT3B_NM_006892              ----------------
XP_001626231_Nv                    ----------------
54904_b_WHSC1L1_NM_023034_MSH6     WDIGIAHAEKALKMTR
7468_b_WHSC1_NM_133330_MSH6l       WEMGIVQAEEAASMSV
64324_b_NSD1_NM_172349_MSH6l       WEASVGLAE-------
152098_ZCWPW2_NP_001035522         ----------------
XP_001633052_Nv                    VQERLAV---------
10771_ZMYND11_NM_006624            ----------------
XP_001625954_Nv                    ----------------
23613_ZMYND8_NM_183047             ----------------
NP_116242                          ----------------
139221_MUM1L1_NM_152423            ----------------
728317_LOC728317_XP_001127165      ----------------
728307_LOC728307_XP_001127139      ----------------
27154_BRPF3_NM_015695              MIH-------------
7862_BRPF1_NM_001003694            LQH-------------
23774_BRD1_NM_014577               MNH-------------
55063_ZCWPW1_NM_017984             I---------------
2956_MSH6_HSU54777                 LELAV-----------
```

Multiple sequence alignment of *Arabidopsis thaliana* PWWP-domain containing sequences. The multiple sequence alignment was used to reconstruct the maximum likelihood (AF4) and neighbor joining (AF5) phylogenies.

```
NP_850170_ATX1_At2g31650          --FEPGDIVWAKLAG-HAMWPAVIVDESIIGERKGLNNKV
NP_172074_ATX2_AT1G05830          --FEPRDIIWAKLTG-HAMWPAIIVDESVIVKRKGLNNKI
NP_195915_At_AT5G02950            ------DLVWAKLRS-YPWWPGLVFD-KSVASKAAMRHFK
NP_187578_At_At3g09670            --ISDSDLVWAKVRS-HPWWPGQVFD-ASAATDKAKKHFK
NP_190402_ATMlike_AT3G48190       -----GNLVWVMTKY-KKWWPGEVVD-FKADAKES-----
NP_187194_At_AT3G05430            --FEVGDMVWGKVKS-HPWWPGQIFN-EAFASPSVRRVKK
NP_198117_At_AT5G27650            --FEVGDLVWGKVKS-HPWWPGHIFN-EAFASPSVRRMRR
NP_198850_At_AT5G40340            -----GDFVWGKIKN-HPWWPGQIYD-PSDASDLALKIKQ
NP_189424_At_AT3G27860            --FHVGDFVWGEEANSQQWWPGQIYD-SLDASDLALKTMQ
NP_194520_ATX4_WHCS1r_AT4G2791    --FYSGDLVWAKSGRNEPFWPAIVIDPMTQAPELVLRSCI
NP_200155_ATX5_At_AT5G53430       --FYSGDLVWGKSGRNEPFWPAIVIDPMTQAPELVLRSCI
NP_191733_ATX3_WHCS1r_AT3G6174    --FTVGDLVWAKCGKRFPAWPAVVIDPISQAPDGVLKHCV
NP_191866_At_HDGFr_AT3G63070      -EWKVGDLVLAKVKG-FPAWPAVVDEPEKWGHSADSK---
NP_850485_At_HDGFr_At2g48160      -----GDLVLAKVKG-FPAWPAVVSEPEKWDASPDSK---
NP_197706_HUA2_At_HDGFr_AT5G23    -QLVLGDLVLAKVKG-FPAWPAKISRPEDWDRAPDPK---
NP_196440_At_AT5G08230            GEMRLGDLVLAKVKG-FPAWPAKIGQPEDWNQAPDPK---

NP_850170_ATX1_At2g31650          SGGGSLLVQFFGTHD------FARIKVKQAISFIKGLLSP
NP_172074_ATX2_AT1G05830          SGGRSVLVQFFGTHD------FARIQVKQAVSFLKGLLSR
NP_195915_At_AT5G02950            KG--NVLVAYFGDCT-----FA-WNNASQIKPFHQNFSQM
NP_187578_At_At3g09670            KG--SFLVTYFGDCT-----FA-WNEASRIKPFRQHFSQM
NP_190402_ATMlike_AT3G48190       -----FMVRSIGQSH-----LVSWFASSKLKPFKESFEQV
NP_187194_At_AT3G05430            MG--YVLVAFFGDN------SYGWFDPAELIPFEPHVKEK
NP_198117_At_AT5G27650            ID--HVLVAFFGDS------SYGWFDPAELIPFEPNLEEK
NP_198850_At_AT5G40340            KG--KLLVACFGDG------TFAWCGASQLKPFAESFKEC
NP_189424_At_AT3G27860            KG--KLLVAYFGDGS-----FFGWCNPLELKPFLENFKEF
NP_194520_ATX4_WHCS1r_AT4G2791    PD--AACVVFFGHSGNENERDYAWVRRGMIFPFVDYVARF
NP_200155_ATX5_At_AT5G53430       PD--AACVMFFGHSGTENERDYAWVRRGMIFPFVDYVERL
NP_191733_ATX3_WHCS1r_AT3G6174    PG--AICVMFFGYSKDGTQRDYAWVRQGMVYPFTEFMDKF
NP_191866_At_HDGFr_AT3G63070      ----KVTVHFFGTQQ------IAFCNHGDVESFTEEKKQS
NP_850485_At_HDGFr_At2g48160      ----KVFVHFFGTQQ------IAFCNPGDVEAFTEERKQS
NP_197706_HUA2_At_HDGFr_AT5G23    ----KYFVQFFGTEE------IAFVAPPDIQAFTSEAKSK
NP_196440_At_AT5G08230            ----KHFVQFYGTGE------IGFVTPPDIQPFTSETKKK

NP_850170_ATX1_At2g31650          SHLK----CKQPRFEEGMQEA-------
NP_172074_ATX2_AT1G05830          SPLK----CKQPRFEEAMEEA-------
NP_195915_At_AT5G02950            QEQ-----SNSAEFRDAIDCALDE----
NP_187578_At_At3g09670            AKQ-----SSLPDFIDAIDFALEE----
NP_190402_ATMlike_AT3G48190       LNQ-----RNDNGFFDALQKA-------
NP_187194_At_AT3G05430            SQQ-----TSSDHFAKAVEEAMNE----
NP_198117_At_AT5G27650            SQQQ----TVSKHFVRAVEEAKDE----
NP_198850_At_AT5G40340            SKV-----SNSRSFLGAVEEAVEE----
NP_189424_At_AT3G27860            SKM-----SDSRRFLLAVEDA-------
NP_194520_ATX4_WHCS1r_AT4G2791    QEQPELQGCKPGNFQMALEEAFLADQGF
NP_200155_ATX5_At_AT5G53430       QEQSELRGCNPRDFQMALEEALLADQGF
NP_191733_ATX3_WHCS1r_AT3G6174    QDQTNLFNYKASEFNKALEEAVLAENGN
NP_191866_At_HDGFr_AT3G63070      LLTR-RHAKGSD-FVRAVKEITES----
NP_850485_At_HDGFr_At2g48160      LLTR-RHAKGSD-FVRAVKEIIES----
NP_197706_HUA2_At_HDGFr_AT5G23    LLAR-CQGKTVKYFAQAVEQICTA----
NP_196440_At_AT5G08230            LSAR-CQGKTVKYFSQAVEEI-------
```

Multiple sequence alignment of plant and algae PWWP-domain containing sequences. The multiple sequence alignment was used to reconstruct the neighbor joining (AF8) phylogeny.

```
XP_002318650_Populus                ----------------FHVGDIVWVKTKN---QSWWPGKI
XP_002461281_Sorghum                ----------------FAPGDMVWGKKLN---HAAWPGLI
NP_189424_At_AT3G27860              ----------------FHVGDFVWGEEANS--QQWWPGQI
XP_002875407_Alyrata                ----------------FHVGDFVWGKEVNS--QQWWPGQI
NP_198850_At_AT5G40340              -------------------GDFVWGKIKN---HPWWPGQI
XP_002305994_Populus                ----------------FRVGDFVWGKIKS---HPWWPGRV
NP_195915_At_AT5G02950              --------------------DLVWAKLRS---YPWWPGLV
XP_002871003_Alyrata                --------------------DLVWAKIRS---YPWWPGQV
NP_187578_At_At3g09670              ----------------ISDSDLVWAKVRS---HPWWPGQV
XP_002882633_Alyrata                ----------------ISDSDLVWAKVRS---HPWWPGQV
XP_002326168_Populus                ----------------LSVGDLVWGKVRS---HPWWPGQV
XP_002322856_Populus                -------------------GDLVWGKVRS---HPWWPGQL
XP_002315275_Populus                ----------------FSVSDLVWGKVRS---HPWWPGQI
XP_002439929_Sorghum                --------------------DLVWGKVKS---HPWWPGEI
XP_002440507_Sorghum                ------------------PGRLVWGKVRD---HPWWPAQV
NP_187194_At_AT3G05430              ----------------FEVGDMVWGKVKS---HPWWPGQI
XP_002882413_Alyrata                ----------------FEVGDMVWGKVKS---HPWWPGQI
XP_002319529_Populus                ----------------FEVGDMVWGKVKS---HPRWPGHI
XP_002330150_Populus                ----------------FEVGDMVWGKVKS---HPWWPGHI
NP_198117_At_AT5G27650              ----------------FEVGDLVWGKVKS---HPWWPGHI
XP_002330341_Populus                ----------------------------------------
XP_002439285_Sorghum                ----------------PRFGDMVWAKVKS---HPWWPGHI
XP_002878409_Alyrata                ----------------FTVGDLVWAKCGKR--FPAWPAVV
XP_002878396_Alyrata                ---------------------------------PAWPAMV
NP_191733_ATX3_WHCS1r_AT3G6174      ----------------FTVGDLVWAKCGKR--FPAWPAVV
XP_002302629_Populus                ----------------FALGDIVWAKCGKR--YPWWPAIV
NP_194520_ATX4_WHCS1r_AT4G2791      ----------------FYSGDLVWAKSGRN--EPFWPAIV
XP_002869532_Alyrata                ----------------FYSGDLVWAKSGRN--EPFWPAIV
NP_200155_ATX5_At_AT5G53430         ----------------FYSGDLVWGKSGRN--EPFWPAIV
XP_002864242_Alyrata                ----------------FYSGDLVWGKSGRK--EPFWPAIV
XP_002318412_Populus                ----------------FYSGDLVWAKSGMK--YPFWPAIV
XP_002321418_Populus                ----------------FYSGDIVWAKSGNK--YPFWPAIV
XP_002454931_Sorghum                ----------------FVLGDVVWARSGKK--SPTWPALV
XP_002961390_Selaginella            ----------------FDIGDIVWAKSGKKK-DPVWPAKV
XP_002980880_Selaginella            ----------------FDIGDIVWAKSGKKK-DPVWPAKV
XP_001777592_Physcomitrella         ----------------FDLGEIVWAKSGKRN-DPVWPARV
XP_002456070_Sorghum                ----------------LVYGDIVWAKLGKR--QPMWPGVL
XP_003078017_O_tauri                -----------------VHGDVVFARASSRATEPLWPAVV
NP_191866_At_HDGFr_AT3G63070        ---------------EWKVGDLVLAKVKG---FPAWPAVV
NP_850485_At_HDGFr_At2g48160        -------------------GDLVLAKVKG---FPAWPAVV
XP_002880345_Alyrata                ---------------KWKVGDLVLAKVKG---FPAWPAAV
XP_002876705_Alyrata                -----------------KVGDLVLAKVKG---FPAWPAAV
XP_002465241_Sorghum                ----------------WKVGDLVLAKMKG---FPAWPAMI
XP_002463300_Sorghum                ---------------QWKVGDLVLAKMKG---FPAWPAMQ
NP_197706_HUA2_At_HDGFr_AT5G23      ---------------QLVLGDLVLAKVKG---FPAWPAKI
XP_002872040_Alyrata                --------------GQLILGDLVLAKVKG---FPAWPAKI
NP_196440_At_AT5G08230              --------------GEMRLGDLVLAKVKG---FPAWPAKI
XP_002873347_Alyrata                ----------------LRLGDLVLAKVKG---FPAWPAKI
XP_002310078_Populus                ---------------QLRLGDLVLAKVKG---YPSWPAKI
XP_002306384_Populus                ---------------QLRLGDLVLAKVKG---YPSWPAKI
XP_002443678_Sorghum                --------------REPQLGDLVLAKIKG---YPPWPAKI
XP_002974023_Selaginella            ---------------QWKIGDLVLAKVKG---FPAWPAQV
```

```
XP_002983468_Selaginella          ---------------QWKIGDLVLAKVKG---FPAWPAQV
XP_002962776_Selaginella          ---------------EWSVGDLVLAKVKG---FPAWPAQV
XP_001753837_Physcomitrella       --------------REFNEGDLVLAKVKG---WPAWPAQA
XP_001765392_Physcomitrella       --------------REFNVGDLVLAKVKG---WPAWPAQ-
NP_850170_ATX1_At2g31650          ----------------FEPGDIVWAKLAG---HAMWPAVI
XP_002881186_Alyrata              ----------------FETGDIVWAKLAG---HAMWPAVI
NP_172074_ATX2_AT1G05830          ----------------FEPRDIIWAKLTG---HAMWPAII
XP_002889570_Alyrata              ----------------FEPRDIIWAKLTG---HAMWPAII
XP_002301643_Populus              ----------------LEPGDIIWAKLTG---HAMWPAIV
XP_002320433_Populus              ------------------PGDIIWAKVTG---HAMWPAIV
XP_002459927_Sorghum              ------------------PGDLVWAKITG---HAMWPAVV
XP_002992195_Selaginella          -------------------GTVVWAKVKG---WPMWPALV
XP_002982095_Selaginella          DELALLANEMESFEEQISHGTVVWAKVKG---WPMWPALV
XP_001767466_Physcomitrella       -------------------GDLVWAKVKG---WPMWPAFV
XP_001780587_Physcomitrella       -------------------GDLVWAKVKG---WPMWPAFV
XP_002975241_Selaginella          ----------------FQHGDIVWAETPG---YPMWPAFV
XP_001693149_Chlamydomonas        ----------------LRPGEVVWAQVRR---SAPWPAIV
XP_001760849_Physcomitrella       ----------------FKHGQLVWAKFAR---FPWWPAEI
XP_001751216_Physcomitrella       -------------------GQLVWAKFAR---FPWWPAEI
XP_002961091_Selaginella          -----------------SAGQLVWAKFSR---SPWWPAQV
XP_002966918_Selaginella          -----------------SAGQLVWAKFSR---SPWWPAQV
XP_002444527_Sorghum              ----------------FRLGDITWVKCSG---SSWWPAQV


XP_002318650_Populus              FDPLGVTKYAVQ--SDQR---NGLLVGYLGSC--------
XP_002461281_Sorghum              YSAGGN-----G--TGHE---GQFLVSYFGDK--------
NP_189424_At_AT3G27860            YDSLDASDLALK--TMQK---GKLLVAYFGDG--------
XP_002875407_Alyrata              YDSLDASDLALK--TMQK---GKLLVAYFGDG--------
NP_198850_At_AT5G40340            YDPSDASDLALK--IKQK---GKLLVACFGDG--------
XP_002305994_Populus              YDPSNASDYAKK--VKQR---DKILVAYFGDS--------
NP_195915_At_AT5G02950            FDKSVASKAAMR--HFKK---GNVLVAYFGDC--------
XP_002871003_Alyrata              IDASVASKAAKK--HFKKK--GNLLVAYFGDC--------
NP_187578_At_At3g09670            FDASAATDKAKK--HFKK---GSFLVTYFGDC--------
XP_002882633_Alyrata              FDASAATDKAKK--YFKK---GSFLVTYFGDC--------
XP_002326168_Populus              FGRSDASKKAKK--YFKK---NSYLIAYFGDQ--------
XP_002322856_Populus              FGCADASKKAKK--HFKK---DSYLIAYLGDQ--------
XP_002315275_Populus              FDPSDASEKAMR--YHKK---DCYLVAYFGDR--------
XP_002439929_Sorghum              FDPSDASELALK--HQKK---GSHLVAYFGDN--------
XP_002440507_Sorghum              FDAADASADARA--LRRPR--GAVLVAYFWDK--------
NP_187194_At_AT3G05430            FNEAFAS-PSVR--RVKKM--GYVLVAFFGDN--------
XP_002882413_Alyrata              FNEAFAS-PSVR--RMKKM--GYVLVAFFGDN--------
XP_002319529_Populus              FNEAFAS-SSVR--RTRRE--GHVLVAFFGDS--------
XP_002330150_Populus              FNEAFAS-SSVR--RTRRE--GHVLVAFFGDS--------
NP_198117_At_AT5G27650            FNEAFAS-PSVR--RMRRI--DHVLVAFFGDS--------
XP_002330341_Populus              -----------------RE--GHVLVAFFGDG--------
XP_002439285_Sorghum              YSVNLTDDEEVH--RGYRD--GLVLVAFFGDS--------
XP_002878409_Alyrata              IDPISQAPDGVL--KHCVP--GAICVMFFGYS-KDG-TQR
XP_002878396_Alyrata              VDPISQAPDGVL--KHCVP--GAICVMFFGYS-KNG-TQR
NP_191733_ATX3_WHCS1r_AT3G6174    IDPISQAPDGVL--KHCVP--GAICVMFFGYS-KDG-TQR
XP_002302629_Populus              IDPILQAPDAVL--SCCVP--GAICIMFYGYS-KNG-TQR
NP_194520_ATX4_WHCS1r_AT4G2791    IDPMTQAPELVL--RSCIP--DAACVVFFGHS-GNE-NER
XP_002869532_Alyrata              IDPMTQAPELVL--RSCIP--DAACVVFFGHS-GNE-NER
NP_200155_ATX5_At_AT5G53430       IDPMTQAPELVL--RSCIP--DAACVMFFGHS-GTE-NER
XP_002864242_Alyrata              IDPMTQAPELVL--RSCIP--DAACVMFFGHS-GTE-NER
XP_002318412_Populus              IDPMTQAPELVL--RSCIA--DAACVMFFGCS-GNDGDQR
XP_002321418_Populus              IDPMTQAPELVL--RSCIA--DAACVMFFGCS-GNDGNQR
XP_002454931_Sorghum              IDPMQHAPEVVL--NSCVP--GALCVMFFGYS-ANG-HGR
```

```
XP_002961390_Selaginella          VDPIKEVPESVR--RKCVP--NRLCVMFYGESLARGVKRR
XP_002980880_Selaginella          VDPIKEVPESVR--RKCVP--NRLCVMFYGESLARGVKRR
XP_001777592_Physcomitrella       IDPFREAPPMVR--ELSLP--NSLCVMFYGPSSSKGKHSR
XP_002456070_Sorghum              IDPTQQVAADAMPPQPRRV--AVLCVMLFGWCTEFS-DEK
XP_003078017_O_tauri              VEPW-DAPEGVR--KQCEP--ESVCVMFLGPSATRG-RAR
NP_191866_At_HDGFr_AT3G63070      D-------EPEKWGHSADS--KKVTVHFFGT---------
NP_850485_At_HDGFr_At2g48160      S-------EPEKWDASPDS--KKVFVHFFGT---------
XP_002880345_Alyrata              S-------EPEKWGALTDL--KKVFVHFFGT---------
XP_002876705_Alyrata              S-------QPEKWGYSADS--KKVLVHFFGT---------
XP_002465241_Sorghum              S-------EPEQWKMPSTK--KKPLVYFYGT---------
XP_002463300_Sorghum              ----------------FIK--YSVHINIYRS---------
NP_197706_HUA2_At_HDGFr_AT5G23    S-------RPEDWDRAPDP--KKYFVQFFGT---------
XP_002872040_Alyrata              S-------RPEDWDRAPDP--KKYFVQFFGT---------
NP_196440_At_AT5G08230            G-------QPEDWNQAPDP--KKHFVQFYGT---------
XP_002873347_Alyrata              G-------QPEDWNQAPDP--KKHFVQFFGT---------
XP_002310078_Populus              S-------RPEDWKRVADA--KKVFVYFFGT---------
XP_002306384_Populus              S-------RPEDWKRAPDA--KKVFVYFFGT---------
XP_002443678_Sorghum              S-------RPEDWQHKPTP--KKFFVYFYGT---------
XP_002974023_Selaginella          S-------RAEDFGRQRNP--AKVFVVFFGT---------
XP_002983468_Selaginella          S-------RAEDFGRQRNP--AKVFVVFFGT---------
XP_002962776_Selaginella          S-------KPEAFGQHHNP--ARVFVVFFGT---------
XP_001753837_Physcomitrella       EVSCFPQEIGKLETQGKHA--GKYYVTYFGPG--------
XP_001765392_Physcomitrella       --------IGRLETQGKHA--GKYYVTYFGPG--------
NP_850170_ATX1_At2g31650          VDESIIGERKGLNNKVSGGG--SLLVQFFGTH--------
XP_002881186_Alyrata              VDESVIGERKGLNNKVSGGG--SLLVQFFGTH--------
NP_172074_ATX2_AT1G05830          VDESVIVKRKGLNNKISGGR--SVLVQFFGTH--------
XP_002889570_Alyrata              VDESVIVKRKGLNNKASGGR--SVLVQFFGTH--------
XP_002301643_Populus              VDGALIGDHKGIS-KNIGGG--SISVQFFGTH--------
XP_002320433_Populus              VDEALIGNHKGLS-KNIGGR--SVSVQFFGTH--------
XP_002459927_Sorghum              VDESNVPATRALK-SIRLDQ--SILVQFFGTH--------
XP_002992195_Selaginella          LDEE-HAEKCGLERPLKKS---TFAVQFFGSC--------
XP_002982095_Selaginella          LDEE-HAEKCGLERPLKKS---TFAVQFFGSC--------
XP_001767466_Physcomitrella       MDED-HAAACGME-PGKKG---MVPLQFFGSY--------
XP_001780587_Physcomitrella       MDED-HAAACGMD-PGKKG---MVPLQFFGSY--------
XP_002975241_Selaginella          MDDQ-HARLCNLE--ISTG---ELPVHYFGTY--------
XP_001693149_Chlamydomonas        ITRE-EAEREGLVGGSRLGHNHQVFVRFFGDY--------
XP_001760849_Physcomitrella       INERAFQPGTGGNRRSNTD----VLVRFFGTY--------
XP_001751216_Physcomitrella       INEKAVIPGTGGNKRINAD----VLSKCF-IY--------
XP_002961091_Selaginella          ADEKSMSERT---RQSKNH----VLVRFFGNY--------
XP_002966918_Selaginella          ADEKSMSERT---RQSKNH----VLVRFFGNY--------
XP_002444527_Sorghum              IDELCVGSKP--KKKDKYD----CLVRLYGTC--------


XP_002318650_Populus              HIA-------WCLPSQLKPFHKDFEQMVVKN---------
XP_002461281_Sorghum              AFA-------WCDGAELRPYEPYFPVAELYD--------D
NP_189424_At_AT3G27860            SFFG------WCNPLELKPFLENFKEFSKMS---------
XP_002875407_Alyrata              KFC-------WCNPLELKPFLENFKEFSKMS---------
NP_198850_At_AT5G40340            TFA-------WCGASQLKPFAESFKECSKVS---------
XP_002305994_Populus              TFA-------WCNPSQLSPFEENFVEMFKQS---------
NP_195915_At_AT5G02950            TFA-------WNNASQIKPFHQNFSQMQEQS---------
XP_002871003_Alyrata              TFA-------WNNASQVKPFHQNFSQMQEQS---------
NP_187578_At_At3g09670            TFA-------WNEASRIKPFRQHFSQMAKQS---------
XP_002882633_Alyrata              TFA-------WNDASRVKPFRQHFSQMAKQS---------
XP_002326168_Populus              TFA-------WNEVSKIKPFRCNFSLLEKQS---------
XP_002322856_Populus              TFA-------WNEVSKIKPFRCNFSLLEKQS---------
XP_002315275_Populus              TFA-------WNEASLLKPFRSHFSQVEKQS---------
XP_002439929_Sorghum              TFA-------WCDESQLKPFVTNYSQMEKQS---------
```

XP_002440507_Sorghum	TFA-------WNDAAALLPFRAGF----------------
NP_187194_At_AT3G05430	SYG-------WFDPAELIPFEPHVKEKSQQ---------T
XP_002882413_Alyrata	SYG-------WFDPAELLPFEPHVAENSQQ---------T
XP_002319529_Populus	SYG-------WFDPAELIQFDVNFAEKSQQ---------T
XP_002330150_Populus	SYG-------WFDPAELIPFDANFAEKSQQ---------T
NP_198117_At_AT5G27650	SYG-------WFDPAELIPFEPNLEEKSQQQ--------T
XP_002330341_Populus	SYG-------WLDPTELVQFDSHYVEKSKQT--------N
XP_002439285_Sorghum	SYG-------WFEPSELVPFEDHFTEK-------------
XP_002878409_Alyrata	DYA-------WVRQGMMYPFTEFMDKFQDQTNLY----NY
XP_002878396_Alyrata	DYA-------WVRQGMVYPFTEFMDKFQDKTNLY----NY
NP_191733_ATX3_WHCS1r_AT3G6174	DYA-------WVRQGMVYPFTEFMDKFQDQTNLF----NY
XP_002302629_Populus	DYA-------WVKQGMVFPFAEFMERFQVQSQMF----KC
NP_194520_ATX4_WHCS1r_AT4G2791	DYA-------WVRRGMIFPFVDYVARFQEQPELQ----GC
XP_002869532_Alyrata	DYA-------WVRRGMIFPFVDYVARFQEQSELQ----GC
NP_200155_ATX5_At_AT5G53430	DYA-------WVRRGMIFPFVDYVERLQEQSELR----GC
XP_002864242_Alyrata	DYA-------WVRRGMIFPFVDYVDRFQEQSELR----GC
XP_002318412_Populus	DYA-------WVQRGMIFPFLDFVDRFQEQSELD----DC
XP_002321418_Populus	DYA-------WVQRGMIFPFMDFLDRFQEQSELD----DF
XP_002454931_Sorghum	DYG-------WVKQGMIFPFVDYLDRFQGQP-LY----KL
XP_002961390_Selaginella	DYA-------WVRKGMIFPFVDYLERFQSQTSFN----KS
XP_002980880_Selaginella	DYA-------WVRKGMIFPFVDYLERFQSQTSFN----KS
XP_001777592_Physcomitrella	DYA-------WVKQGMIFPFNAYLERFQSQTQLN----RS
XP_002456070_Sorghum	KYV-------WVRQGLIFPFSDYMDRFQGQTELS----SC
XP_003078017_O_tauri	DYC-------WATEERLAPYAR-AEALFAQKVAK----RM
NP_191866_At_HDGFr_AT3G63070	QQIA------FCNHGDVESFTEEKKQSLLTRRH------A
NP_850485_At_HDGFr_At2g48160	QQIA------FCNPGDVEAFTEERKQSLLTRRH------A
XP_002880345_Alyrata	QQIA------FCNHTDVEAFTDERKQSLLTKRH------A
XP_002876705_Alyrata	QQIA------LCNPADVESFTEEKKQLLLTKRH------A
XP_002465241_Sorghum	KQIA------FCNYADLEAFTEEKKRSLLAKRH------G
XP_002463300_Sorghum	QDIV------EITEPEQWDLPAAKKKSIVAKK--------
NP_197706_HUA2_At_HDGFr_AT5G23	EEIA------FVAPPDIQAFTSEAKSKLLARC-----QGK
XP_002872040_Alyrata	EEIA------FVAPPDIQAFTSEAKSKLLARC-----QGK
NP_196440_At_AT5G08230	GEIG------FVTPPDIQPFTSETKKKLSARC-----QGK
XP_002873347_Alyrata	QEIG------FVAPPDIQPFTSEAKNKLLARC-----QGK
XP_002310078_Populus	QEIA------FVAPSDIQVFTNEVKNKLSARC-----QSK
XP_002306384_Populus	QEIA------FVAPSDIQVFTNEVKNKLSARC-----QSK
XP_002443678_Sorghum	EEIA------FVPLADLEEFTEKTKNDLLDRAPNIKVQRK
XP_002974023_Selaginella	KQIR------FCQHSEISAFTVEARAALHARTLS----KC
XP_002983468_Selaginella	KQIR------FCQHSEISAFTVEARAALHARTLS----KC
XP_002962776_Selaginella	KQIR------FCHHSEISKFTPEAKASLVAKAHS----KC
XP_001753837_Physcomitrella	QQIG------WCLPVELSEFDADQREASIQKSLK----KT
XP_001765392_Physcomitrella	QQIG------WCLPVELSEFDADQREASVQKSLK----KT
NP_850170_ATX1_At2g31650	DFAR------IKVKQAISFIKGLLSPSHLKCKQP------
XP_002881186_Alyrata	DFAR------IKEKQAISFIKGLLSPSHLKCKQP------
NP_172074_ATX2_AT1G05830	DFAR------IQVKQAVSFLKGLLSRSPLKCKQP------
XP_002889570_Alyrata	DFAR---------EDCHDFEPRDIIWAKLTGHA-------
XP_002301643_Populus	DFAR------IKPKQAISFLKGLLSSFHLKCKQP------
XP_002320433_Populus	DFAR------YLSEQKLP----------------------
XP_002459927_Sorghum	DFAR------F-----------------------------
XP_002992195_Selaginella	DFAR------LNNDKIVTFSKGVQLKYHSKCKRP------
XP_002982095_Selaginella	DFAR------LNNDKIVTFSKGVQLKYHSKCKRP------
XP_001767466_Physcomitrella	DHCR------FSYKKLVIFSKGLMMKFHTKCKRV------
XP_001780587_Physcomitrella	DHCR------FSYKKLVIFSKGLMMKFHTKCKRV------
XP_002975241_Selaginella	ESA-------------------------------------
XP_001693149_Chlamydomonas	TVFA------LPAISAGRIPD--------RGAVV------
XP_001760849_Physcomitrella	DYG-------WVDPEVGLSEFDVKMQERSRIKKK------
XP_001751216_Physcomitrella	GLGSDGKCSGWVDPKVGLSEFDVKLQERSKIKKK------

```
XP_002961091_Selaginella          NYG-------WVDPASDLSKFDVDFDGRSQVPTK------
XP_002966918_Selaginella          NYG-------WVDPASDLSKFDVDFDGRSQVPTK------
XP_002444527_Sorghum              QYL-------YVDPWKSNSEFEMMLKQENKSTME------


XP_002318650_Populus              KARSFLGAVEKAVDE----
XP_002461281_Sorghum              GGEDFDAAVEASLDE----
NP_189424_At_AT3G27860            DSRRFLLAVEDA-------
XP_002875407_Alyrata              ESKRFLSAVEEA-------
NP_198850_At_AT5G40340            NSRSFLGAVEEAVEE----
XP_002305994_Populus              NSKSFVNAVKEAVDE----
NP_195915_At_AT5G02950            NSAEFRDAIDCALDE----
XP_002871003_Alyrata              NLAEFRDAIDCALDE----
NP_187578_At_At3g09670            SLPDFIDAIDFALEE----
XP_002882633_Alyrata              SLPDFIDAIDFALEE----
XP_002326168_Populus              NLEDFHDAVHCALDE----
XP_002322856_Populus              NLEDFHDAVHCALDE----
XP_002315275_Populus              NSEVFQNAVDCSLEE----
XP_002439929_Sorghum              TSDAFVGSVNNALEE----
XP_002440507_Sorghum              -------------------
NP_187194_At_AT3G05430            SSDHFAKAVEEAMNE----
XP_002882413_Alyrata              SSGHFAKAVEEAMDE----
XP_002319529_Populus              NSRTFIKAVEEATDE----
XP_002330150_Populus              NSRTFIRAVEEATDE----
NP_198117_At_AT5G27650            VSKHFVRAVEEAKDE----
XP_002330341_Populus              AKVFLIKAVEEADD-----
XP_002439285_Sorghum              -------------------
XP_002878409_Alyrata              KPSEFKKALEEAVLAENG-
XP_002878396_Alyrata              KPSEFKKALDEAVLAENG-
NP_191733_ATX3_WHCS1r_AT3G6174    KASEFNKALEEAVLAENGN
XP_002302629_Populus              KLSDFQVALEEAILAESGF
NP_194520_ATX4_WHCS1r_AT4G2791    KPGNFQMALEEAFLADQGF
XP_002869532_Alyrata              KPGNFQMALEEAFLADQGF
NP_200155_ATX5_At_AT5G53430       NPRDFQMALEEALLADQGF
XP_002864242_Alyrata              NPREFQMALEEALLADQGF
XP_002318412_Populus              KPGDFQMAVEEAFLAEQGF
XP_002321418_Populus              N-GDFQMAFEEAFLAEQGF
XP_002454931_Sorghum              RPSKFRAAIEEAFLAERGF
XP_002961390_Selaginella          QPGDFWSAIEEATLAEAG-
XP_002980880_Selaginella          QPGDFWSAIEEATLAEAG-
XP_001777592_Physcomitrella       RPVDFRQAISEAKLAD---
XP_002456070_Sorghum              KPADFRRAVEEAFLADQGF
XP_003078017_O_tauri              RPNAFNEACAEA-------
NP_191866_At_HDGFr_AT3G63070      KGSDFVRAVKEITES----
NP_850485_At_HDGFr_At2g48160      KGSDFVRAVKEIIES----
XP_002880345_Alyrata              KGSDFLRAVKEIIES----
XP_002876705_Alyrata              KGSDFVRAVKEITES----
XP_002465241_Sorghum              KGADFLRAVDEIVE-----
XP_002463300_Sorghum              KLLVYFYGTKE--------
NP_197706_HUA2_At_HDGFr_AT5G23    TVKYFAQAVEQICTA----
XP_002872040_Alyrata              TVKYFAQAVEQICTA----
NP_196440_At_AT5G08230            TVKYFSQAVEEI-------
XP_002873347_Alyrata              TVKYFSQAVEEI-------
XP_002310078_Populus              KDRFFSQAVKEICAA----
XP_002306384_Populus              KDKFFSQAVKEICAA----
XP_002443678_Sorghum              YVQVFNDAVEQICKA----
XP_002974023_Selaginella          VPSDLKRAVKEICE-----
```

```
XP_002983468_Selaginella          VPSDLKRAVKEICE-----
XP_002962776_Selaginella          TPADLRLAVSEIC------
XP_001753837_Physcomitrella       ADKKFISAVKEIC------
XP_001765392_Physcomitrella       ADKKFIFAVKEIC------
NP_850170_ATX1_At2g31650          ---RFEEGMQEA-------
XP_002881186_Alyrata              ---RFEEGMQEA-------
NP_172074_ATX2_AT1G05830          ---RFEEAMEEA-------
XP_002889570_Alyrata              -------------------
XP_002301643_Populus              ---RFTRSLEEA-------
XP_002320433_Populus              -------------------
XP_002459927_Sorghum              -------------------
XP_002992195_Selaginella          ---AFDQGLRE--------
XP_002982095_Selaginella          ---AFDQGLRE--------
XP_001767466_Physcomitrella       ---VFVQGLEEVE------
XP_001780587_Physcomitrella       ---VFVQGLEEVE------
XP_002975241_Selaginella          -------------------
XP_001693149_Chlamydomonas        ---PFLSGL----------
XP_001760849_Physcomitrella       ---AFQKGIEEALEY----
XP_001751216_Physcomitrella       ---ALQKGIEEALEF----
XP_002961091_Selaginella          ---AFQKGLKEALEY----
XP_002966918_Selaginella          ---AFQKGLKEALEY----
XP_002444527_Sorghum              ---AFREVLEK--------
```

Multiple sequence alignment of PWWP-domain containing sequences. The multiple sequence alignment was used to reconstruct the neighbor joining (AF9) phylogeny.

```
3068_HDGF_NP_004485                 KEYKCGDLVFAKMKGYPHWP--------------ARI--D
50810_HDGFRP3_CCDS32314             REYKAGDLVFAKMKGYPHWP--------------ARI--D
84717_HDGFRP2_UNQ785PRO1604         HAFKPGDLVFAKMKGYPHWP--------------ARI--D
154150_HDGFL1_NP_612641             --YKSGDLVFAKLKGYAHWP--------------ARI--E
XP_001634069_Nv                     MTYQPGDLIWAKMRGYPHWP--------------ARI--D
84656_GLYR1_NM_032569               --LRLGDLVWGKLGRYPPWP--------------GKI--V
54904_b_WHSC1L1_NM_023034_MSH6      -KFQVGDLVWSKVGTYPWWP--------------CMVSSD
7468_b_WHSC1_NM_133330_MSH6l        LKYNVGDLVWSKVSGYPWWP--------------CMVSAD
64324_b_NSD1_NM_172349_MSH6l        LKYEVGDLIWAKFKRRPWWP--------------CRICSD
152098_ZCWPW2_NP_001035522          ---PLGSLVLVKLQNWPSWP--------------GILCPD
XP_001633052_Nv                     SPFPTGAIVWAKLAGYPWWP--------------AMMEDD
7468_a_WHSC1_NM_133330_WHSCr        --LHFQDIIWVKLGNYRWWP-----------AEVCHPKNV
64324_a_NSD1_NM_172349_WHSCr        --PHYREIVWVKVGRYRWWP-----------AEICHPRAV
54904_a_WHSC1L1_NM_023034_WHSC      --LHYKQIVWVKLGNYRWWP-----------AEICNPRSV
XP_001635219_Nv                     --LLYGDIVWVKLGMYRWWP-----------AMICNPRDV
XP_001747378_b_Mb                   ------DVVWAKYGSYRWWP-----------AVILSATEY
23613_ZMYND8_NM_183047              ---------WF-C--EPCSN--------PHPLVWAKLKGF
10771_ZMYND11_NM_006624             ---------WF-C--YPCIP--------NHELVWAKMKGF
27154_BRPF3_NM_015695               --LEPLELVWAKCRGYPSYPALIIDPKMPREGLLHNGVPI
7862_BRPF1_NM_001003694             --LDALDLVWAKCRGYPSYPALIIDPKMPREGMFHHGVPI
23774_BRD1_NM_014577                --LEPLKVVWAKCSGYPSYPALIIDPKMPRVPGHHNGVTI
114825_PWWP2Aisob_NP_001124336      --ICVGDIVWAKIYGFPWWP--------------ARILTI
170394_PWWP2B_NM_138499             --VAVGDIVWGKIHGFPWWP--------------ARVLDI
XP_001624451_Nv                     --INEGSIIWGKVHGHPWWP--------------GRVMAI
1788_DNMT3A_NM_022552               --FGIGELVWGKLRGFSWWP--------------GRIVSW
1789_DNMT3B_NM_006892               --FGIGDLVWGKIKGFSWWP--------------AMVVSW
XP_001626231_Nv                     -----GVLVWSKLKGYDWWP--------------GRVVTY
NP_116242                           --FEVGMLVWHKHKKYPFWP--------------AVVKSV
139221_MUM1L1_NM_152423             --FETGMIVWFKYQKYPFWP--------------AVIKSI
728317_LOC728317_XP_001127165       --IERGTMVWFKFQDHPFWP--------------AVVKSV
728307_LOC728307_XP_001127139       --IERGTMVWFKFQDHPFWP--------------AVVKSV
55063_ZCWPW1_NM_017984              ASYIPGSIIWAKQYGYPWWP--------------GMIESD
XP_001750279_Mb                     --PQAWDFVWARMRGYPWYP--------------AMLVNW
2956_MSH6_HSU54777                  CDFSPGDLVWAKMEGYPWWP--------------CLVYNH
XP_003084095_O_tauri                -----NQLVWAKLPGHPFWPG------------LRVNLET
XP_003079195_O_tauri                ----------------AVIP--------------ESARTG


3068_HDGF_NP_004485                 EMPEAAVKST--------------ANKYQVFFFG-THETA
50810_HDGFRP3_CCDS32314             ELPEGAVKPP--------------ANKYPIFFFG-THETA
84717_HDGFRP2_UNQ785PRO1604         DIADGAVKPP--------------PNKYPIFFFG-THETA
154150_HDGFL1_NP_612641             HMTQ--------------------PNRYQVFFFG-THETA
XP_001634069_Nv                     LAAKDEVIP---------------AKKYPIFFYG-THETA
84656_GLYR1_NM_032569               NPPKDLKKPRG-------------KKCFFVKFFG-TEDHA
54904_b_WHSC1L1_NM_023034_MSH6      PQLEVHTKIN-----------TRGAREYHVQFFSNQPERA
7468_b_WHSC1_NM_133330_MSH6l        PLLHSYTKLKG---------QKKSARQYHVQFFGDAPERA
64324_b_NSD1_NM_172349_MSH6l        PLINTHSKMKVS--------NRRPYRQYYVEAFGDPSERA
152098_ZCWPW2_NP_001035522          RFKGKYVTYDP---------DGNV-EEYHIEFLGDPHSRS
XP_001633052_Nv                     PDYGYYYETDL---------DDRIPISYHVVFFGKDVSRA
7468_a_WHSC1_NM_133330_WHSCr        ---PPNIQK-----------MKHEIGEFPVFFFG-SKDYY
64324_a_NSD1_NM_172349_WHSCr        ---PSNIDK-----------MRHDVGEFPVLFFG-SNDYL
54904_a_WHSC1L1_NM_023034_WHSC      ---PLNIQG-----------LKHDLGDFPVFFFG-SHDYY
XP_001635219_Nv                     ---PTNIQS-----------MRHQPGEFPVMFLG-SHDFY
```

XP_001747378_b_Mb	---PERCGQ-----------SPAE-GNFLVKFLG-SNDVA
23613_ZMYND8_NM_183047	PFWPAKALRDKD-----G-QVDAR-------FFG-QHDRA
10771_ZMYND11_NM_006624	GFWPAKVMQKED-----N-QVDVR-------FFGHHHQRA
27154_BRPF3_NM_015695	PVPPLDVLKLGE-----QKQAEAGEKLFLVLFFDNKRTWQ
7862_BRPF1_NM_001003694	PVPPLEVLKLGE-----QMTQEAREHLYLVLFFDNKRTWQ
23774_BRD1_NM_014577	PAPPLDVLKIGE-----HMQTKSDEKLFLVLFFDNKRSWQ
114825_PWWP2Aisob_NP_001124336	TVSRKDNGLL-------------VRQEARISWFG-SPTTS
170394_PWWP2B_NM_138499	SLGQKEDGEP-------------SWREAKVSWFG-SPTTS
XP_001624451_Nv	TEVVDDQGG--------------VDTFAHVTWFG-SNTSS
1788_DNMT3A_NM_022552	WMTGRSRAA---------------EGTRWVMWFG-DGKFS
1789_DNMT3B_NM_006892	KATSKRQAM---------------SGMRWVQWFG-DGKFS
XP_001626231_Nv	MEAGRPPPG---------------PGNHWVKWFG-DNKFS
NP_116242	RQRDKKASV-----------------LYIEGHMNPKMKGF
139221_MUM1L1_NM_152423	RRKERKASV-----------------LFVEANMNSEKKGI
728317_LOC728317_XP_001127165	SNTDKTARV-----------------LLLEANLHHGKRGI
728307_LOC728307_XP_001127139	SNTDKTARV-----------------LLLEANLHHGKRGI
55063_ZCWPW1_NM_017984	PDLGEYFLFTS--------HLDSLPSKYHVTFFGETVSRA
XP_001750279_Mb	QTPRDDLAECVPSDAVLKSQPSQDESSLLVRFFDKTGSWA
2956_MSH6_HSU54777	PFDGTFIREKG------------KSVRVHVQFFDDSPTRG
XP_003084095_O_tauri	DFIPEDARKMS------------RDGEVLVVFFG-ENSFG
XP_003079195_O_tauri	GFASKVSDD----------------EHRLVVFFDDKHSFA

3068_HDGF_NP_004485	FLGPKDLFPYEESK--EKFGK---PNKRKGFSEGLWEIEN
50810_HDGFRP3_CCDS32314	FLGPKDLFPYKEYK--DKFGK---SNKRKGFNEGLWEIEN
84717_HDGFRP2_UNQ785PRO1604	FLGPKDLFPYDKCK--DKYGK---PNKRKGFNEGLWEIQN
154150_HDGFL1_NP_612641	FLSPKRLFPYKECK--EKFGK---PNKRRGFSAGLWEIEN
XP_001634069_Nv	VMLPKDLFPYEKHK--HKFAK---PCKRKGFMEALEEIVK
84656_GLYR1_NM_032569	WIKVEQLKPYHAHK--EEMIK---INKGKRFQQAVDAVEE
54904_b_WHSC1L1_NM_023034_MSH6	WVHEKRVREYKGHKQYEELLAE--ATKQASNHSEKQKIRK
7468_b_WHSC1_NM_133330_MSH6l	WIFEKSLVAFEGEGQFEKLCQE--SAKQAPTKAEKIKLLK
64324_b_NSD1_NM_172349_MSH6l	WVAGKAIVMFEGRHQFEELP----VLRRRGKQKEK-GYRH
152098_ZCWPW2_NP_001035522	WIKATFVGHYSITLKPEKCKN-----KKKWYKSALQEA--
XP_001633052_Nv	WVNSFSVRELTADEEPEDIGNI--VVRKKNYKKQLDDANG
7468_a_WHSC1_NM_133330_WHSCr	WTHQARVFPYMEGDRGSRYQGV--RGIGRVFKNALQEAEA
64324_a_NSD1_NM_172349_WHSCr	WTHQARVFPYMEGDVSSKDKMG--KGVDGTYKKALQEAAA
54904_a_WHSC1L1_NM_023034_WHSC	WVHQGRVFPYVEGDK-SFAEGQ--TSINKTFKKALEEAAK
XP_001635219_Nv	WIHKGRVFSYQDGDKGTESGNN--KYLAKVFKKALVEAKE
XP_001747378_b_Mb	WVGHESIIPFSNGDQ-----------KNSYFTRNLKAAKK
23613_ZMYND8_NM_183047	WVPINNCYLMSKEIP-FSVKKT--KSIFNSAMQEMEVY--
10771_ZMYND11_NM_006624	WIPSENIQDITVNIHRLHVKRS--MG-WKKACDELELH--
27154_BRPF3_NM_015695	WLPRDKVLPLGVEDTVDKLKML--EGRKTSIRKSVQVAYD
7862_BRPF1_NM_001003694	WLPRTKLVPLGVNQDLDKEKML--EGRKSNIRKSVQIAYH
23774_BRD1_NM_014577	WLPKSKMVPLGIDETIDKLKMM--EGRNSSIRKAVRIAFD
114825_PWWP2Aisob_NP_001124336	FLALSQLSPFLENFQSRFNK-----KRKGLYRKAITEAAK
170394_PWWP2B_NM_138499	FLSISKLSPFSEFFKLRFNR-----KKKGMYRKAITEAAN
XP_001624451_Nv	EMPVQELQDFEPNFRRRYKK-----DKKGCYRRAVRQAQE
1788_DNMT3A_NM_022552	VVCVEKLMPLSS-FCSAFHQAT--YNKQPMYRKAIYEVLQ
1789_DNMT3B_NM_006892	EVSADKLVALGL-FSQHFNLAT--FNKLVSYRKAMYHALE
XP_001626231_Nv	QVTYDTVLPFAE-FKSNFLVTK--MKG--LYKKAVLDALE
NP_116242	TVSLKSLKHFDCKEKQTLLNQ-----AREDFNQDIGWC--
139221_MUM1L1_NM_152423	RVNFRRLKKFDCKEKQMLVDK-----AREDYSESIDWC--
728317_LOC728317_XP_001127165	QVPLRRLKHLDCKEKEKLLKR-----AQKAYKQSVNWC--
728307_LOC728307_XP_001127139	QVPLRRLKHLDCKEKEKLLKR-----AQKAYKQSVNWC--
55063_ZCWPW1_NM_017984	WIPVNMLKNFQELSLELSVMKK--RRNDCSQKLGVALMMA
XP_001750279_Mb	WVTSEQVMRMFASAWVDAQMLS--QTSKHAKDVFVRSLFE
2956_MSH6_HSU54777	WVSKRLLKPYTGSKSKEAQKGGHFYSAKPEILRAMQRADE

XP_003084095_O_tauri	WVREENCLDFSEHYESKSRDP---ARNKSRFQAALKQAND
XP_003079195_O_tauri	WVKKSKVYPFCTFYAEAMKQPM--FTARSTFTRMIDRA--

3068_HDGF_NP_004485	N------------------------
50810_HDGFRP3_CCDS32314	N------------------------
84717_HDGFRP2_UNQ785PRO1604	N------------------------
154150_HDGFL1_NP_612641	N------------------------
XP_001634069_Nv	N------------------------
84656_GLYR1_NM_032569	FL-----------------------
54904_b_WHSC1L1_NM_023034_MSH6	PRPQRERAQWDIGIAHAEKALKMTR
7468_b_WHSC1_NM_133330_MSH6l	PISGKLRAQWEMGIVQAEEAASMSV
64324_b_NSD1_NM_172349_MSH6l	KVPQKILSKWEASVGLAE-------
152098_ZCWPW2_NP_001035522	-------------------------
XP_001633052_Nv	QARQALSLPVQERLAV---------
7468_a_WHSC1_NM_133330_WHSCr	RFREI--------------------
64324_a_NSD1_NM_172349_WHSCr	RFEEL--------------------
54904_a_WHSC1L1_NM_023034_WHSC	RFQEL--------------------
XP_001635219_Nv	KYDEW--------------------
XP_001747378_b_Mb	SFQE---------------------
23613_ZMYND8_NM_183047	-------------------------
10771_ZMYND11_NM_006624	-------------------------
27154_BRPF3_NM_015695	RAMIH--------------------
7862_BRPF1_NM_001003694	RALQH--------------------
23774_BRD1_NM_014577	RAMNH--------------------
114825_PWWP2Aisob_NP_001124336	AA-----------------------
170394_PWWP2B_NM_138499	AA-----------------------
XP_001624451_Nv	TL-----------------------
1788_DNMT3A_NM_022552	VA-----------------------
1789_DNMT3B_NM_006892	KA-----------------------
XP_001626231_Nv	VL-----------------------
NP_116242	-------------------------
139221_MUM1L1_NM_152423	-------------------------
728317_LOC728317_XP_001127165	-------------------------
728307_LOC728307_XP_001127139	-------------------------
55063_ZCWPW1_NM_017984	QEAEQISI-----------------
XP_001750279_Mb	-------------------------
2956_MSH6_HSU54777	ALNKDKIKRLELAV-----------
XP_003084095_O_tauri	E------------------------
XP_003079195_O_tauri	-------------------------
