# Supplementary material for: Evolution of the PWWP-domain encoding genes in the plant and animal lineages
Source: BMC Evol Biol. 2012 Jun 26;12:101. doi: 10.1186/1471-2148-12-101 (PMC3457860; doi:10.1186/1471-2148-12-101)

Additional file 7. Gene architecture of the PWWP domain containing proteins from the 12 genomes studied

Symbols	Domain names	Symbols	Domain names
	PWWP domain		SET domain
0	PHD Plant homeo domain		DAST Domain Associated with SET in Trithorax
◊	Bromo_RACK7 Bromodomain	&&&	VHS_ENTH_ANTH VHS, ENTH and NTH domain superfamily
###	EPL1 Enhancer of polycomb-like domain		AdoMet_MTases S-adenosylmethionine-dependent methyltrans-ferases domain
	MDN1 AAA ATPase containing von Willebrand factor type A (vWA) domain		Dcm Site-specific DNA methylase
	Chromatin binding site		AWS domain associated with SET
	MutS Mismatch repair ATPase domain		HMG High Mobility Group (HMG)-box
	MmsB 3-hydroxyisobutyrate and related beta-hydroxyacid dehydrogenases		FATC FATC domain
	zf-CW CW-type Zinc Finger		PIKKc_ATM Ataxia telangiectasia mutated catalytic domain
	Peptidase C12 Cysteine peptidase C12 containing domain		SAND SAND domain
	Chromo Chromatin organization modifier domain		TUDOR Tudor domain
++++	SNF2_N SNF2 family N-terminal domain		Tudor/PWWP/MBT superfamily domain
	HELICc Helicase superfamily c-terminal domain		Rubis-s Rubisco LSMT substrate-binding
	APC APC family of proteins		

In the following figures, each protein is represented by a rectangle proportional to the length. Positions of domains are illustrated using the symbols described above. The sizes of these symbols are not proportional to their actual size. When some regions are abbreviated for too long proteins, such regions are indicated with the following start and end position: Start End

solo-PWWP domain proteins.

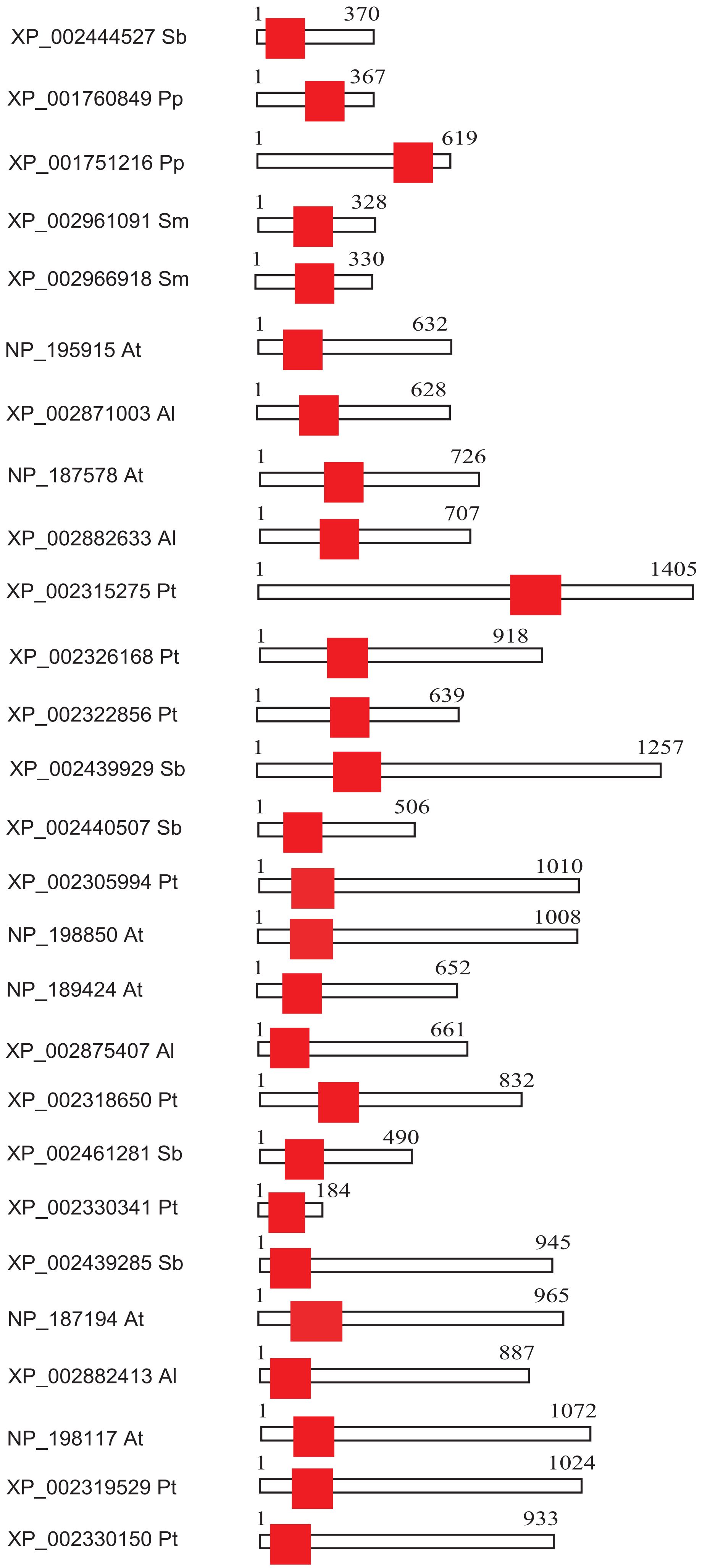

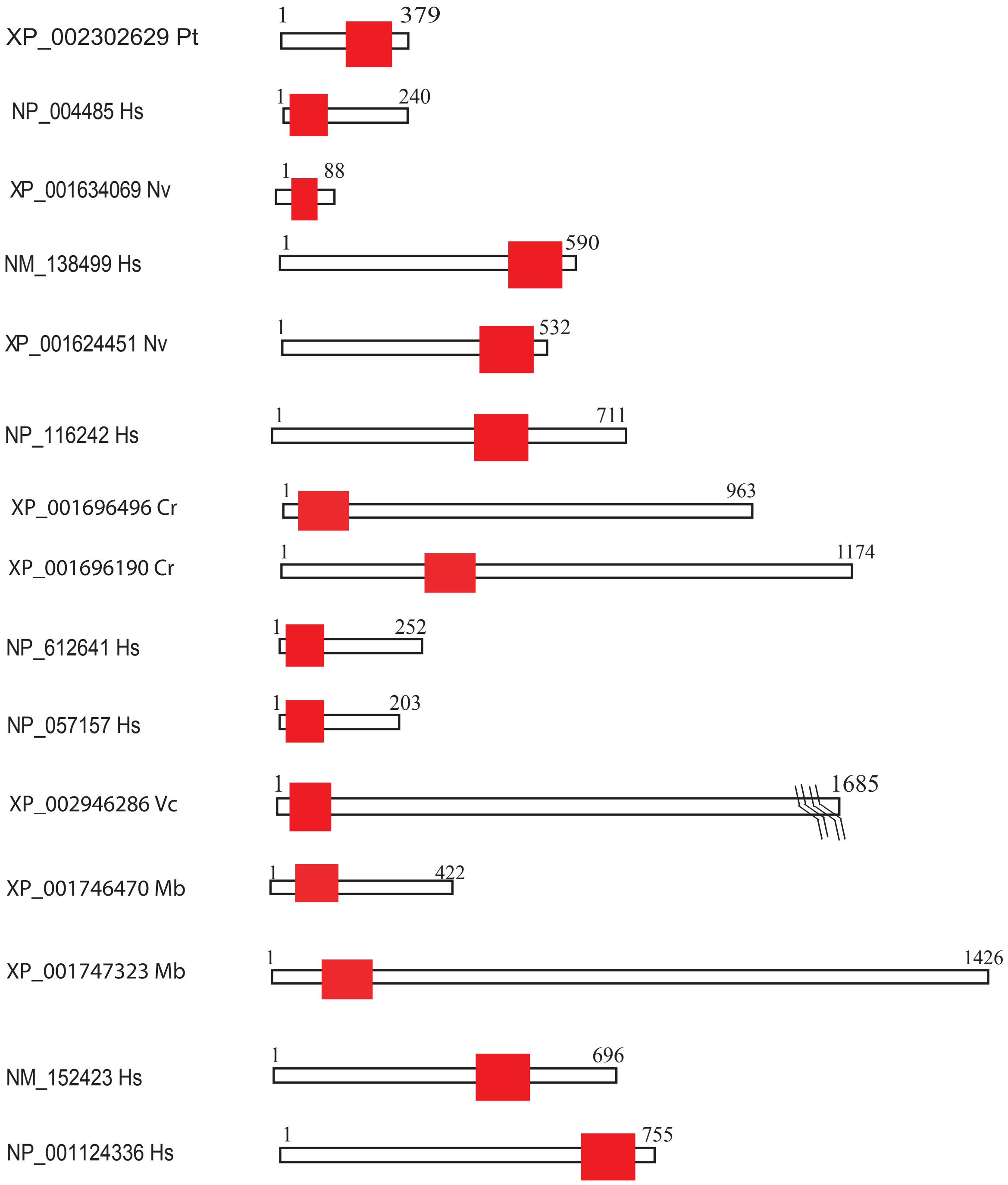
XP_002302629 Pt
1
379
NP_004485 Hs
1
240
XP_001634069 Nv
1
88
NM_138499 Hs
1
590
XP_001624451 Nv
1
532
NP_116242 Hs
1
711
XP_001696496 Cr
1
963
XP_001696190 Cr
1
1174
NP_612641 Hs
1
252
NP_057157 Hs
1
203
XP_002946286 Vc
1
1685
XP_001746470 Mb
1
422
XP_001747323 Mb
1
1426
NM_152423 Hs
1
696
NP_001124336 Hs
1
755

The PWWP domain in species- or lineage-specific combinations

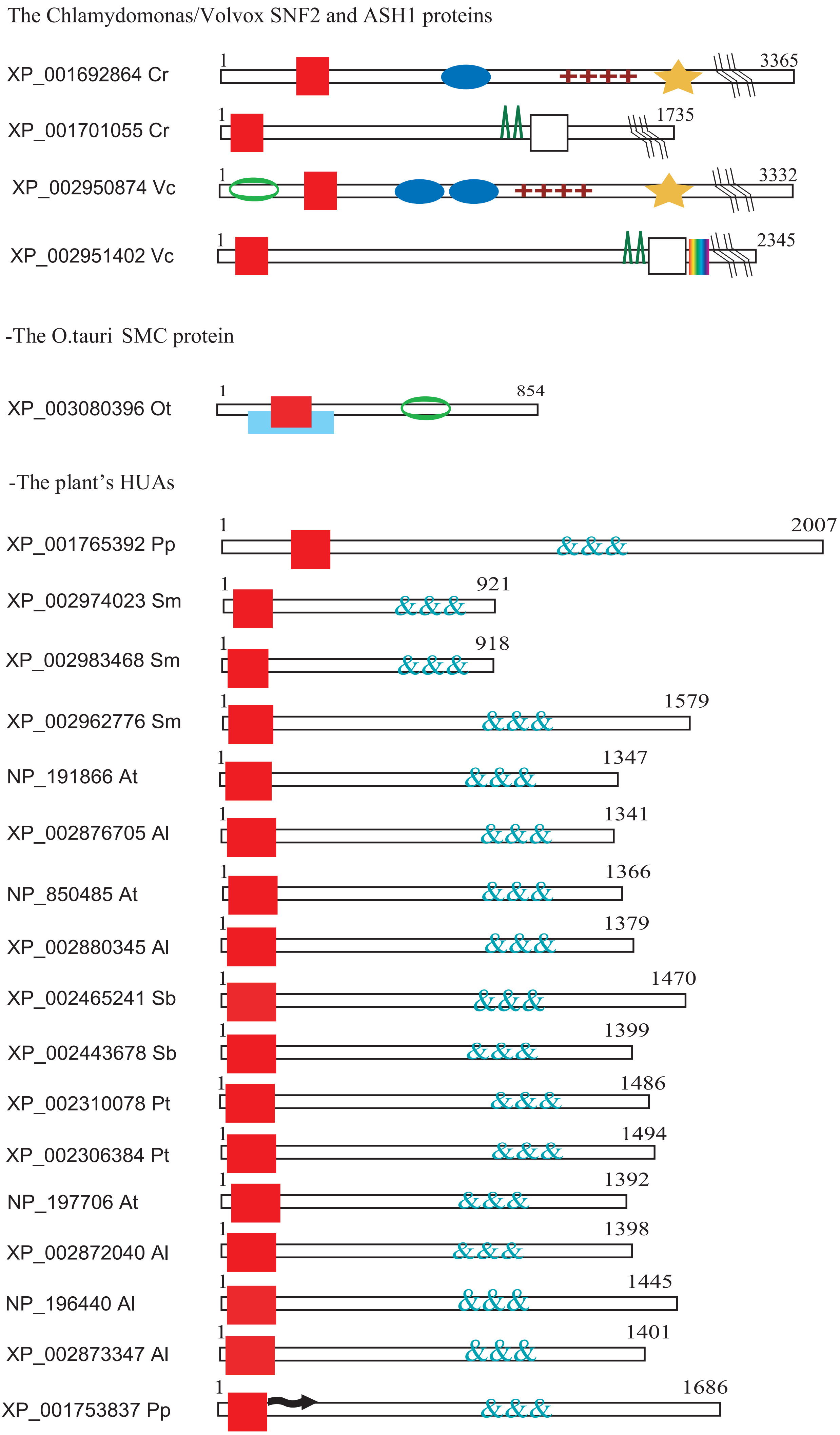

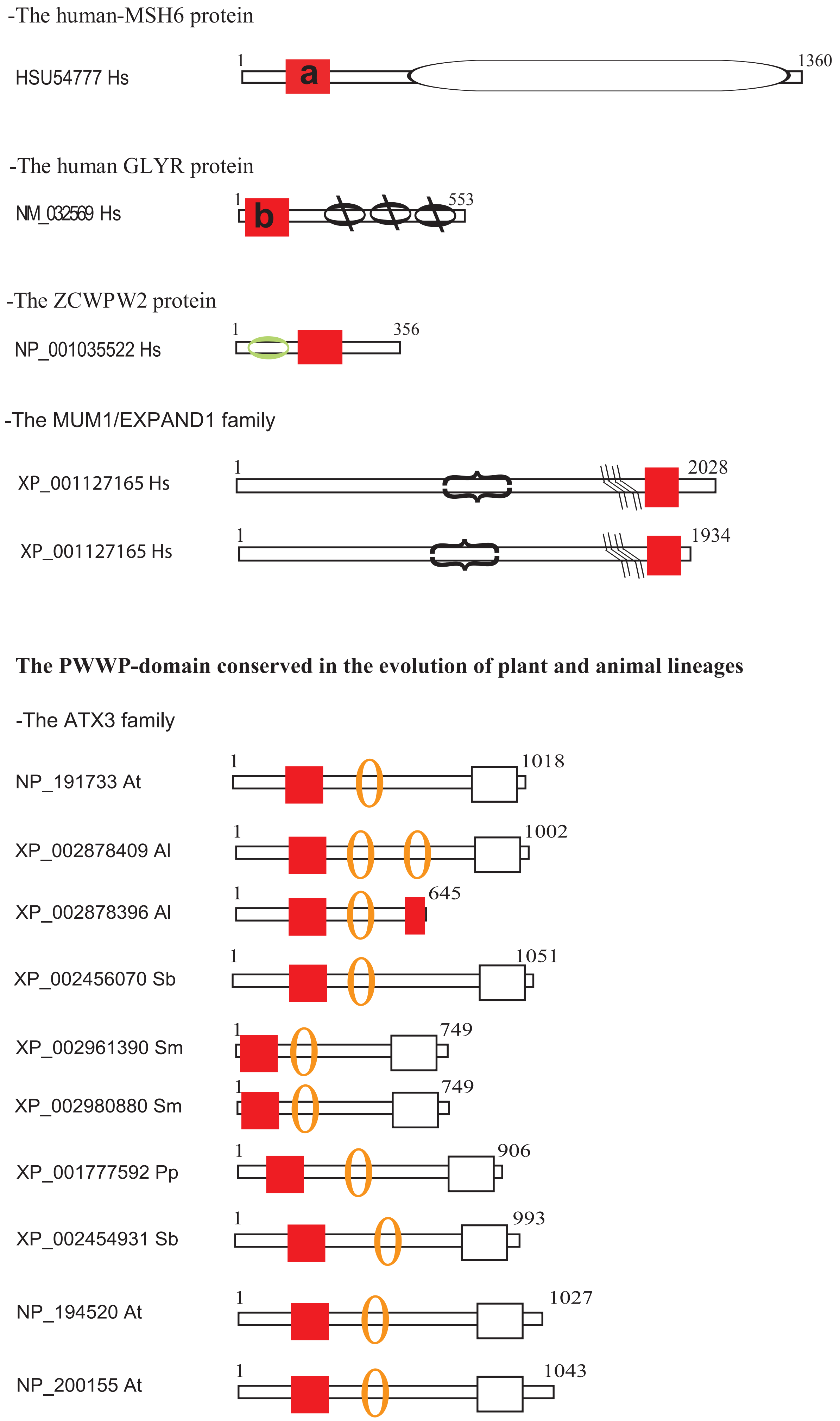

-The human-MSH6 protein
HSU54777 Hs
1
a
1360
-The human GLYR protein
NM_032569 Hs
1
b
553
-The ZCWPW2 protein
NP_001035522 Hs
1
356
-The MUM1/EXPAND1 family
XP_001127165 Hs
1
2028
XP_001127165 Hs
1
1934
The PWWP-domain conserved in the evolution of plant and animal lineages
-The ATX3 family
NP_191733 At
1
1018
XP_002878409 Al
1
1002
XP_002878396 Al
1
645
XP_002456070 Sb
1
1051
XP_002961390 Sm
1
749
XP_002980880 Sm
1
749
XP_001777592 Pp
1
906
XP_002454931 Sb
1
993
NP_194520 At
1
1027
NP_200155 At
1
1043

-The ATX1 family

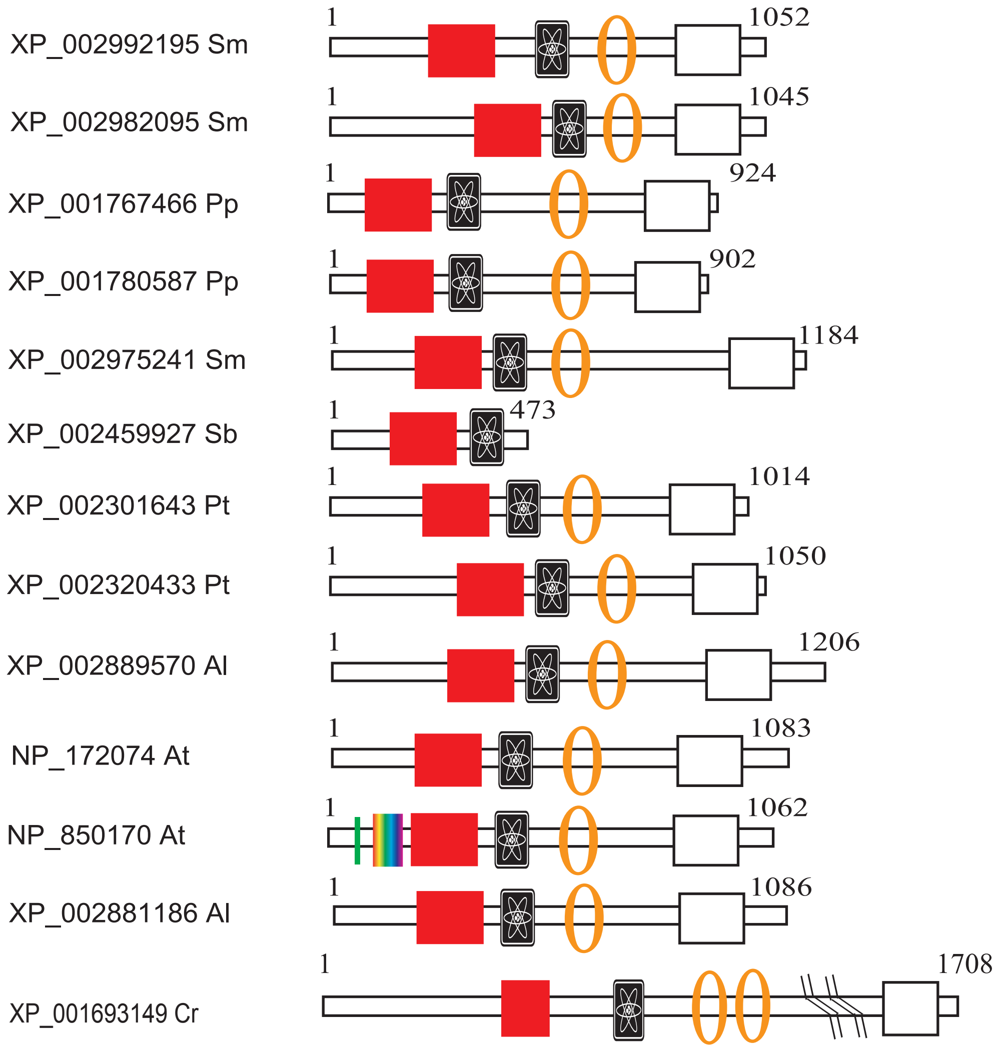

-The Peregrin-type or Brefeldin family

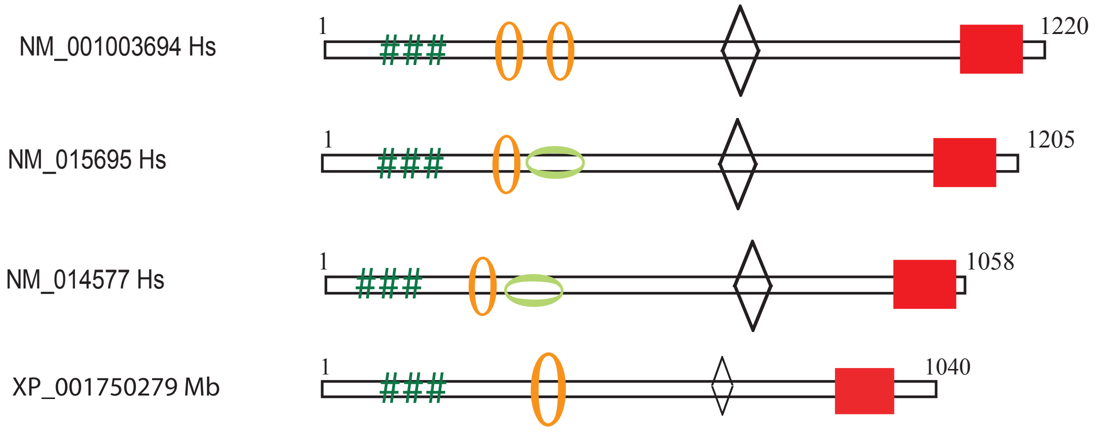

-The WHSC family

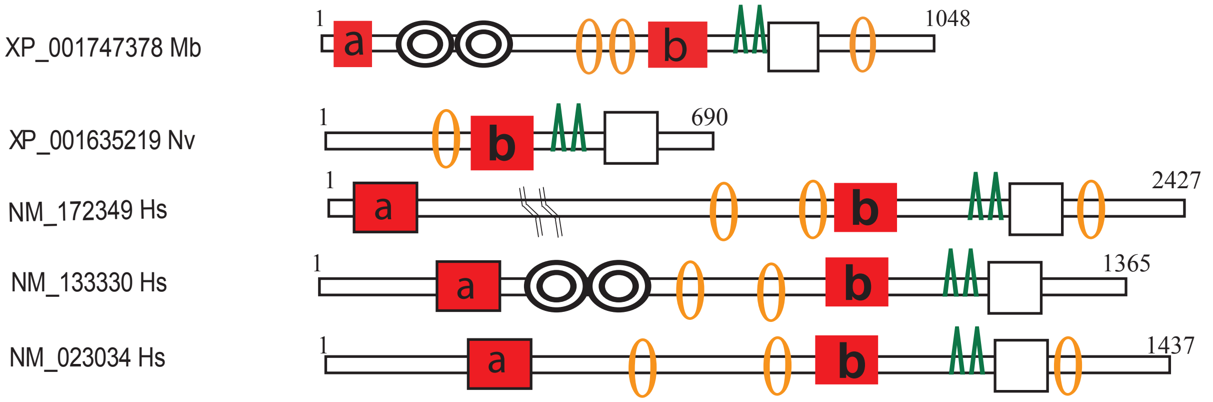

-The Z/MYND family

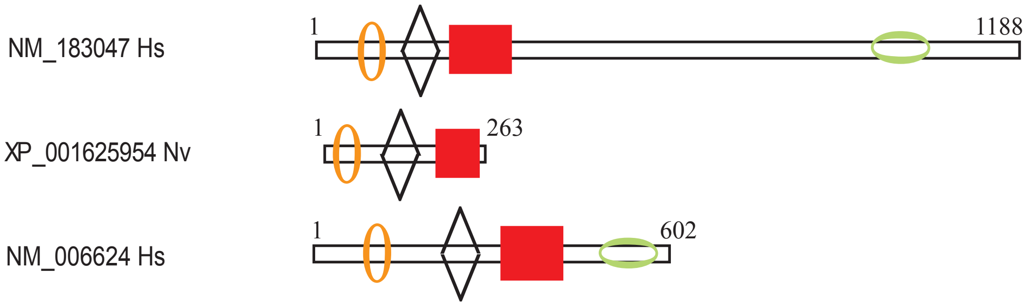

-The ZCWPW1 family

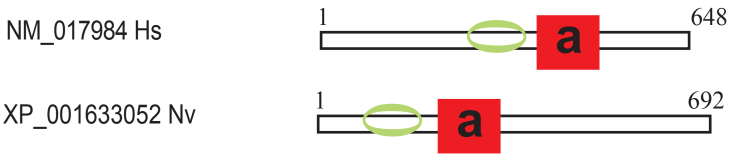

-The DNA methyltransferases

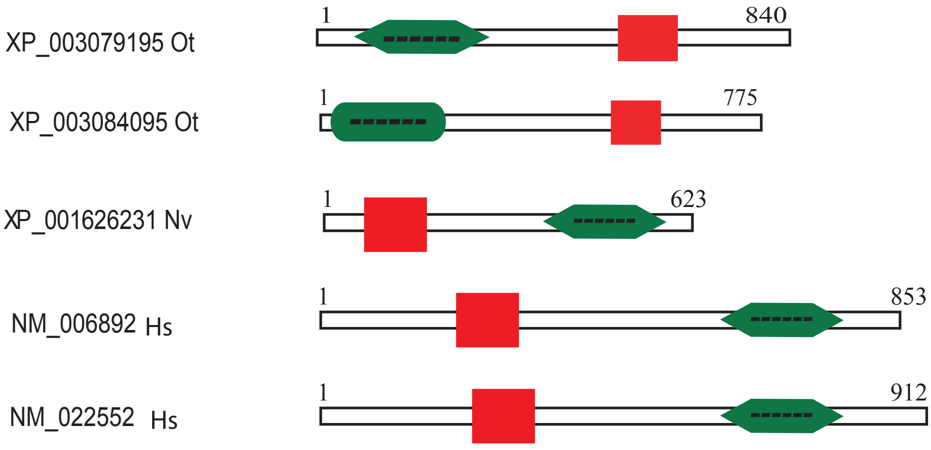

Supplement: Additional file 7 — Gene architecture of the PWWP domain containing proteins from the 12 genomes studied. [file 1471-2148-12-101-S7.pdf]
