# Supplementary material for: Evolution of the PWWP-domain encoding genes in the plant and animal lineages
Source: BMC Evol Biol. 2012 Jun 26;12:101. doi: 10.1186/1471-2148-12-101 (PMC3457860; doi:10.1186/1471-2148-12-101)
Supplement: Additional file 8 — Neighbor-Joining phylogeny of PWWP containing proteins in plants including the ATX3-like protein fromO. tauri and the ATX1-like protein of Chlamydomonas. [file 1471-2148-12-101-S8.pdf]

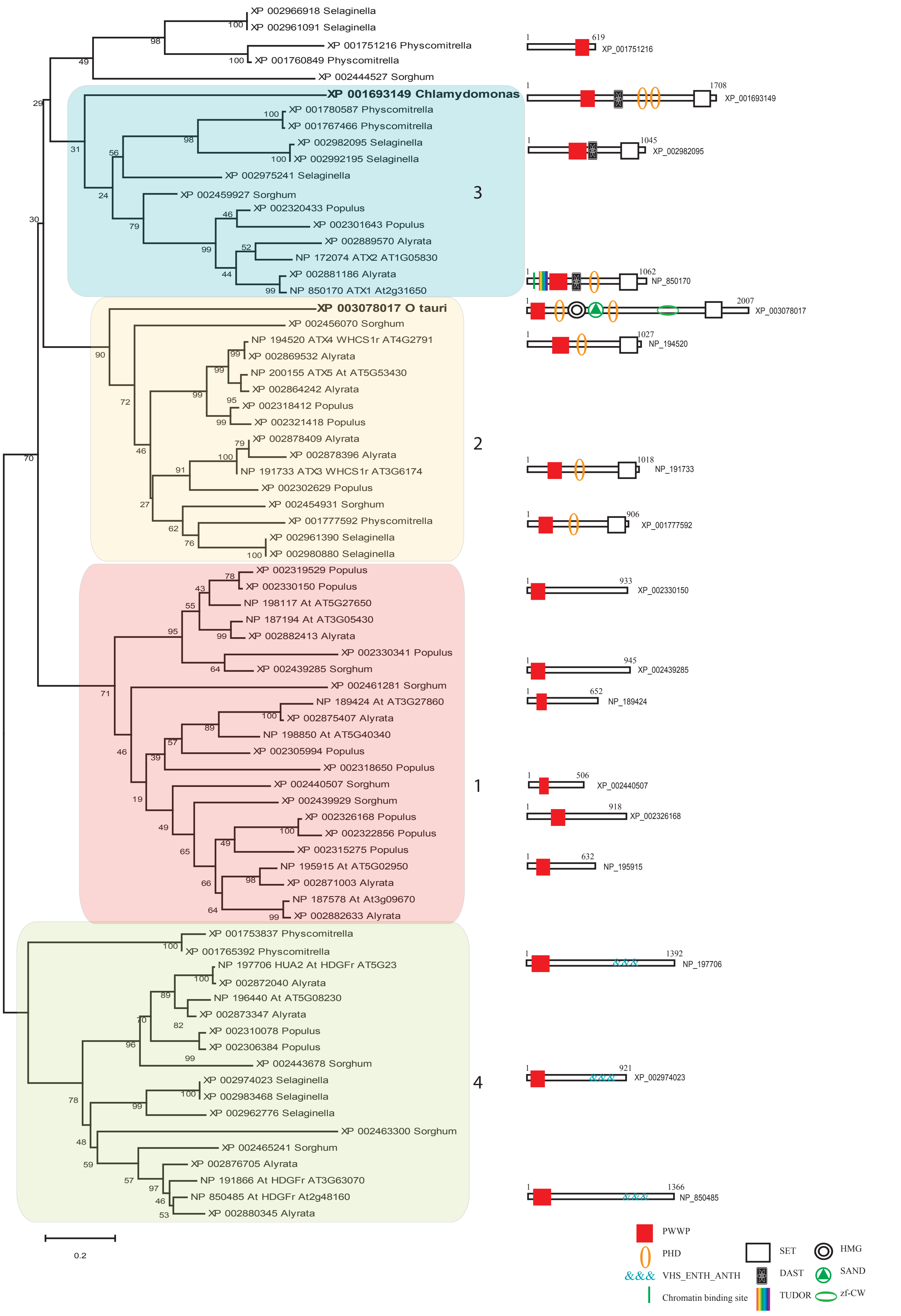

Additional File 8. Neighbor-Joining phylogeny of PWWP containing proteins in plants including the ATX3-like protein from *O. tauri* and the ATX1-like protein of Chlamydomonas. The tree was constructed as described in Additional file 5. Phylogenetic analyses were conducted in MEGA4.
