# Supplementary material for: Evolution of the PWWP-domain encoding genes in the plant and animal lineages
Source: BMC Evol Biol. 2012 Jun 26;12:101. doi: 10.1186/1471-2148-12-101 (PMC3457860; doi:10.1186/1471-2148-12-101)
Supplement: Additional file 9 — Neighbor-Joining phylogeny of PWWP containing proteins in humans, Nematostella, M. brevicolis and O. tauri . [file 1471-2148-12-101-S9.pdf]

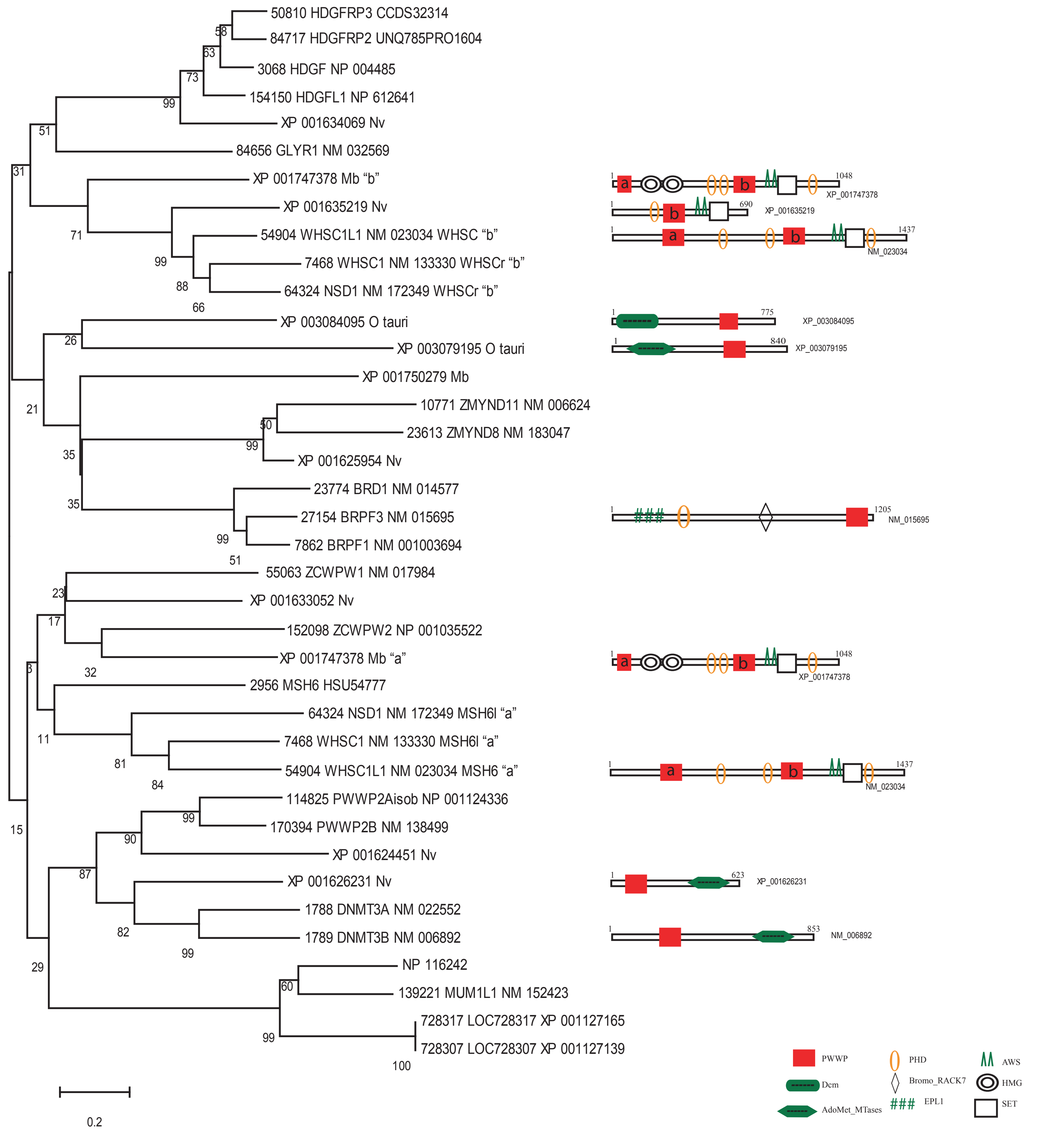

Additional File 9. Neighbor-Joining phylogeny of PWWP containing proteins in humans, Nematostella, *M. brevicolis* and *O. tauri*. The evolutionary history was inferred using the Neighbor-Joining method. The percentage of replicate trees in which the associated taxa clustered together in the bootstrap test (5000 replicates) are shown next to the branches. The tree is drawn to scale, with branch lengths in the same units as those of the evolutionary distances used to infer the phylogenetic tree. The evolutionary distances were computed using the Poisson correction method. All positions containing gaps and missing data were eliminated from the dataset (Complete deletion option). Phylogenetic analyses were conducted in MEGA4. All positions containing gaps and missing data were eliminated from the dataset (Complete deletion option). Phylogenetic analyses were conducted in MEGA4. The PWWP domains marked as PWWPa and PWWb correspond to the two PWWP domain subtypes (see text for details). The distinct PWWP-domain subgroups (as discussed in the main text) are shaded in different colors. Species abbreviations correspond to *Nematostella vectensis* (Nv), *Monosiga brevicolis* (Mb), *Ostreococcus tauri* (O. tauri) and the rest of the proteins correspond to humans.
